# Supplementary material for: Study protocol: establishment of a multicentre pre-eclampsia database and biobank in Sweden: GO PROVE and UP MOST, a prospective cohort study
Source: BMJ Open. 2021 Nov 24;11(11):e049559. doi: 10.1136/bmjopen-2021-049559 (PMC8614148; doi:10.1136/bmjopen-2021-049559)
Supplement: Supplementary data [file bmjopen-2021-049559supp001.pdf]

Table S1. Variables included in the study.

**Variables from the woman's medical chart**	**Variables from the study inclusion visit, gathered from the patients´ medical charts**	**Variables from the study inclusion visit when diagnosed with preeclampsia, registered by patients in the enrolment module**	**Variables from the Swedish Pregnancy Register after delivery**
Marital status (n)<br>Current living situation (n)<br>Country of birth (n)<br>Total years of education (c)<br>Profession (n)<br>Maternal occupation in the beginning of pregnancy (b)<br>Height (c)<br>Intra uterine growth restriction (b)<br>Preeclampsia (b)<br><br>**Obstetric history**<br>Number of pregnancies terminated before 21+6 weeks gestation (d)<br>Previous pregnancies, terminated before 21+6 weeks gestation, causes (n)<br>Gestational age (d)<br>Gestational hypertension (b)<br>Preeclampsia (b)<br>Eclampsia (b)<br>HELLP (b)<br>Gestational diabetes treated with Insulin or oral agents (b)<br>Treatment details (Free text)<br><br>Number of pregnancies beyond 21+6 weeks gestation (d)<br>Information on previous pregnancies, with delivery beyond 21+6 weeks gestation, outcome details:<br>Gestational age (d)<br>Gestational hypertension (b)<br>Preeclampsia (b)<br>Eclampsia (b)<br>HELLP (b)	**Study assessment at inclusion**<br>Date (c)<br>Location (n)<br>1st BP (c)<br>2nd BP (c)<br>Pulse (c)<br>Weight (c)<br>Proteinuria (dipstick) (o)<br>Urinary protein/creatinine ratio (c)<br>24 hour protein (c)<br>Gestation at PE/Intra uterine growth restriction (IUGR) diagnosis (c)<br><br>**Investigations at inclusion**<br>Samples taken, see table 3<br><br>**Urine sample**<br>See Table 3<br><br>**Gestational diabetes (GDM) screening, diagnosis treatment**<br>Date screened (c)<br>Date of GDM diagnosis (c)<br>Gestation GDM diagnosed (c)<br>Date of glucose tolerance test (d)<br><br>**Ultrasound and doppler scan after preeclampsia diagnosis**<br>Date of scan (d)<br>Fetal sex (n)<br>Gestational age when scanned (c)<br>Fetal abnormality (n)<br>Crown rump length (c)<br>Nuchal translucency (c)	Yearly income (o)<br>First pregnancy with the baby's biological father (b)<br>Months of sexual relationship (pre-pregnancy) (c)<br>Contraception used in this relationship (n)<br>If contraception has been used, which mostly in the last year (n)<br>Medications before pregnancy (n)<br>First degree relative/s with hypertensive disease in pregnancy (b)<br>First degree relative/s with hypertension, Ischaemic Heart Disease, Stroke or any form of Diabetes (b)<br>Pre-pregnancy weight (c)<br><br>**Partners Family History**<br>Children with other partners (b)<br>Pregnancy outcome with other partner (n)<br>First degree relative with preeclampsia or Eclampsia (b)<br>First degree relative/s with Hypertension (b), Ischaemic Heart Disease (b), Stroke (b) or any form of Diabetes (b)<br><br>**Partner's Data**<br>Age (c)<br>Weight (c)<br>Height (c)<br>Body mass index (c)<br>Partners medical condition (n)<br>Diabetes mellitus (b)<br>Hypertension (b)<br>Chronic Heart Disease (b)	**Maternal**<br>*Delivery data*<br>Date of admission pre-delivery (c)<br>GA at admission pre-delivery (c)<br>Onset of delivery (n)<br>Maximum temperature in labour (c)<br><br>Foetal compromise pre-labour Caesarean section<br>Place and supervision of delivery (n)<br>Duration of rupture of membranes (c)<br><br>Duration of 1st stage (c)<br>Duration of 2nd stage (c)<br><br>Mode of delivery (n)<br>Indication for operative delivery (n)<br>Indication for delivery (n)<br><br>*Medication*<br>Magnesium sulphate (n)<br>Steroids (n)<br>Antihypertensive treatment in labour (n)<br>Medication during labour and delivery only (Free text)<br>Calcium antagonist (b)<br><br>*Placenta*<br>Recommended standards of placental handling used (b)<br>Placental weight (c)<br>Cord insertion relative to placental margin (n)<br>Number of vessels in cord (b)<br>Pathology report (b)

Gestational diabetes treated with Insulin or oral agents (b)<br>Treatment details (n)<br><br>**History of infertility** (b)<br>Fertility treatment for this conception (b)<br>Stimulation for ovulation for this conception (b)<br>Artificial insemination for this conception (n)<br>In Vitro fertilization for this conception (n)<br>Intra Cytoplasmic sperm injection for this conception (n)<br>Embryo recipient for this conception (b)<br><br>**Medical conditions diagnosed before pregnancy**<br>Polycystic ovary syndrome (b)<br>Medical conditions diagnosed before pregnancy (n)<br>Hypertension (b)<br>Renal disease (b)<br>Anaemia (b)<br>Diabetes type I (b)<br>Diabetes type II (b)<br>Lupus disease (b)<br>Inflammatory bowel disease (b)<br>Coeliac disease (b)<br>Rheumatoid arthritis (b)<br>Epilepsy (b)<br>Thromboembolism (b)<br>History of depression (b)<br>Antiphospholipid syndrome (b)<br>HIV (b)<br>Sickle cell (b)<br>Malaria requiring treatment in the last year (b)<br>Asthma (b)<br>Oral steroids for asthma (b)<br>Thyroid disease (n)<br>Migraine (b)<br>Irritable colon (b)<br>Anxiety disorder (b)<br>Other medical conditions diagnosed before pregnancy (free text)	Biparietal diameter (BPD (c)<br>Head circumference (HC) (c)<br>Abdominal diameter (AD (c)<br>Femur length (C)<br>Sub-chorionic haematoma (b)<br>2nd sac (b)<br>Risk score of Trisomy 21(d)<br>Liquor volume (o)<br>Placental Location (n)<br>Clinical suspicion for fetal growth restriction (b)<br>Documented fetal growth restriction (b)<br>Clinical indication for Macrosomia (b)<br>Amniotic fluid index (c)<br>Single deepest pocket (c)<br><br>*Umbilical dopplers* (c)<br>Gestational age (c)<br>Umbilical PI (c)<br><br>*Uterine dopplers*<br>L Ut Pulsatility Index (c)<br>R Ut Pulsatility Index (c)<br>Mean Ut PI (c)<br>L Notch (c)<br>R Notch (c)<br>Other Ultrasound comments (Free text)<br>Fetal Outcome (n)<br><br>**Medication use at inclusion**<br>Any medication at inclusion(b)<br>Low dose aspirin (b)<br>Other antiplatelet medication (b)<br>Low molecular weight heparin (n)<br>Diuretics (n)<br>Labetalol (n)<br>Beta blocking agent (n)<br>Calcium channel antagonist (n)<br>Antibiotic (n)<br>Anticoagulants (n)<br>Anticonvulsant-1 (not $MgSO_4$) (n)<br>Antidepressants (n)	Other (Free text)<br><br>**Vitamin and mineral supplementation before /and during pregnancy**<br>Folate (c)<br>Multivitamin (o)<br>Vitamin D (o)<br>Iron (o)<br>Calcium (o)<br><br><br>**Infection in pregnancy to record** (b)<br>Type of infection (n)<br>Urinary tract infection (lower) (b)<br>Pyelonephritis (b)<br>Human immunodeficiency virus (b)<br>Active Tuberculosis (b)<br>Zika virus (b)<br>Schistosomiasis (b)<br>Active or chronic hepatitis (b)<br>Active or recently treated sexually transmitted infection (b)<br>Other infection in pregnancy (b)<br><br>Diagnosis (n)<br>Treatment (n)<br><br>**Symptoms at inclusion see Table X**<br><br>**At time of MRI brain and Cognitive tests**<br>Blood pressure (c)<br>Pulse (c)<br>Oxygen saturation (d)<br><br>Current treatment<br>-Magnesium sulphate IVI (b)<br>-Labetolol IVI (b)<br>-Hydralazine IVI (b)<br>-Calcium antagonist short acting oral (b)<br>-Labetolol oral (b)<br>-Calcium antagonist long acting oral (b)	Photograph against a scale bar (b)<br><br>**Newborn data**<br>Liveborn (b)<br>Date of birth (c)<br>Time of birth (c)<br>Gestational age at delivery (c)<br>Sex (b)<br>Apgar score (d)<br>Intubation at birth (n)<br>Cord arterial pH (c)<br>Cord arterial Base deficit (c)<br>Cord venous pH (c)<br>Cord venous Base deficit (c)<br>Birthweight (c)<br>Birthweight centile (c)<br>Customised birthweight centile (c)<br>Length (c)<br>Head circumference (c)<br>TSH (c)<br>T3 total (c)<br>T4 total (c)<br>T3 free (c)<br>T4 free (c)<br>ICD code(s) for measures and diagnosis (n/free text)<br><br>*Neonatal Outcome at discharge*<br>Uncomplicated neonatal outcome (b)<br>Neonatal outcome (n)<br>Date of discharge home (c)<br>Days in hospital from birth to discharge home (d)<br>Admission to neonatal unit (c)<br>Final discharge date from neonatal unit or date of death in neonatal unit (c)<br>Days in neonatal unit (d)<br><br>**Perinatal outcome**<br>Perinatal Survival (b)<br>Stillbirth (b)

Medications before pregnancy (n)

Last menstrual period (LMP)
Certainty (n)
LMP date (c)
Gestational age by LMP/clinical examination (c)
Estimated due date by LMP (c)

Estimated due date estimated by scan
<16 week scan date (c)
19-21 week scan date (c)
Final estimated due date (c)

Clinical Readings at 1st Clinical Assessment
Date of 1st clinical blood pressure (BP) (c)
Gestation of 1st BP (c)
1st clinical weight (c)
Proteinuria dipstick (o)
Urinary protein/creatinine ratio (c)
24 hour protein (c)

Clinical Test
(*Prenatal screening for chromosomal anomalies*)
Prenatal diagnosis (n)
Serum Screening for prenatal diagnosis (SSPD) (b)
Date of SSPD performed (c)
Gestation if SSPD performed (c)

1st and 2nd Trimester Complications
Hyperemesis (b)
Treatment in case of hyperemesis (free text)
Vaginal bleeding (b)
Gestational week if vaginal bleeding has occurred (c)
Duration of bleeding (d)
Amount of blood (o)

Antiglycemic agent –Insulin (b)
Metformin (b)
Long-term corticosteroids (b)
Other immunosuppressant (n)
Thyroid supplement (b)
Antithyroid treatment for thyrotoxicosis (b)
Statins (n)
Other medication(n)
Hydralazine (b)
Magnesium sulphate (b)

Received steroids for fetal lung maturation before sampling (b)
Type of steroids (n)
Date started (c)
Gestational age started (c)
Date completed (c)
Gestational age completed (c)
Total dose (c)
Time between last dose and sampling (c)

-ACE inhibitor oral (b)
-AT-2 inhibitor oral (b)
-Diuretics IVI (b)
-Diuretics oral (b)
-Other medications (b)

Neurological symptoms at MRI brain, see table X

Echocardiocraphy and RH pat
Date (c)
Blood pressure (c)
Pulse (c)
Oxygen saturation (d)
Oxygen administration (b)
Current treatment:
-Magnesium sulphate IVI (b)
-Labetolol IVI (b)
-Hydralazine IVI (b)
-Calcium antagonist short acting oral (b)
-Labetalol (b)
-Calcium antagonist long acting oral (b)
-ACE inhibitor oral (b)
-AT-2 inhibitor oral (b)
-Diuretics IVI (b)
-Diuretics oral (b)
-Other medications (b)
If *other*, state (FREE text)

Variables from the visit at 1 year postpartum registered by the patient in the study module as well as study personnel
Date (date)
Blood pressure (c)
Pulse (c)
Weight (c)
Medication
ACE inhibitor (b)
AT-2 inhibitor (b)
Diuretics (b)

Neonatal death (o)

Final Maternal and Perinatal Outcome
Maternal outcome (n)
Maternal admission to intensive care unit (b)

Pregnancy Summary & Complication
Did any of these complications occur
-Normal pregnancy (b)
-Preeclampsia (b)
-Gestational Hypertension (b)
-Eclampsia or HELLP (b)
-Chronic hypertension (b)
-Placental abruption (b)
-Maternal death (b)
-Small for gestational age by population centile (b)
-Fetal growth retardation (b)
-Spontaneous preterm birth (b)
-PPROM with spontaneous preterm birth (b)
-Cervical insufficiency (b)
-Pregestational diabetes (b)
-Gestational diabetes (b)
-Cholestasis of pregnancy (b)
-Venous thromboembolism (b)
-Obese (BMI≥30) (b)
-Postpartum haemorrhage (PPH) (b)
-Other complications (b)
Maternal outcome comment (Free text)
-Perinatal outcome (n)
Fetal outcome comment (Free text)
Autopsy examination (n)
Major congenital abnormalities (n)
Chromosomal abnormality (n)
Congenital infection (b)
ICD codes for diagnosis
O14.0 (b)
O14.1A (b)
O14.1B (b)
O14.1X (b)
O14.9 (b)

**Recreational Drug use**<br>Any recreational drug user (n)<br>Gestation ceased (c)<br>Tobacco (o)<br>Cigars (o)<br>Chewing tobacco (o)<br>Snuff (o)<br>E-cigarettes (o)<br>Nicotine replacement (o)<br>Alcohol (o)<br><br>*Other drugs in pregnancy*<br>Marijuana (b)<br>Cocaine/Crack (b)<br>Amphetamines (b)<br>Substance P/Crystal Meth (b)<br>XTC (b)<br>Opiates (b)<br>Hallucinogens (b)<br>Other (n)<br><br>Audit number 3 months prior to pregnancy (d)<br><br>**Ultrasound**<br>Date of scan (c)<br>Fetal sex (n)<br>Gestational age (c)<br>Fetal abnormality(n)<br>CRL (crown rump length) (c)<br>BPD (biparietal diameter) (c)<br>HC (head circumference) (c)<br>AD (abdominal diameter) (c)<br>FL (femur length) (c)<br>Sub-chorionic haematoma(b)<br>2nd sac (b)<br>Risk score of Trisomi 21(d)<br>Liquor Volume (o)<br>Placental Location (n)<br>Clinical suspicion for fetal growth restriction (FGR) (b)<br>Documented fetal growth restriction (b)		Betablocker (b)<br>Aspirin (b)<br>Insulin (b)<br>Oral diabetes medication (b)<br>Lipid lowering medication (b)<br>Levothyroxine (b)<br>Antidepressants (b)<br>Sleep medicine (b)<br><br>Did she breastfeed (b)<br>Period of breastfeeding (o)<br>Regained period (b)<br>Use of contraception (b)<br>-If yes, what contraception (free text)<br><br>After delivery, has the woman sought healthcare for;<br>-Migraine (b)<br>-High blood pressure (b)<br>-Visual disturbance (b)<br>-Diabetes (b)<br>-Thyroid function disorder (b)<br>-Allergies (b)<br>-Irritable colon (b)<br>-Alcohol or drug addiction (b)<br>-Depression (b)<br>-Anxiety (b)<br>-Pain-related disorders (b)<br><br>Cognitive test performed (b)<br>-STAI, PANAS, PSQI performed (b)<br>-EPDS, HADS performed (b)<br>-EQ-VAS, CEC, BSES, GES performed (b)<br>-Bioimpedance been performed (b)<br><br>**Neurological symptoms at 1 year postpartum, see Table X**	O11.9 (b)<br>Other (b)<br>Procedure codes (FREE text)<br><br>**Other severe complication at any time before or after delivery**<br>-None (b)<br>-Unknown (b)<br>-DIC (b)<br>-Acute renal insufficiency (b)<br>-No urine catheter output for more than 60 min (b)<br>-Haemolysis (b)<br>-CVA (b)<br>-Epigastric pain (b)<br>-Pulmonary Oedema (b)<br>-Hepatic rupture (b)<br>-Severe sepsis (b)<br>-Maternal death (b)<br>-Foetal death (b)<br>-Thrombocytopenia (b)<br>-Abnormal LFT (due to PE) (b)<br>-Coma (b)<br>-Jaundice (b)<br>-Respiratory failure (b)<br>-Cardiac failure (b)<br>-Venous Thromboembolism (b)<br>-Other complications (b)<br>-If other, specify (FREE text)<br><br>**Summary of antenatal and postnatal lab results**<br>-Blood group (n)<br>-Rhesus factor (b)<br>Table Y<br><br>**End report-status**<br>Final pregnancy outcome (n)<br><br>If pregnancy ended $\leq 19^{+6}$, date (c)

Clinical indication for Macrosomia (b)<br>Amniotic fluid index (AFI) (c)<br>Single deepest pocket (c)<br><br>*Umbilical dopplers*<br>Gestational age (c)<br>Umbilical RI (resistive index) (n)<br>Umbilical PI (pulsatility index) (n)<br>Umbilical vein (n)<br><br>*Uterine dopplers*<br>L Ut PI (n)<br>R Ut PI (n)<br>Mean Ut PI (n)<br>L notch (n)<br>R notch (n)<br>Foetal outcome (n)			Gestational age at pregnancy end (c)<br><br>**MRI brain 1 year postpartum**<br>Blood pressure (c)<br>Pulse (c)<br>Plasma sample taken (b)
