# Supplementary material for: Study protocol: establishment of a multicentre pre-eclampsia database and biobank in Sweden: GO PROVE and UP MOST, a prospective cohort study
Source: BMJ Open. 2021 Nov 24;11(11):e049559. doi: 10.1136/bmjopen-2021-049559 (PMC8614148; doi:10.1136/bmjopen-2021-049559)
Supplement: Author's manuscript [file bmjopen-2021-049559.draft_revisions.pdf]

BMJ Open is committed to open peer review. As part of this commitment we make the peer review history of every article we publish publicly available.

When an article is published we post the peer reviewers' comments and the authors' responses online. We also post the versions of the paper that were used during peer review. These are the versions that the peer review comments apply to.

The versions of the paper that follow are the versions that were submitted during the peer review process. They are not the versions of record or the final published versions. They should not be cited or distributed as the published version of this manuscript.

BMJ Open is an open access journal and the full, final, typeset and author-corrected version of record of the manuscript is available on our site with no access controls, subscription charges or pay-per-view fees (http://bmjopen.bmj.com).

If you have any questions on BMJ Open's open peer review process please email info.bmjopen@bmj.com

BMJ Open

# Establishment of a multi-center preeclampsia database and biobank in Sweden – GO PROVE and UP MOST, a prospective cohort study



| | |
|---|---|
| Journal: | *BMJ Open* |
| Manuscript ID | bmjopen-2021-049559 |
| Article Type: | Protocol |
| Date Submitted by the Author: | 27-Jan-2021 |





| | |
|---|---|
| Complete List of Authors: | Thorgeirsdottir, Lilja; Sahlgrenska Academy, 1Department of Reproductive and Perinatal health, Institute of Health and Care Sciences<br>Andersson, Malin; Sahlgrenska Academy, Institute of clinical sciences, department of Anesthesiology and Intensive Care<br>Karlsson, Ove ; Sahlgrenska Academy, Department of Anesthesiology and Intensive Care, Institute of clinical sciences<br>Thörn, Sven-Egron; Institute of Clinical Sciences at Sahlgrenska Academy, University of Gothenburg, Department of Anaesthesiology and Intensive Care Medicine<br>Oras, Jonatan; Sahlgrenska Academy, 3Department of Anesthesiology and Intensive Care, Institute of clinical sciences<br>Sengpiel, Verena; University of Gothenburg Sahlgrenska Academy, Department of obstetrics and gynecology; Sahlgrenska Universitetssjukhuset Östra sjukhuset, Department of obstetrics and gynecology<br>Svanvik, Teresia; University of Gothenburg Sahlgrenska Academy, Department of obstetrics and gynecology<br>Elden, Helen; Goteborgs Universitet, Health and Care Sciences<br>Linden, Karolina; Goteborgs universitet Institutionen for vardvetenskap och halsa,<br>Junus, Katja; Uppsala University, Department of women's and children's health<br>Lager, Susanne; Uppsala University, Women's and Children's Health; Uppsala University, Centre for Reproductive Biology in Uppsala (CRU)<br>Enskär, Ida; Uppsala University, Department of women's and children's health<br>van Veen, Teelkien; University Medical Centre Groningen, Department of obstetrics and gynecology<br>Wikström, Johan; Department of Surgical Sciences, Radiology Unit, Department of Surgical Sciences, Radiology Unit<br>Björkman-Burtscher, Isabella; Göteborgs universitet Institutionen för kliniska vetenskaper, Radiology<br>Stigsdotter Neely, Anna; Karlstad University, Department of Social and Psychological Studies<br>Wikström, Anna-Karin; Karolinska institutet, Department of Medicine Unit of clinical epid; Uppsala University, Womens and Childrens Health<br>Bergman, Lina ; Sahlgrenska Academy, Institute of clinical sciences, department of obstetrics and gynecology; Uppsala University, Department of women's and children's health |

| | |
|---|---|
| Keywords: | Maternal medicine < OBSTETRICS, RADIOLOGY & IMAGING, ULTRASONOGRAPHY, NEUROLOGY, QUALITATIVE RESEARCH |
| | |

SCHOLARONE™
Manuscripts

*I, the Submitting Author has the right to grant and does grant on behalf of all authors of the Work (as defined in the below author licence), an exclusive licence and/or a non-exclusive licence for contributions from authors who are: i) UK Crown employees; ii) where BMJ has agreed a CC-BY licence shall apply, and/or iii) in accordance with the terms applicable for US Federal Government officers or employees acting as part of their official duties; on a worldwide, perpetual, irrevocable, royalty-free basis to BMJ Publishing Group Ltd ("BMJ") its licensees and where the relevant Journal is co-owned by BMJ to the co-owners of the Journal, to publish the Work in this journal and any other BMJ products and to exploit all rights, as set out in our licence.*

*The Submitting Author accepts and understands that any supply made under these terms is made by BMJ to the Submitting Author unless you are acting as an employee on behalf of your employer or a postgraduate student of an affiliated institution which is paying any applicable article publishing charge ("APC") for Open Access articles. Where the Submitting Author wishes to make the Work available on an Open Access basis (and intends to pay the relevant APC), the terms of reuse of such Open Access shall be governed by a Creative Commons licence – details of these licences and which Creative Commons licence will apply to this Work are set out in our licence referred to above.*

*Other than as permitted in any relevant BMJ Author's Self Archiving Policies, I confirm this Work has not been accepted for publication elsewhere, is not being considered for publication elsewhere and does not duplicate material already published. I confirm all authors consent to publication of this Work and authorise the granting of this licence.*

For peer review only

# Establishment of a multi-center preeclampsia database and biobank in Sweden – GO PROVE and UP MOST, a prospective cohort study

Lilja Thorgeirsdottir[1,2], Malin Andersson[3], Ove Karlsson[3], Sven-Egron Thörn[3], Jonatan Oras[3], Verena Sengpiel[2,4], Teresia Svanvik[2,4], Helen Elden[1,2], Karolina Linden[1], Katja Junus[5], Susanne Lager[5], Ida Enskär[5], Teelkien van Veen[6], Johan Wikström[7], Isabella M. Björkman-Burtscher[8], Anna Stigsdotter Neely[8,9], Anna Karin Wikström[5], Lina Bergman[2,4,11]

[1]Department of Reproductive and Perinatal health, Institute of Health and Care Sciences, Sahlgrenska Academy, University of Gothenburg, Gothenburg, Sweden

[2]Department of Obstetrics and Gynecology, Sahlgrenska University Hospital, Gothenburg, Sweden

[3]Department of Anesthesiology and Intensive Care, Institute of clinical sciences, Sahlgrenska Academy, University of Gothenburg, Gothenburg, Sweden

[4]Department of Obstetrics and Gynecology, Institute of clinical sciences, Sahlgrenska Academy, University of Gothenburg, Gothenburg, Sweden

[5]Department of Women's and Children's Health, Uppsala University, Uppsala, Sweden

[6]Department of Obstetrics and Gynecology, University Medical Center Groningen, Groningen, the Netherlands

[7]Department of Surgical Sciences, Radiology, Uppsala University

[8]Department of Radiology, Institute of Clinical Sciences, Sahlgrenska Academy, University of Gothenburg and Sahlgrenska University Hospital, Gothenburg, Sweden

[8]Department of Social and Psychological Studies, Karlstad University, Sweden

[9]Engineering Psychology Luleå University of Technology

[11]Department of Obstetrics and Gynecology, Stellenbosch University, Cape Town, South Africa

Corresponding author

Lina Bergman

Department of Obstetrics and Gynecology

PO Kvinnokliniken SU Östra

SE 416 85 Göteborg

Sweden

For peer review only

Telephone number 0046707920780

Email: lina.bergman.2@gu.se

Word count: 3561

For peer review only



For peer review only - http://bmjopen.bmj.com/site/about/guidelines.xhtml

## ABSTRACT

### Introduction

Preeclampsia, a multi-system disorder in pregnancy, is one of the most common causes of maternal morbidity and mortality worldwide. However, we lack methods for objective assessment of organ function in preeclampsia and predictors of organ impairment during and after preeclampsia. The women's and their partners' experiences of preeclampsia have not been studied in detail. To phenotype different subtypes of the disorder is of importance for prediction, prevention, surveillance, treatment and follow-up of preeclampsia.

The aim of this study is to set up a multi-center database and biobank for preeclampsia in order to contribute to a safer and more individualized treatment and care.

### Methods and analysis

This is a prospective multicenter cohort study. Pregnant women ≥18 years, diagnosed with preeclampsia presenting at Sahlgrenska University Hospital, Uppsala University Hospital and at Södra Älvsborgs Hospital, Sweden, as well as normotensive controls are eligible for participation. At inclusion and at one-year follow-up, the participants donate bio samples that are stored in a biobank and they are also asked to participate in various organ specific evaluations. In addition, questionnaires and interviews regarding the women's and partner's experiences are distributed at follow up.

### Ethics and dissemination

By creating a data- and biobank, we will provide the means to explore the disorder in a broader sense and allow clinical and laboratory discoveries that can be translated to clinical trials aiming at improved care of women with preeclampsia. Further, to evaluate experiences and the psychological impact of being affected by preeclampsia can improve the care of pregnant women and their partners. In case of incidental pathological findings during examinations performed, they will be handled in accordance with clinical routine. Data is


For peer review only

stored in a secure online database. Biobank samples are identified through the women's personal identification number and pseudonymised after identification in the biobank before analysis.

**Trial Registration**

Trial ID: ISRCTN13060768

Date registered: 27/07/2020

Link: https://www.isrctn.com/ISRCTN13060768

**Keywords**: (3-10 max) Biobank, brain, cerebral autoregulation, cognition, partner experience, RH-PAT, UCG, MRI

**Strengths and limitations of this study**

- This study is a national three center study, enabling inclusion of women from different socioeconomic backgrounds and by the size of the units, permits a large biobank and database of preeclampsia in a short time frame.
- The interdisciplinary team of obstetricians, midwives, anaesthesiologists, radiologists and cardiologists and collaboration between units from developed and developing countries contribute to a wider understanding of the disease from different aspects.
- The study uses pre-defined variables by an international consensus of predictors and outcomes in preeclampsia research – enabling merging of data with other international research groups.
- The hospital integrated biobanks ensure a high quality and standardized methods for the biological samples included in the study.



For peer review only - http://bmjopen.bmj.com/site/about/guidelines.xhtml

- Women with preeclampsia are delivered (as a cure) often before onset of complications and thus the endpoints will often consist of "preterm delivery" instead of true maternal organ complications.



For peer review only - http://bmjopen.bmj.com/site/about/guidelines.xhtml

## INTRODUCTION

Preeclampsia is one of the most serious complications of pregnancy affecting 3-8 % of pregnancies worldwide.[1,2] Preeclampsia leads to more than 60,000 maternal deaths annually and is also a common cause of maternal and neonatal morbidity.[2,3] Preeclampsia is a multi-organ disorder involving the brain, cardiovascular system, fetoplacental unit, hematological system, kidneys, liver, lungs and maternal vessels.[1] It may cause heart failure, pulmonary edema, renal failure, hemostatic disorders, cerebrovascular events, seizures (eclampsia), intrauterine growth restriction and intrauterine death. The pathophysiology of preeclampsia is not completely understood, but the hypothesis is based on placental dysfunction, in particular in early onset disease (diagnosis <34 gestational weeks). In late onset disease, maternal factors such as diabetes, cardiac disease, multi-fetal pregnancy and obesity are thought to play a more important role.[4] There is a paucity of data on how to evaluate threatening organ dysfunction in preeclampsia. At present, there are only a few tests available mainly regarding liver-, coagulation- and renal function.[5] Clinicians lack predictors or objective assessment of cardiovascular, endothelial, or neurological dysfunction in preeclampsia. An increased understanding of the underlying pathophysiology of organ complications and increased accuracy regarding prediction would allow individualized treatment. This includes magnesium sulphate for prevention of seizures, targeted blood pressure treatment for prevention of intracranial hemorrhage, customized administration of regional anesthesia, particularly in cases with disturbed coagulation and targeted treatment in case of cardiac dysfunction. By identifying biomarkers with high sensitivity and specificity for various threatening organ complications, end organ complications could be identified and managed in time and some cases of iatrogenic preterm labor could be avoided, potentially improving the short- and long-term outcomes for babies born to women with early onset preeclampsia.[6]



For peer review only - http://bmjopen.bmj.com/site/about/guidelines.xhtml

The capacity to recognize, exploit and phenotype different subtypes of preeclampsia is of importance for prediction, prevention, surveillance, treatment and follow-up of preeclampsia. This protocol adheres to the international consensus about standardized data and biological sample collections in preeclampsia research.[7] Adherence to such standardized protocols will facilitate the cooperation with other research teams and hopefully improve the understanding of the causes of preeclampsia and development of targeted treatment strategies.

The women's and their partners' experiences of preeclampsia during pregnancy and postpartum has not been studied in detail. Qualitative interview studies with women with preeclampsia have described preeclampsia as a fearful, powerless, painful and/or life-threatening event. They have also described inadequate clinical management and care and many women felt lucky to have survived.[8,9] By studying how women and their partners experience preeclampsia and the consequences regarding health-related quality of life, self-efficacy, anxiety, childbirth experiences and breastfeeding, we aim to improve personalized care and follow-up after a pregnancy complicated by preeclampsia.

For peer review only

## METHODS/DESIGN

This is a Swedish multicenter prospective cohort study including women diagnosed with preeclampsia and their partners and in addition normotensive controls. A flow chart of the study is presented in Figure 1. The study started to include women during the fall of 2019 and will continue to recruit until 2023.

### Aims

The overall aim of this study is to establish a database and biobank including two Swedish University preeclampsia cohorts, GO PROVE (Gothenburg Preeclampsia Obstetric adverse events) and UP MOST (Uppsala Pregnancy Complication Study) for preeclampsia research, to facilitate laboratory-based, translational and clinical research on preeclampsia and to



For peer review only - http://bmjopen.bmj.com/site/about/guidelines.xhtml

contribute to a safer and more patient centered care of women with preeclampsia in the acute and long-term perspective, focused on specific organ involvement. Thus, the following actions are included in the project:

- To establish a biobank and database with core predictors and outcomes for preeclampsia research in Sweden (GO PROVE and UP MOST)
- To examine alterations in cerebral structure and function through magnetic resonance (MR) investigation of the brain, doppler examination of cerebral blood flow, cerebral biomarkers, electroencephalograms (EEG) and neurocognitive assessment of mental processing speed, working memory, executive functions, and episodic memory in women with preeclampsia compared to normotensive controls at diagnosis and at one year postpartum (GO PROVE and UP MOST)
- To examine cardiac impairment and prediction of cardiac impairment through echocardiography and cardiac biomarkers in women with preeclampsia compared to normotensive controls at diagnosis and at one year postpartum (GO PROVE)
- To examine vascular dysfunction through measurement of peripheral resistance and blood-based endothelial biomarkers in women with preeclampsia compared to normotensive controls at diagnosis and at one year postpartum (GO PROVE)
- To examine coagulation profiles through blood-based biomarkers and tests such as thromboelastometry (ROTEM®) and impedance aggregometry (Multiplate®) in women with preeclampsia compared to normotensive controls (GO PROVE) To increase the knowledge and understanding of women's and their partners' experiences of preeclampsia during pregnancy, birth and postpartum through validated measurements and qualitative interviews (GO PROVE)

**Population**

For peer review only

Pregnant women ≥18 years of age with a diagnosis of preeclampsia after gestational week 20, presenting at Sahlgrenska University Hospital (10 500 deliveries/year), Uppsala University Hospital (4 200 deliveries/year) and Södra Älvsborgs Hospital (3 100 deliveries/year) are approached for inclusion. Both participating women and their partner are eligible for the qualitative interview study. Healthy women ≥18 years with normotensive pregnancies are included as controls.

Exclusion criteria: Women unable to understand oral and written information and unable to give written informed consent are not eligible as well as women with pre-existing hypertension, diabetes mellitus before or during pregnancy and pre-existing cardiovascular, renal or cerebral disease. MR contraindications for MR group includes claustrophobia.

**GO PROVE and UP MOST database**

The database is provided by MedSciNet (MedSciNet AB, Stockholm, Sweden) and includes exposure and outcome variables as proposed for preeclampsia research by the international collaborative group for preeclampsia research Co-Lab.[7] In addition, specific variables for this study have been included (Table 1 and Table 2). Biochemical tests when performed in routine health care are also collected, in addition to biochemical tests analyzed specifically for the study, and are presented in Table 3.

**GO PROVE and UP MOST Biobank**

GO PROVE and UP MOST use the Hospital Integrated Biobank (SIB, www.biobanksverige.se) that enables samples to be handled in a standardized manner through the hospitals' clinical laboratories, thus ensuring sample quality. Samples are sent to the local hospital's clinical chemistry laboratory or directly to the hospital biobank facility, where they are spun, aliquoted into 225 µl wells and frozen within four to six hours. Samples are only thawed directly prior to analysis. Each new project utilizing the biobank requires ethical approval, but no additional informed consent. At inclusion in the study, at delivery



For peer review only - http://bmjopen.bmj.com/site/about/guidelines.xhtml

and at one year follow up, maternal blood and urine are collected. At delivery, placental tissue, umbilical cord blood sample and cerebrospinal fluid are collected in addition to above. If the woman undergoes MRI brain, an extra blood sample is drawn. A detailed description of samples can be found in Table 4. By merging the participants in the biobank with the GO PROVE and UP MOST database, a data- and biobank is created that will also include results from organ-specific evaluation as described below.

**GO PROVE and UPMOST examinations**

For an overview of included examinations, please see Figure 1.

*Magnetic resonance (MR) examination of the brain, assessment of cerebral autoregulation and cognitive function scoring*

MR brain

Investigations will be performed using 3T MR scanners at inclusion and at one year postpartum for cases and controls. Imaging protocols contain the following sequences: 3D T1-weighted (w) MPRAGE sequence, 3D fluid attenuated inversion recovery (FLAIR), and 2D diffusion weighted imaging (DWI) with three directions and multiple b-values for morphological evaluation of e.g. edema and gross pathology as well as perfusion assessment using intravoxel incoherent motion (IVIM) technique,[10] 2D DWI with 32 directions to evaluate white matter microstructure,[11] arterial spin labeling (ASL) for perfusion assessment,[10,12] 3D time of flight (TOF) MR angiography for vessel evaluation, susceptibility weighted imaging (SWI/QSM or SWAN) for assessment of hemorrhage and resting state functional MR (rsfMR) for evaluation of neuronal networks.[13]

Neurocognitive assessment

To determine whether the participating women experience cognitive impairment, several instruments that measure, both subjective and objective cognition, provide a fine-grained picture of the participants' cognitive performance. Subjective cognitive function is assessed



For peer review only - http://bmjopen.bmj.com/site/about/guidelines.xhtml

by the Cognitive Failure Questionnaire (CFQ) measuring self-reported cognitive failures in everyday life.[14] The objective cognitive test battery consists of 11 tests covering a broad range of cognitive abilities. Verbal ability is assessed with a Swedish four-alternative multiple-choice synonym test (SRB 1) and serves as an estimate of premorbid cognitive ability.[15] Mental speed is measured by Digit Symbol Coding from Wechsler Adult Intelligence Scale (WAIS IV).[16] Working memory is assessed by the letter-number sequencing task and the three digit span tasks forward, backward and sequencing from WAIS-IV.[16] Executive functions are measured by three tasks from the Delis–Kaplan Executive Function System (D-KEFS) (a) Mental shifting/flexibility with the Trail Making Test; (b) Inhibition with the Stroop test; and (c) Verbal fluency with Letter and Category fluency test.[17] Several aspects of episodic memory performance are measured with free and delayed recall of nouns using the Buschke Selective Reminding procedure and prospective episodic memory with the Reminding task.[18,19] All cognitive tests have good psychometric properties. The tests are administered by study personnel, at inclusion at the hospital and again at one year postpartum.

Transcranial cerebral doppler

Measurement of the blood flow velocity in the middle cerebral artery (MCA) is performed with transcranial doppler technique and combined with continuous non-invasive measurement of the blood pressure using finger arterial volume clamping (calibrated with brachial blood pressure measurement) and carbon dioxide concentrations in exhaled air. The signal is obtained through the temporal window and systolic, mean and diastolic blood flow velocity and depth of measurement are registered. The measurements are performed by trained investigators and intra- and inter-observer variation is measured and kept below 15%. Cerebral autoregulation is determined from the response of the cerebral blood flow velocity to spontaneous fluctuations in mean blood pressure as described previously and reported as



For peer review only - http://bmjopen.bmj.com/site/about/guidelines.xhtml

Autoregulation Index.[20] The measurements will be compared between cases and normotensive controls and between inclusion and at one year postpartum.

*Echocardiography and endothelial dysfunction measurement*

<u>Echocardiography</u>

Women with preeclampsia and pregnant controls are examined with transthoracic echocardiography (TTE) for evaluation of systolic and diastolic function. Systolic dysfunction is defined as having an ejection fraction less than 50% or having regional hypokinesia. Diastolic dysfunction is defined as fulfilling at least 50% of the following; an average peak velocity of early diastolic transmitral flow / peak velocity of early diastolic mitral annular motion as determined by pulsed wave Doppler (E/e´) >14, septal e´ velocity < 7cm/s or lateral e´ velocity < 10cm/s, tricuspid regurgitation velocity > 2.8m/s or left atrial volume index >34ml/$m^2$[21]. Right ventricular function is assessed by eye-balling, tricuspid Annular Plane Systolic Excursion (TAPSE) and right ventricular peak velocity of systolic mitral annular motion as determined by pulsed wave Doppler (RV S´).

The examination is performed in proximity of inclusion and one year postpartum.

<u>Peripheral artery resistance measurements</u>

The status of the peripheral vessels can be measured with a variety of methods. In common, they are based on the interpretation of pulse pressure curve in the vessels. In this study we use the Reactive Hyperemia-Peripheral Arterial Tonometry (RH-PAT) method. The RH-PAT induces ischemia in one arm during 5 minutes. The pulse pressure wave in the peripheral artery, i.e. the finger, is measured before, during and after ischemia in both arms. The difference in reaction is recorded and reflects the vascular status peripherally.[22]

The Systemic Vascular Resistance index (SVRI) is calculated from the cardiac output and estimation of central venous pressure (CVP) measured through TTE indexed to body mass. SVRI reflects the afterload i.e. the workload that the heart has to handle at a normovolemic

For peer review only

state which in turn will be affected by the patient's blood pressure and peripheral vasoconstriction.[23]

The measurements of the heart function and the derived Systemic Vascular resistance retrieved from the TTE will then be compared with the measurement of peripheral vascular function as measured by the RH-PAT method and serum biomarkers clinically used to measure heart failure. The examination is performed at inclusion and again at one year postpartum.

*Hemostasis*

Hemostatic status can be assessed by laboratory analyses and by point-of-care devices as thromboelastometry (ROTEM®) and impedance aggregometry (Multiplate®). ROTEM® is an established viscoelastic method for hemostasis testing in whole blood and a modification of traditional thromboelastography (TEG®). ROTEM® investigates the interaction of coagulation factors, their inhibitors, anticoagulant drugs, blood cells (specifically platelets) during clotting and subsequent fibrinolysis.[24] Multiplate® measures platelet function through impedance aggregometry.[25] ROTEM® and Multiplate® are assessed at inclusion and again at delivery (GO-PROVE).

**Women's and partner's experiences of preeclampsia during pregnancy, childbirth and postnatal care**

*Questionnaires*

Effects of preeclampsia on women's self-reported health are measured with validated instruments sent to them by email, 8-12 weeks postpartum: Self-efficacy, the belief that one is capable of accomplishing a behavior or developing a competency to succeed in reaching a specific or more general goal, is measured by the General Self-Efficacy scale (GES).[26] Anxiety and depression are measured by Hospital anxiety and depression scale (HADS)[27] and Edinburgh postnatal depression scale (EPDS).[28] Health-related quality of life is determined

For peer review only

by the EQ-visual analog scale (EQ- VAS).[29] Childbirth experiences is measured by the Childbirth Experience Questionnaire (CEQ)[30] and levels of breastfeeding self-efficacy is assessed through the Breastfeeding Self-Efficacy Scale short form (BES).[31] Post-Traumatic Stress Symptoms is evaluated by the Post traumatic stress disorder (PTSD) Checklist, DSM-5 (PCL-5).[32]

*Interviews*

Experiences of preeclampsia during pregnancy, childbirth and postnatal care are described through qualitative interviews. Informants will be 15-20 women who have experienced preeclampsia with severe features during pregnancy and their partners.[33] Informants are selected to ensure a broad range of views and experiences of the phenomenon preeclampsia. Face-to-face interviews[34,35] are conducted 2 to 3 months after delivery. An open–ended question is used, "Please tell me of your experience of preeclampsia" or "please tell me of your experience as a partner to a woman with preeclampsia".

**Data processing, analysis and statistics**

Results from cerebral doppler, echocardiography, and RH-PAT estimations will be calculated by two independent interpreters blinded to groups and entered manually into the database. MR data will be analyzed in collaboration by neuroradiologists, blinded to groups. Blood samples will be thawed for analyses of cardiac-, renal-, neurological- and endothelial biomarkers, analyzed through standardized platforms when available and for the remaining through manual ELISA analyses in duplicates. Inter- and intra-assay coefficients of variation will be aimed at below 10%.

Demographics will be presented as medians or means as appropriate by distribution and by numbers and percentages as appropriate. Comparison between groups will be analyzed by student's t-test or Mann Whitney U-test with means or medians and confidence intervals or interquartile range, as appropriate according to distribution of the variables. Proportions will



For peer review only - http://bmjopen.bmj.com/site/about/guidelines.xhtml

307 be analyzed through Chi Square test. Correlations will be analyzed by Pearson's r or
308 Spearman's rho as appropriate by distribution of the variable. Regression analyses,
309 unadjusted and adjusted, will be performed to adjust for known confounding variables. Ten
310 cases per variable at the lowest will be considered appropriate to avoid overfitting of the
311 model. All statistical analyses will be performed in SPSS or R. Analyzes of cognitive
312 function will be performed through ANOVA AND MANOVA.
313 Data analysis of the interview data will be conducted by phenomenology with a lifeworld
314 approach[36] or with qualitative content analysis.[37] NVivo 10 software (qsrinternational.com/)
315 is used for grouping and organizing the text.

316 **Power calculations**

317 Uppsala and Sahlgrenska University hospitals have 4,200 and 10,500 deliveries yearly
318 respectively and Södra Älvsborgs Hospital 3,100 deliveries yearly. Preeclampsia affects 3%
319 of the population resulting in approximately 530 cases yearly at the centers and
320 approximately 410 cases after exclusions according to the study protocol. For logistical
321 reasons, all women cannot be included. Of eligible, we estimate that we will recruit around
322 20% for MR, transcranial doppler and cognition examinations (70/year) at Uppsala and
323 Sahlgrenska University hospitals and about 40/year at Sahlgrenska University hospital for
324 cardiac and endothelial function tests. Therefore, we estimate that inclusion will take two
325 years. Inclusion during two years at two centers will thus give around 140 MR investigations,
326 100 transcranial doppler and cognition examinations and 40 TTE. We will include 60
327 controls for each investigation. For blood based tests we will use the whole cohort. We want
328 to include more cases than controls since we want to be able to subtype cases based on
329 preeclampsia phenotypes. Some specific power calculations:

330 *Cerebral blood flow regulation and tissue perfusion:* in order to demonstrate a change of 3
331 ml / min x 100g in perfusion in the cortical sub-volume of the brain,[38] 44 cases of



For peer review only - http://bmjopen.bmj.com/site/about/guidelines.xhtml

preeclampsia and 44 normotensive controls with healthy pregnancies are required. In order to detect a difference in dynamic cerebral autoregulation index of 1.2,[20] a sample size of 32 women in each group is estimated to be required. In addition, differences in cerebral perfusion and dynamic cerebral autoregulation have been detected in the above sample sizes of 44 and 20 preeclampsia cases respectively with an equal number of normotensive controls.

*Cognition:* In a meta-analysis, a difference in the objective test "letter number sequencing task" was found when pooling results from 101 women with previous preeclampsia and 98 women with previous healthy pregnancies.[39]

**Patient and Public Involvement**

To ensure that the research is relevant, acceptable and feasible from the women's and partners' point of view patient representatives were invited to participate in the research process.[40] Focusing on women's and their partners' experiences of preeclampsia diagnosis and care at the same time will ensure that findings from this project will improve not only clinical outcome measures but also women's and their partners' experience.

## ETHICS AND DISSEMINATION

This is a cohort study with a control group with no intervention for the participants. Incidental pathological findings on examinations performed are and will be handled in accordance with clinical routine. Data is stored in a secure online database. Biobank samples are identified through the women's personal identification number and pseudonymised after identification in the biobank before analysis. Results from the study will be published in international peer-reviewed journals.

## DISCUSSION



For peer review only - http://bmjopen.bmj.com/site/about/guidelines.xhtml

There is a need for more individualized treatment of women with preeclampsia with respect to different organ manifestations of the disease. This in order to individualize treatment and care, such as an informed decision to deliver and to avoid short- and long-term complications for the mother and infant. Currently there are no reliable methods in clinical practice to evaluate organ dysfunction such as cardiovascular and cerebral effects of preeclampsia, although cerebral edema, intracranial bleeding and pulmonary edema secondary to heart failure are the most common causes of maternal mortality due to preeclampsia. There are emerging biomarkers and prediction models but none of them are used in routine care yet and they do not seem to be able to differentiate between different organ dysfunction.[41,42]

The interdisciplinary team of obstetricians, midwives, anesthesiologists, neuroradiologists and cardiologists enables us to link organ specialists in the field of preeclampsia research. This collaboration will enable important discoveries that have great potential to improve the care of these women. In addition, by employing a database created to harmonize data collection in preeclampsia research, biological samples and data can be shared with other research groups, enabling larger datasets for rare outcomes.[7]

Regarding cerebral dysfunction in preeclampsia, we will be able to further characterize imaging abnormalities, circulating cerebral biomarkers and cognitive function among women with preeclampsia and neurological impairment. Some women with preeclampsia show evidence of cerebral edema in the acute phase[43,44] and long-term follow-up of women with previous preeclampsia demonstrate an increased number of white matter lesions.[45] There is a paucity in data regarding the connection between acute lesions and long-term effects on the brain and their implications in the development of stroke, epilepsy, and dementia. Cerebral biomarkers have been reported to be increased in preeclampsia but the connection to objective findings on MR or cognitive function is not clear.[46,47] Hopefully, data from this study can enable an objective assessment of neurological function in preeclampsia as a tool

For peer review only



For peer review only - http://bmjopen.bmj.com/site/about/guidelines.xhtml

for targeting women at risk for adverse cerebral events. In addition, the long-term cerebral effects of preeclampsia are still an area that needs more research. There are reports of an increased risk of dementia, stroke and epilepsy after a pregnancy complicated by preeclampsia but the causality and importance of preeclampsia as an insult to the brain is not fully elucidated.[48-50] Cognitive impairment years after a pregnancy complicated by preeclampsia has been reported but it is not known whether this cognitive impairment exists already at diagnosis and how it changes over the first year postpartum after preeclampsia.[51,52] Findings from this study might increase the understanding of the underlying pathophysiological mechanisms of acute cerebral injury and long-term effects.

Regarding hemostasis, our results will hopefully provide guidance as to when women with preeclampsia can receive spinal anesthesia and further insight into the coagulation profile among women with severe preeclampsia.

For preeclampsia and cardiovascular function, an initial assessment of cardiovascular function with TTE and peripheral resistance measurement for assessment of endothelial dysfunction for all women in the study will aid in the risk-classification of women stratified on cardiovascular involvement.

Increased knowledge about how women as well as partners experience preeclampsia and the consequences could improve personalized care and follow-up after preeclampsia.

Preeclampsia is a disease only manifested in humans, hence there are no animal models of preeclampsia with the ability to exactly mimic the true preeclampsia state.[53] Therefore, it is imperative that critical laboratory and clinical observations are made on humans and human tissues. By creating a data- and biobank, we will be able to make important laboratory discoveries that can be translated to clinical trials that may improve the care of women.



For peer review only - http://bmjopen.bmj.com/site/about/guidelines.xhtml

**LIST OF ABBREVIATIONS**

BES Breastfeeding Self-Efficacy Scale short form

CEQ Childbirth Experience Questionnaire

CFQ Cognitive Failures Questionnaire

D-KEFS Delis-Kaplan Executive Function System

DSM 5 (PCL-5) Post Traumatic Stress Disorder Checklist

DWI Diffusion weighted images

EPDS Edinburgh Postnatal Depression Scale

EQ-VAS Euro Qol Visual Analogue Scale

FLAIR Fluid attenuated inversion recovery

GES General Self-Efficacy Scale

GO-PROVE Gothenburg Obstetric Adverse Events

HADS Hospital Anxiety and Depression Scale

MCA Middle cerebral artery

MRA 3D TOF Magnetic resonance angiography 3 dimensional time of flight

MR Magnetic Resonance

PROVE Preeclampsia Obstetric Adverse Events

PTSD Post traumatic stress disorder

RH-PAT reactive hyperemia-peripheral arterial tonometry

ROTEM Rotational tromboelastometry

SIB Hospital integrated biobank

SU Sahlgrenska University Hospital

TEG Thromboelastography

UP-PROVE Uppsala Pregnancy Complication Study

WAIS IV Wechsler Adult Intelligence Scale, fourth version

For peer review only



For peer review only - http://bmjopen.bmj.com/site/about/guidelines.xhtml

## DECLARATIONS

### Ethics approval and consent to participate

This study was approved by the regional ethical review board in Gothenburg on the 28$^{th}$ December 2018 (approval number 955-18) and by the Swedish Ethical Review Authority on the 27$^{th}$ of February 2019 (approval number 2019-00309).

All participating women are given oral and written information and sign an informed consent before participation in the study.

### Consent for publication

Not applicable.

### Availability of data and materials

The datasets used and/or analyzed during the current study are available from the corresponding author on reasonable request.

### Acknowledgment

We would like to thank Biobank Väst and Uppsala Biobank (a part of SIB) for supporting with collection of biological specimens.

For peer review only

## REFERENCES


1. Mol BWJ, Roberts CT, Thangaratinam S, Magee LA, de Groot CJM, Hofmeyr GJ. Pre-eclampsia. *Lancet.* 2016;387(10022):999-1011.
2. Duley L. The global impact of pre-eclampsia and eclampsia. *Semin Perinatol.* 2009;33(3):130-137.
3. Lain KY, Roberts JM. Contemporary concepts of the pathogenesis and management of preeclampsia. *JAMA.* 2002;287(24):3183-3186.
4. von Dadelszen P, Magee LA, Roberts JM. Subclassification of preeclampsia. *Hypertension in pregnancy : official journal of the International Society for the Study of Hypertension in Pregnancy.* 2003;22(2):143-148.
5. Brown MA, Magee LA, Kenny LC, et al. The hypertensive disorders of pregnancy: ISSHP classification, diagnosis & management recommendations for international practice. *Pregnancy Hypertens.* 2018.
6. Sibai BM. Preeclampsia as a cause of preterm and late preterm (near-term) births. *Seminars in Perinatology.* 2006;30(1):16-19.
7. Myatt L, Redman CW, Staff AC, et al. Strategy for standardization of preeclampsia research study design. *Hypertension.* 2014;63(6):1293-1301.
8. Furuta M, Sandall J, Bick D. Women's perceptions and experiences of severe maternal morbidity--a synthesis of qualitative studies using a meta-ethnographic approach. *Midwifery.* 2014;30(2):158-169.
9. Vaerland IE, Vevatne K, Brinchmann BS. An Integrative Review of Mothers' Experiences of Preeclampsia. *J Obstet Gynecol Neonatal Nurs.* 2016;45(3):300-307.
10. Le Bihan D, Breton E, Lallemand D, Aubin ML, Vignaud J, Laval-Jeantet M. Separation of diffusion and perfusion in intravoxel incoherent motion MR imaging. *Radiology.* 1988;168(2):497-505.


For peer review only



For peer review only - http://bmjopen.bmj.com/site/about/guidelines.xhtml

11. Pierpaoli C, Jezzard P, Basser PJ, Barnett A, Di Chiro G. Diffusion tensor MR imaging of the human brain. *Radiology.* 1996;201(3):637-648.

12. Williams DS, Detre JA, Leigh JS, Koretsky AP. Magnetic resonance imaging of perfusion using spin inversion of arterial water. *Proc Natl Acad Sci U S A.* 1992;89(1):212-216.

13. Biswal B, Yetkin FZ, Haughton VM, Hyde JS. Functional connectivity in the motor cortex of resting human brain using echo-planar MRI. *Magn Reson Med.* 1995;34(4):537-541.

14. Broadbent DE, Cooper PF, FitzGerald P, Parkes KR. The Cognitive Failures Questionnaire (CFQ) and its correlates. *Br J Clin Psychol.* 1982;21 (Pt 1):1-16.

15. Dureman I KL, Österberg E. *Manual till DS-batteriet [Manual for the DS-battery].* Stockholm: Psykologiförlaget; 1971.

16. Wechsler D. *WAIS-IV Administration and Scoring Manual.* San Antonio, TX: The Psychological Corporation; 2008.

17. Delis DC Kaplan E KJ. *Delis-Kaplan Executive Functioning System (D-KEFS).* San Antonio: The Psychological Corporation; 2001.

18. Buschke H. Selective reminding for analysis of memory and learning. *J Verb Learn Verb Behav.* 1973;12:543–550.

19. Mäntylä T, Nilsson LG. Remembering to remember in adulthood: A population-based study on aging and prospective memory. *Journal of Neuropsyhol Cogn.* 1997;4:81-92.

20. van Veen TR, Panerai RB, Haeri S, Griffioen AC, Zeeman GG, Belfort MA. Cerebral autoregulation in normal pregnancy and preeclampsia. *Obstet Gynecol.* 2013;122(5):1064-1069.

21. Nagueh SF SO, Appleton CP, Byrd BF 3rd, Dokainish H, Edvardsen T, Flachskampf FA, Gillebert TC, Klein AL, Lancellotti P, Marino P, Oh JK, Popescu BA, Waggoner

For peer review only



For peer review only - http://bmjopen.bmj.com/site/about/guidelines.xhtml

AD. Recommendations for the Evaluation of Left Ventricular Diastolic Function by Echocardiography: An Update from the American Society of Echocardiography and the European Association of Cardiovascular Imaging. *Journal Am Soc Echocardiogr.* 2016;29(4):277-314.

22. Meeme A BG, Mammen M, Namugowa A. Endothelial dysfunction and arterial stiffness in pre-eclampsia demonstrated by the EndoPAT method. *Cardiovascular Journal of Africa.* 2016;27:1-6.

23. Capeless EL CJ. Cardiovascular changes in early phase of pregnancy. *American Journal of Obstetrics and Gynecology.* 1989;161(6):1449-1453.

24. RJ L. Thrombelastography/thromboelastometry. *Clin Lab Haematol.* 2005;27(2):81-90.

25. Moenen FCJI VM, Nelemans PJ, van Rooy KJM, Vranken JRRA, Verhezen PWM, Wetzels RJH, Ten Cate H, Schouten HC, Beckers EAM, Henskens YMC. Screening for platelet function disorders with Multiplate and platelet function analyzer. *Platelets.* 2019;30(1):81-87.

26. Love J, Moore CD, Hensing G. Validation of the Swedish translation of the General Self-Efficacy scale. *Qual Life Res.* 2012;21(7):1249-1253.

27. Spinhoven PH OJ, Sloekers PPA, Kempen GIJM, Speckens AEM, Van Hemert AM. A validation study of the Hospital Anxiety and Depression Scale (HADS) in different groups of Dutch subjects. *Psychological Medicine.* 1997;27:363-370.

28. Cox JL HJ, Sagovsky R. Detection of Postnatal Depression Development of the 10-item Edinburgh Postnatal Depression Scale. *British Journal of Psychiatry.* 1987;150:782-786.

29. Group TE. EuroQol - a new facility for the measurement of health-related quality of life. *Health Policy.* 1990;16:199-208.

For peer review only



For peer review only - http://bmjopen.bmj.com/site/about/guidelines.xhtml

30. Dencker A, Taft C, Bergqvist L, Lilja H, Berg M. Childbirth experience questionnaire (CEQ): development and evaluation of a multidimensional instrument. *BMC Pregnancy Childbirth.* 2010;10:81.

31. Gerhardsson E, Nyqvist KH, Mattsson E, Volgsten H, Hildingsson I, Funkquist EL. The Swedish Version of the Breastfeeding Self-Efficacy Scale-Short Form: Reliability and Validity Assessment. *J Hum Lact.* 2014;30(3):340-345.

32. Blevins CA, Weathers FW, Davis MT, Witte TK, Domino JL. The Posttraumatic Stress Disorder Checklist for DSM-5 (PCL-5): Development and Initial Psychometric Evaluation. *J Trauma Stress.* 2015;28(6):489-498.

33. Patton M. *Qualitative Research& Evaluation Methods (3rd ed.).* Thousand Oaks,CA,: Sage Publications; 2002.

34. Graneheim U LB. Qualitative content analysis in nursing research: Concepts, procedures and measures to achieve trustworthiness. *Nurse Education Today.* 2004;24:105-112.

35. Polit D BC. *Nursing Research, Generating and Assessing Evidence fo Nursing Practice.* 8th ed: Wolters Kluwer/Lippincott Williams&Wilkins; 2008.

36. Dahlberg K DH, Nyström Pettersson M. *Reflective Lifeworld Research.* Studentlitteratur Lund; 2008.

37. Elo S KH. The qualitative content analysis process. *Journal of Advanced Nursing.* 2008;62(1):107-115.

38. Virhammar J, Laurell K, Ahlgren A, Larsson EM. Arterial Spin-Labeling Perfusion MR Imaging Demonstrates Regional CBF Decrease in Idiopathic Normal Pressure Hydrocephalus. *AJNR Am J Neuroradiol.* 2017;38(11):2081-2088.

For peer review only



For peer review only - http://bmjopen.bmj.com/site/about/guidelines.xhtml

39. Elharram M, Dayan N, Kaur A, Landry T, Pilote L. Long-Term Cognitive Impairment After Preeclampsia: A Systematic Review and Meta-analysis. *Obstet Gynecol.* 2018;132(2):355-364.

40. Sacristan JA, Aguaron A, Avendano-Sola C, et al. Patient involvement in clinical research: why, when, and how. *Patient Prefer Adherence.* 2016;10:631-640.

41. Thangaratinam S, Allotey J, Marlin N, et al. Prediction of complications in early-onset pre-eclampsia (PREP): development and external multinational validation of prognostic models. *BMC Med.* 2017;15(1):68.

42. Cerdeira AS, O'Sullivan J, Ohuma EO, et al. Randomized Interventional Study on Prediction of Preeclampsia/Eclampsia in Women With Suspected Preeclampsia. *Hypertension.* 2019:HYPERTENSIONAHA11912739.

43. Brewer J, Owens MY, Wallace K, et al. Posterior reversible encephalopathy syndrome in 46 of 47 patients with eclampsia. *Am J Obstet Gynecol.* 2013;208(6):468 e461-466.

44. Fisher N, Saraf S, Egbert N, Homel P, Stein EG, Minkoff H. Clinical Correlates of Posterior Reversible Encephalopathy Syndrome in Pregnancy. *J Clin Hypertens (Greenwich).* 2016;18(6):522-527.

45. Aukes AM, De Groot JC, Wiegman MJ, Aarnoudse JG, Sanwikarja GS, Zeeman GG. Long-term cerebral imaging after pre-eclampsia. *BJOG.* 2012;119(9):1117-1122.

46. Bergman L, Zetterberg H, Kaihola H, Hagberg H, Blennow K, Akerud H. Blood-based cerebral biomarkers in preeclampsia: Plasma concentrations of NfL, tau, S100B and NSE during pregnancy in women who later develop preeclampsia - A nested case control study. *PLoS One.* 2018;13(5):e0196025.

47. Evers KS, Atkinson A, Barro C, et al. Neurofilament as Neuronal Injury Blood Marker in Preeclampsia. *Hypertension.* 2018;71(6):1178-1184.

For peer review only



For peer review only - http://bmjopen.bmj.com/site/about/guidelines.xhtml

48. Basit S, Wohlfahrt J, Boyd HA. Pre-eclampsia and risk of dementia later in life: nationwide cohort study. *BMJ.* 2018;363:k4109.

49. Miller EC. Preeclampsia and Cerebrovascular Disease. *Hypertension.* 2019;74(1):5-13.

50. Nerenberg KA, Park AL, Vigod SN, et al. Long-term Risk of a Seizure Disorder After Eclampsia. *Obstet Gynecol.* 2017;130(6):1327-1333.

51. Aukes AM, Wessel I, Dubois AM, Aarnoudse JG, Zeeman GG. Self-reported cognitive functioning in formerly eclamptic women. *Am J Obstet Gynecol.* 2007;197(4):365 e361-366.

52. Postma IR, Groen H, Easterling TR, et al. The brain study: Cognition, quality of life and social functioning following preeclampsia; An observational study. *Pregnancy Hypertens.* 2013;3(4):227-234.

53. Sunderland N, Hennessy A, Makris A. Animal models of pre-eclampsia. *Am J Reprod Immunol.* 2011;65(6):533-541.

For peer review only



For peer review only - http://bmjopen.bmj.com/site/about/guidelines.xhtml

**Figure legends**

**Figure 1.**

Flow chart of the study. Investigations and biological samples from inclusion to one year postpartum. CSF; Cerebrospinal fluid, MRI; Magnetic resonance imaging

For peer review only

**Authors’ contributions**

The group of authors set up the study. LB and LT wrote the original draft. All authors provided intellectual input to the manuscript and approved the final version.

**Funding statement**

This study is supported by the Swedish Brain Fund, Märta Lundqvist’s foundation, Jane and Dan Olsson’s foundation, the Foundation of the Health and Medical care committee of the Region of Västra Götaland, Sweden (VGFOUREG851391), (VGFOUREG930918), Gothenburg Society of Medicine (GLS934555 and GLS878481), Sahlgrenska University Hospital, ALF Västra Götalands Region (ALFGBG-925851), Swedish Research Council (2020-01640), Swedish Society for Medical Research and Sahlgrenska University Hospital SU (2018-03591).

**Competing interest statement**

The authors declare that they have no competing interests.

For peer review only

**Table 1**. Variables included in the study.

| Variables from the woman's medical chart | Variables from the study inclusion visit, gathered from the patients´ medical charts | Variables from the study inclusion visit when diagnosed with preeclampsia, registered by patients in the enrolment module | Variables from the Swedish Pregnancy Register after delivery |
|---|---|---|---|
| Marital status (n)<br>Current living situation (n)<br>Country of birth (n)<br>Total years of education (c)<br>Profession (n)<br>Maternal occupation in the beginning of pregnancy (b)<br>Height (c)<br>Intra uterine growth restriction (b)<br>Preeclampsia (b)<br><br>**Obstetric history**<br>Number of pregnancies terminated before 21+6 weeks gestation (d)<br>Previous pregnancies, terminated before 21+6 weeks gestation, causes (n)<br>Gestational age (d)<br>Gestational hypertension (b)<br>Preeclampsia (b)<br>Eclampsia (b)<br>HELLP (b)<br>Gestational diabetes treated with Insulin or oral agents (b)<br>Treatment details (Free text)<br><br>Number of pregnancies beyond 21+6 weeks gestation (d)<br>Information on previous pregnancies, with delivery beyond 21+6 weeks gestation, outcome details:<br>Gestational age (d)<br>Gestational hypertension (b)<br>Preeclampsia (b)<br>Eclampsia (b)<br>HELLP (b) | **Study assessment at inclusion**<br>Date (c)<br>Location (n)<br>1st BP (c)<br>2nd BP (c)<br>Pulse (c)<br>Weight (c)<br>Proteinuria (dipstick) (o)<br>Urinary protein/creatinine ratio (c)<br>24 hour protein (c)<br>Gestation at PE/Intra uterine growth restriction (IUGR) diagnosis (c)<br><br>**Investigations at inclusion**<br>Samples taken, see table 3<br><br>**Urine sample**<br>See Table 3<br><br>**Gestational diabetes (GDM) screening, diagnosis treatment**<br>Date screened (c)<br>Date of GDM diagnosis (c)<br>Gestation GDM diagnosed (c)<br>Date of glucose tolerance test (d)<br><br>**Ultrasound and doppler scan after preeclampsia diagnosis**<br>Date of scan (d)<br>Fetal sex (n)<br>Gestational age when scanned (c)<br>Fetal abnormality (n)<br>Crown rump length (c)<br>Nuchal translucency (c)<br>Biparietal diameter (BPD (c) | Yearly income (o)<br>First pregnancy with the baby's biological father (b)<br>Months of sexual relationship (pre-pregnancy) (c)<br>Contraception used in this relationship (n)<br>If contraception has been used, which mostly in the last year (n)<br>Medications before pregnancy (n)<br>First degree relative/s with hypertensive disease in pregnancy (b)<br>First degree relative/s with hypertension, Ischaemic Heart Disease, Stroke or any form of Diabetes (b)<br>Pre-pregnancy weight (c)<br><br>**Partners Family History**<br>Children with other partners (b)<br>Pregnancy outcome with other partner (n)<br>First degree relative with preeclampsia or Eclampsia (b)<br>First degree relative/s with Hypertension (b), Ischaemic Heart Disease (b), Stroke (b) or any form of Diabetes (b)<br><br>**Partner's Data**<br>Age (c)<br>Weight (c)<br>Height (c)<br>Body mass index (c)<br>Partners medical condition (n)<br>Diabetes mellitus (b)<br>Hypertension (b)<br>Chronic Heart Disease (b)<br>Other (Free text) | **Maternal**<br>*Delivery data*<br>Date of admission pre-delivery (c)<br>GA at admission pre-delivery (c)<br>Onset of delivery (n)<br>Maximum temperature in labour (c)<br><br>Foetal compromise pre-labour Caesarean section<br>Place and supervision of delivery (n)<br>Duration of rupture of membranes (c)<br><br>Duration of 1st stage (c)<br>Duration of 2nd stage (c)<br><br>Mode of delivery (n)<br>Indication for operative delivery (n)<br>Indication for delivery (n)<br><br>*Medication*<br>Magnesium sulphate (n)<br>Steroids (n)<br>Antihypertensive treatment in labour (n)<br>Medication during labour and delivery only (Free text)<br>Calcium antagonist (b)<br><br>*Placenta*<br>Recommended standards of placental handling used (b)<br>Placental weight (c)<br>Cord insertion relative to placental margin (n)<br>Number of vessels in cord (b)<br>Pathology report (b)<br>Photograph against a scale bar (b) |



For peer review only - http://bmjopen.bmj.com/site/about/guidelines.xhtml

| | | | |
|---|---|---|---|
| Gestational diabetes treated with Insulin or oral | Head circumference (HC) (c) | | |
| agents (b) | Abdominal diameter (AD (c) | **Vitamin and mineral supplementation** | **Newborn data** |
| Treatment details (n) | Femur length (C) | **before /and during pregnancy** | Liveborn (b) |
| | Sub-chorionic haematoma (b) | Folate (c) | Date of birth (c) |
| **History of infertility** (b) | 2nd sac (b) | Multivitamin (o) | Time of birth (c) |
| Fertility treatment for this conception (b) | Risk score of Trisomy 21(d) | Vitamin D (o) | Gestational age at delivery (c) |
| Stimulation for ovulation for this conception (b) | Liquor volume (o) | Iron (o) | Sex (b) |
| Artificial insemination for this conception (n) | Placental Location (n) | Calcium (o) | Apgar score (d) |
| In Vitro fertilization for this conception (n) | Clinical suspicion for fetal growth restriction (b) | | Intubation at birth (n) |
| Intra Cytoplasmic sperm injection for this | Documented fetal growth restriction (b) | | Cord arterial pH (c) |
| conception (n) | Clinical indication for Macrosomia (b) | **Infection in pregnancy to record** (b) | Cord arterial Base deficit (c) |
| Embryo recipient for this conception (b) | Amniotic fluid index (c) | Type of infection (n) | Cord venous pH (c) |
| | Single deepest pocket (c) | Urinary tract infection (lower) (b) | Cord venous Base deficit (c) |
| **Medical conditions diagnosed before pregnancy** | | Pyelonephritis (b) | Birthweight (c) |
| Polycystic ovary syndrome (b) | *Umbilical dopplers* (c) | Human immunodeficiency virus (b) | Birthweight centile (c) |
| Medical conditions diagnosed before pregnancy (n) | Gestational age (c) | Active Tuberculosis (b) | Customised birthweight centile (c) |
| Hypertension (b) | Umbilical PI (c) | Zika virus (b) | Length (c) |
| Renal disease (b) | | Schistosomiasis (b) | Head circumference (c) |
| Anaemia (b) | *Uterine dopplers* | Active or chronic hepatitis (b) | TSH (c) |
| Diabetes type I (b) | L Ut Pulsatility Index (c) | Active or recently treated sexually | T3 total (c) |
| Diabetes type II (b) | R Ut Pulsatility Index (c) | transmitted infection (b) | T4 total (c) |
| Lupus disease (b) | Mean Ut PI (c) | Other infection in pregnancy (b) | T3 free (c) |
| Inflammatory bowel disease (b) | L Notch (c) | | T4 free (c) |
| Coeliac disease (b) | R Notch (c) | Diagnosis (n) | ICD code(s) for measures and diagnosis (n/free |
| Rheumatoid arthritis (b) | Other Ultrasound comments (Free text) | Treatment (n) | text) |
| Epilepsy (b) | Fetal Outcome (n) | | |
| Thromboembolism (b) | | **Symptoms at inclusion see Table X** | *Neonatal Outcome at discharge* |
| History of depression (b) | **Medication use at inclusion** | | Uncomplicated neonatal outcome (b) |
| Antiphospholipid syndrome (b) | Any medication at inclusion(b) | **At time of MRI brain and Cognitive tests** | Neonatal outcome (n) |
| HIV (b) | Low dose aspirin (b) | Blood pressure (c) | Date of discharge home (c) |
| Sickle cell (b) | Other antiplatelet medication (b) | Pulse (c) | Days in hospital from birth to discharge home |
| Malaria requiring treatment in the last year (b) | Low molecular weight heparin (n) | Oxygen saturation (d) | (d) |
| Asthma (b) | Diuretics (n) | | Admission to neonatal unit (c) |
| Oral steroids for asthma (b) | Labetalol (n) | Current treatment | Final discharge date from neonatal unit or date |
| Thyroid disease (n) | Beta blocking agent (n) | -Magnesium sulphate IVI (b) | of death in neonatal unit (c) |
| Migraine (b) | Calcium channel antagonist (n) | -Labetolol IVI (b) | Days in neonatal unit (d) |
| Irritable colon (b) | Antibiotic (n) | -Hydralazine IVI (b) | |
| Anxiety disorder (b) | Anticoagulants (n) | -Calcium antagonist short acting oral (b) | **Perinatal outcome** |
| Other medical conditions diagnosed before | Anticonvulsant-1 (not $MgSO_4$) (n) | -Labetolol oral (b) | Perinatal Survival (b) |
| pregnancy (free text) | Antidepressants (n) | -Calcium antagonist long acting oral (b) | Stillbirth (b) |
| | Antiglycemic agent –Insulin (b) | -ACE inhibitor oral (b) | Neonatal death (o) |
| Medications before pregnancy (n) | Metformin (b) | -AT-2 inhibitor oral (b) | |
| | Long-term corticosteroids (b) | -Diuretics IVI (b) | **Final Maternal and Perinatal Outcome** |

For peer review only



For peer review only - http://bmjopen.bmj.com/site/about/guidelines.xhtml

| | | | |
|---|---|---|---|
| **Last menstrual period** (LMP)<br>Certainty (n)<br>LMP date (c)<br>Gestational age by LMP/clinical examination (c)<br>Estimated due date by LMP (c)<br><br>***Estimated due date estimated by scan***<br><16 week scan date (c)<br>19-21 week scan date (c)<br>Final estimated due date (c)<br><br>**Clinical Readings at 1st Clinical Assessment**<br>Date of 1st clinical blood pressure (BP) (c)<br>Gestation of 1st BP (c)<br>1st clinical weight (c)<br>Proteinuria dipstick (o)<br>Urinary protein/creatinine ratio (c)<br>24 hour protein (c)<br><br>**Clinical Test**<br>(*Prenatal screening for chromosomal anomalies*)<br>Prenatal diagnosis (n)<br>Serum Screening for prenatal diagnosis (SSPD) (b)<br>Date of SSPD performed (c)<br>Gestation if SSPD performed (c)<br><br>**1st and 2nd Trimester Complications**<br>Hyperemesis (b)<br>Treatment in case of hyperemesis (free text)<br>Vaginal bleeding (b)<br>Gestational week if vaginal bleeding has occurred (c)<br>Duration of bleeding (d)<br>Amount of blood (o)<br><br>**Recreational Drug use**<br>Any recreational drug user (n)<br>Gestation ceased (c)<br>Tobacco (o)<br>Cigars (o) | Other immunosuppressant (n)<br>Thyroid supplement (b)<br>Antithyroid treatment for thyrotoxicosis (b)<br>Statins (n)<br>Other medication(n)<br>Hydralazine (b)<br>Magnesium sulphate (b)<br><br>Received steroids for fetal lung maturation before sampling (b)<br>Type of steroids (n)<br>Date started (c)<br>Gestational age started (c)<br>Date completed (c)<br>Gestational age completed (c)<br>Total dose (c)<br>Time between last dose and sampling (c) | -Diuretics oral (b)<br>-Other medications (b)<br><br>**Neurological symptoms at MRI brain, see table X**<br><br>**Echocardiocraphy and RH pat**<br>Date (c)<br>Blood pressure (c)<br>Pulse (c)<br>Oxygen saturation (d)<br>Oxygen administration (b)<br>Current treatment:<br>-Magnesium sulphate IVI (b)<br>-Labetolol IVI (b)<br>-Hydralazine IVI (b)<br>-Calcium antagonist short acting oral (b)<br>-Labetalol (b)<br>-Calcium antagonist long acting oral (b)<br>-ACE inhibitor oral (b)<br>-AT-2 inhibitor oral (b)<br>-Diuretics IVI (b)<br>-Diuretics oral (b)<br>-Other medications (b)<br>If *other*, state (FREE text)<br><br>**Variables from the visit at 1 year postpartum registered by the patient in the study module as well as study personnel**<br>Date (date)<br>Blood pressure (c)<br>Pulse (c)<br>Weight (c)<br>*Medication*<br>ACE inhibitor (b)<br>AT-2 inhibitor (b)<br>Diuretics (b)<br>Betablocker (b)<br>Aspirin (b)<br>Insulin (b)<br>Oral diabetes medication (b)<br>Lipid lowering medication (b) | Maternal outcome (n)<br>Maternal admission to intensive care unit (b)<br><br>**Pregnancy Summary & Complication**<br>Did any of these complications occur<br>-Normal pregnancy (b)<br>-Preeclampsia (b)<br>-Gestational Hypertension (b)<br>-Eclampsia or HELLP (b)<br>-Chronic hypertension (b)<br>-Placental abruption (b)<br>-Maternal death (b)<br>-Small for gestational age by population centile (b)<br>-Fetal growth retardation (b)<br>-Spontaneous preterm birth (b)<br>-PPROM with spontaneous preterm birth (b)<br>-Cervical insufficiency (b)<br>-Pregestational diabetes (b)<br>-Gestational diabetes (b)<br>-Cholestasis of pregnancy (b)<br>-Venous thromboembolism (b)<br>-Obese (BMI≥30) (b)<br>-Postpartum haemorrhage (PPH) (b)<br>-Other complications (b)<br>Maternal outcome comment (Free text)<br>-Perinatal outcome (n)<br>Fetal outcome comment (Free text)<br>Autopsy examination (n)<br>Major congenital abnormalities (n)<br>Chromosomal abnormality (n)<br>Congenital infection (b)<br>*ICD codes for diagnosis*<br>O14.0 (b)<br>O14.1A (b)<br>O14.1B (b)<br>O14.1X (b)<br>O14.9 (b)<br>O11.9 (b)<br>Other (b)<br>Procedure codes (FREE text) |

For peer review only



For peer review only - http://bmjopen.bmj.com/site/about/guidelines.xhtml

| | | | |
|---|---|---|---|
| Chewing tobacco (o)<br>Snuff (o)<br>E-cigarettes (o)<br>Nicotine replacement (o)<br>Alcohol (o)<br><br>*Other drugs in pregnancy*<br>Marijuana (b)<br>Cocaine/Crack (b)<br>Amphetamines (b)<br>Substance P/Crystal Meth (b)<br>XTC (b)<br>Opiates (b)<br>Hallucinogens (b)<br>Other (n)<br><br>Audit number 3 months prior to pregnancy (d)<br><br>**Ultrasound**<br>Date of scan (c)<br>Fetal sex (n)<br>Gestational age (c)<br>Fetal abnormality(n)<br>CRL (crown rump length) (c)<br>BPD (biparietal diameter) (c)<br>HC (head circumference) (c)<br>AD (abdominal diameter) (c)<br>FL (femur length) (c)<br>Sub-chorionic haematoma(b)<br>2nd sac (b)<br>Risk score of Trisomi 21(d)<br>Liquor Volume (o)<br>Placental Location (n)<br>Clinical suspicion for fetal growth restriction (FGR) (b)<br>Documented fetal growth restriction (b)<br>Clinical indication for Macrosomia (b)<br>Amniotic fluid index (AFI) (c)<br>Single deepest pocket (c)<br><br>*Umbilical dopplers*<br>Gestational age (c)<br>Umbilical RI (resistive index) (n) | | Levothyroxine (b)<br>Antidepressants (b)<br>Sleep medicine (b)<br><br>Did she breastfeed (b)<br>Period of breastfeeding (o)<br>Regained period (b)<br>Use of contraception (b)<br>-If yes, what contraception (free text)<br><br>After delivery, has the woman sought healthcare for;<br>-Migraine (b)<br>-High blood pressure (b)<br>-Visual disturbance (b)<br>-Diabetes (b)<br>-Thyroid function disorder (b)<br>-Allergies (b)<br>-Irritable colon (b)<br>-Alcohol or drug addiction (b)<br>-Depression (b)<br>-Anxiety (b)<br>-Pain-related disorders (b)<br><br>Cognitive test performed (b)<br>-STAI, PANAS, PSQI performed (b)<br>-EPDS, HADS performed (b)<br>-EQ-VAS, CEC, BSES, GES performed (b)<br>-Bioimpedance been performed (b)<br><br>**Neurological symptoms at 1 year postpartum, see Table X** | **Other severe complication at any time before or after delivery**<br>-None (b)<br>-Unknown (b)<br>-DIC (b)<br>-Acute renal insufficiency (b)<br>-No urine catheter output for more than 60 min (b)<br>-Haemolysis (b)<br>-CVA (b)<br>-Epigastric pain (b)<br>-Pulmonary Oedema (b)<br>-Hepatic rupture (b)<br>-Severe sepsis (b)<br>-Maternal death (b)<br>-Foetal death (b)<br>-Thrombocytopenia (b)<br>-Abnormal LFT (due to PE) (b)<br>-Coma (b)<br>-Jaundice (b)<br>-Respiratory failure (b)<br>-Cardiac failure (b)<br>-Venous Thromboembolism (b)<br>-Other complications (b)<br>-If other, specify (FREE text)<br><br>**Summary of antenatal and postnatal lab results**<br>-Blood group (n)<br>-Rhesus factor (b)<br>Table Y<br><br>**End report-status**<br>Final pregnancy outcome (n)<br><br>If pregnancy ended $\leq 19^{+6}$, date (c)<br>Gestational age at pregnancy end (c)<br><br>**MRI brain 1 year postpartum**<br>Blood pressure (c)<br>Pulse (c)<br>Plasma sample taken (b) |

For peer review only

| | | | |
|---|---|---|---|
| Umbilical PI (pulsatility index) (n)<br>Umbilical vein (n)<br><br>*Uterine dopplers*<br>L Ut PI (n)<br>R Ut PI (n)<br>Mean Ut PI (n)<br>L notch (n)<br>R notch (n)<br>Foetal outcome (n) | | | |

610

For peer review only

**Table 2.** Neurological Symptoms at inclusion, at MRI brain and at follow up one year postpartum.

| |
|---|
| Edema (n) |
| Visual disturbances (b) |
| *No visual disturbances* (b) |
| *Blurred vision* (b) |
| *Double vision* (b) |
| *Temporary loss of vision* (b) |
| *Sensitivity to light* (b) |
| *Partial loss of vision or blind spots in normal field of vision* (b) |
| *Flickering lights* (b) |
| *Other* (n) |
| |
| Time of onset for visual disturbances (n) |
| *N/A (no visual disturbance)* (b) |
| |
| Headache (o) |
| Headache onset (o) |
| Epigastric/abdominal pain (o) |
| Tightness in chest (b) |
| Shortness of breath (b) |
| Focal neurological deficits (n) |
| |
| Tendon reflexes (n) |
| Nausea (b) |
| Vomit (b) |
| Form of confusion (b) |
| Twitching or jerking in arms and/or legs (b) |
| Has the woman's family/friends experienced the patient as not responding or staring blankly into space (b) |
| Difficulty concentrating (b) |
| Speech affected (b) |
| Hearing affected (b) |
| Mood changes (n) |
| Other mood changes than listed (Free text) |
| |
| *Before being diagnosed with PE/MRI scan/visit at 1 year postpartum did the woman* |
| -feel anxious (b) |
| -feel as if the end of the world was coming (b) |
| -experience severe dizziness (b) |
| -experience any weakness or paralysis (b) |
| -experience jitters/nervousness/nervous shaking (b) |
| -experienced any other symptoms not listed (b) |
| List other symptoms (FREE text) |

For peer review only

**Table 3.** Biochemical analyses specific for the study and in clinical routine when available.

| Prenatal screening | At inclusion | At delivery and Summary of analyses at discharge* |
|---|---|---|
| **If taken in clinical routine**<br>human chorionic gonadotropin) (c)<br>Oestriol (c)<br>Alpha-fetoprotein (c)<br>Inhibin A (c)<br>Pregnancy-associated plasma protein A (c)<br>Cell free fetal DNA (c) | **If taken in clinical routine**<br>Haemoglobin (c)<br>Haematocrit (c)<br>WCC (white cell count) (c)<br>Platelets (c)<br>Creatinine (c)<br>AST (aspartate transaminase) (c)<br>ALT (alanine transaminase) (c)<br>Total bilirubin (c)<br>Albumin (c)<br>LDH (lactate dehydrogenase) (c)<br>Haptoglobin (c)<br>APTT (activated partial thromboplastin time) (c)<br>PR (prothrombin ratio) (c)<br>INR (International normalize ratio) (c)<br>CRP (C reactive protein) (c)<br>Oxygen saturation (d)<br>Total leukocyte count (c)<br>Urinary prot/creat ratio (c)<br>Fibrinogen (c)<br>Antitrombin (c)<br><br>**Specific for the study**<br>ROTEM (b)<br>Multiplate (b)<br>NT-pro BNP (c)<br>Troponin (c) | **At delivery specific for the study**<br>ROTEM (b)<br>Multiplate (b)<br>Platelets (c)<br>Troponin (c)<br>NT pro BNP (c)<br><br>**Summary of analyses at discharge if taken in clinical routine**<br>Haemoglobin (g/L) (c)<br>Platelet count (x10^9/L) (c)<br>Urinary protein/creatinine ratio (mg/g) (c)<br>Creatinine (mmol/L) (c)<br>Aspartate transaminase (IU/L) (c)<br>Alanine transaminase (IU/L) (c)<br>GGT (IU/L) (c)<br>Total bilirubin (umol/L) (c)<br>Albumin (g/L) (c)<br>LDH(IU/L) (c)<br>Haptoglobin (mg/dL) (c)<br>APTT (sec) (c)<br>INR (c)<br>D-dimer (ug/L) (c)<br>CRP (mg/L) (c) |

**Lowest antepartum in hospital/Highest antepartum in hospital/Lowest value postpartum/Highest value postpartum*

For peer review only



For peer review only - http://bmjopen.bmj.com/site/about/guidelines.xhtml

**Table 4.** Biological sample collection

| At inclusion | At delivery | At MRI brain | At one year postpartum |
|---|---|---|---|
| Serum<br>EDTA plasma<br>Li/Heparin plasma<br>EDTA whole blood<br>Urine | Serum<br>EDTA plasma<br>Li/Heparin plasma<br>EDTA plasma umbilical cord<br>Cerebrospinal fluid<br>Placental villous tissue frozen<br>Placental villous tissue RNA later<br>Basal plate, full thickness frozen<br>Basal plate, full thickness RNA later | Serum<br>EDTA plasma | Serum<br>EDTA plasma |

For peer review only

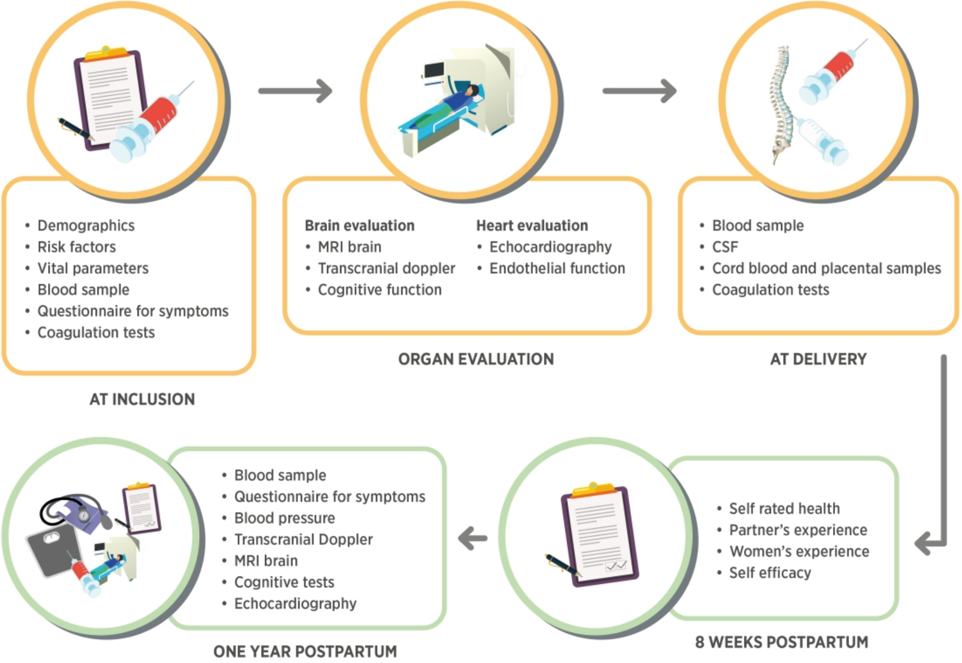


Flow chart of the study. Investigations and biological samples from inclusion to one year postpartum. CSF; Cerebrospinal fluid, MRI; Magnetic resonance imaging

297x209mm (300 x 300 DPI)

STROBE Statement—Checklist of items that should be included in reports of ***cohort studies***

| | Item No | Recommendation |
|---|---|---|
| **Title and abstract** | 1 | (*a*) Indicate the study's design with a commonly used term in the title or the abstract<br>Title page |
| | | (*b*) Provide in the abstract an informative and balanced summary of what was done and what was found<br>Page 3-4 |
| **Introduction** | | |
| Background/rationale | 2 | Explain the scientific background and rationale for the investigation being reported<br>Page 6-7 |
| Objectives | 3 | State specific objectives, including any prespecified hypotheses<br>Page 7-8 |
| **Methods** | | |
| Study design | 4 | Present key elements of study design early in the paper<br>Page 7 |
| Setting | 5 | Describe the setting, locations, and relevant dates, including periods of recruitment, exposure, follow-up, and data collection<br>Page 7, 9-14 |
| Participants | 6 | (*a*) Give the eligibility criteria, and the sources and methods of selection of participants. Describe methods of follow-up<br>Page 9 and Figure 1 |
| | | (*b*) For matched studies, give matching criteria and number of exposed and unexposed<br>N/A |
| Variables | 7 | Clearly define all outcomes, exposures, predictors, potential confounders, and effect modifiers. Give diagnostic criteria, if applicable<br>Page 8. The study is a biobank and database that will also address future research questions and outcomes. |
| Data sources/ measurement | 8* | For each variable of interest, give sources of data and details of methods of assessment (measurement). Describe comparability of assessment methods if there is more than one group<br>Page 9-14 |
| Bias | 9 | Describe any efforts to address potential sources of bias<br>Page 15 |
| Study size | 10 | Explain how the study size was arrived at<br>Page 15-16 |
| Quantitative variables | 11 | Explain how quantitative variables were handled in the analyses. If applicable, describe which groupings were chosen and why<br>N/A, study protocol |
| Statistical methods | 12 | (*a*) Describe all statistical methods, including those used to control for confounding<br>Page 15 |
| | | (*b*) Describe any methods used to examine subgroups and interactions<br>N/A |
| | | (*c*) Explain how missing data were addressed<br>N/A, study protocol |
| | | (*d*) If applicable, explain how loss to follow-up was addressed<br>N/A, study protocol |
| | | (*e*) Describe any sensitivity analyses |


For peer review only - http://bmjopen.bmj.com/site/about/guidelines.xhtml

| | | |
|---|---|---|
| | | N/A |
| **Results** | | |
| Participants | 13* | (a) Report numbers of individuals at each stage of study—eg numbers potentially eligible, examined for eligibility, confirmed eligible, included in the study, completing follow-up, and analysed<br>N/A, study protocol |
| | | (b) Give reasons for non-participation at each stage<br>N/A, study protocol |
| | | (c) Consider use of a flow diagram<br>N/A, study protocol |
| Descriptive data | 14* | (a) Give characteristics of study participants (eg demographic, clinical, social) and information on exposures and potential confounders<br>N/A, study protocol |
| | | (b) Indicate number of participants with missing data for each variable of interest<br>N/A, study protocol |
| | | (c) Summarise follow-up time (eg, average and total amount)<br>N/A, study protocol |
| Outcome data | 15* | Report numbers of outcome events or summary measures over time<br>N/A, study protocol |
| Main results | 16 | (*a*) Give unadjusted estimates and, if applicable, confounder-adjusted estimates and their precision (eg, 95% confidence interval). Make clear which confounders were adjusted for and why they were included<br>N/A, study protocol |
| | | (*b*) Report category boundaries when continuous variables were categorized<br>N/A, study protocol |
| | | (*c*) If relevant, consider translating estimates of relative risk into absolute risk for a meaningful time period<br>N/A, study protocol |
| Other analyses | 17 | Report other analyses done—eg analyses of subgroups and interactions, and sensitivity analyses<br>N/A, study protocol |
| **Discussion** | | |
| Key results | 18 | Summarise key results with reference to study objectives<br>N/A, study protocol |
| Limitations | 19 | Discuss limitations of the study, taking into account sources of potential bias or imprecision. Discuss both direction and magnitude of any potential bias<br>Page 16-18 |
| Interpretation | 20 | Give a cautious overall interpretation of results considering objectives, limitations, multiplicity of analyses, results from similar studies, and other relevant evidence<br>N/A, study protocol |
| Generalisability | 21 | Discuss the generalisability (external validity) of the study results<br>N/A, study protocol |
| **Other information** | | |
| Funding | 22 | Give the source of funding and the role of the funders for the present study and, if applicable, for the original study on which the present article is based<br>Page 28 |

*Give information separately for exposed and unexposed groups.



For peer review only - http://bmjopen.bmj.com/site/about/guidelines.xhtml

For peer review only

**Note:** An Explanation and Elaboration article discusses each checklist item and gives methodological background and published examples of transparent reporting. The STROBE checklist is best used in conjunction with this article (freely available on the Web sites of PLoS Medicine at http://www.plosmedicine.org/, Annals of Internal Medicine at http://www.annals.org/, and Epidemiology at http://www.epidem.com/). Information on the STROBE Initiative is available at http://www.strobe-statement.org.

For peer review only



For peer review only - http://bmjopen.bmj.com/site/about/guidelines.xhtml

BMJ Open

# Study protocol; Establishment of a multi-center preeclampsia database and biobank in Sweden – GO PROVE and UP MOST, a prospective cohort study



| | |
|---|---|
| Journal: | *BMJ Open* |
| Manuscript ID | bmjopen-2021-049559.R1 |
| Article Type: | Protocol |
| Date Submitted by the Author: | 28-Aug-2021 |





| | |
|---|---|
| Complete List of Authors: | Thorgeirsdottir, Lilja; Sahlgrenska Academy, 1Department of Reproductive and Perinatal health, Institute of Health and Care Sciences<br>Andersson, Malin; Sahlgrenska Academy, Institute of clinical sciences, department of Anesthesiology and Intensive Care<br>Karlsson, Ove ; Sahlgrenska Academy, Department of Anesthesiology and Intensive Care, Institute of clinical sciences<br>Thörn, Sven-Egron; Institute of Clinical Sciences at Sahlgrenska Academy, University of Gothenburg, Department of Anaesthesiology and Intensive Care Medicine<br>Oras, Jonatan; Sahlgrenska Academy, 3Department of Anesthesiology and Intensive Care, Institute of clinical sciences<br>Sengpiel, Verena; University of Gothenburg Sahlgrenska Academy, Department of obstetrics and gynecology; Sahlgrenska Universitetssjukhuset Östra sjukhuset, Department of obstetrics and gynecology<br>Svanvik, Teresia; University of Gothenburg Sahlgrenska Academy, Department of obstetrics and gynecology<br>Elden, Helen; Goteborgs Universitet, Health and Care Sciences<br>Linden, Karolina; Goteborgs universitet Institutionen for vardvetenskap och halsa,<br>Junus, Katja; Uppsala University, Department of women's and children's health<br>Lager, Susanne; Uppsala University, Women's and Children's Health; Uppsala University, Centre for Reproductive Biology in Uppsala (CRU)<br>Enskär, Ida; Uppsala University, Department of women's and children's health<br>van Veen, Teelkien; University Medical Centre Groningen, Department of obstetrics and gynecology<br>Wikström, Johan; Department of Surgical Sciences, Radiology Unit, Department of Surgical Sciences, Radiology Unit<br>Björkman-Burtscher, Isabella; Göteborgs universitet Institutionen för kliniska vetenskaper, Radiology<br>Stigsdotter Neely, Anna; Karlstad University, Department of Social and Psychological Studies<br>Wikström, Anna-Karin; Karolinska institutet, Department of Medicine Unit of clinical epid; Uppsala University, Womens and Childrens Health<br>Bergman, Lina ; Sahlgrenska Academy, Institute of clinical sciences, department of obstetrics and gynecology; Uppsala University, Department of women's and children's health |

| | |
|---|---|
| <b>Primary Subject Heading</b>: | Reproductive medicine |
| Secondary Subject Heading: | Obstetrics and gynaecology |
| Keywords: | Maternal medicine < OBSTETRICS, RADIOLOGY & IMAGING, ULTRASONOGRAPHY, NEUROLOGY, QUALITATIVE RESEARCH |
| | |


SCHOLARONE™
Manuscripts

*I, the Submitting Author has the right to grant and does grant on behalf of all authors of the Work (as defined in the below author licence), an exclusive licence and/or a non-exclusive licence for contributions from authors who are: i) UK Crown employees; ii) where BMJ has agreed a CC-BY licence shall apply, and/or iii) in accordance with the terms applicable for US Federal Government officers or employees acting as part of their official duties; on a worldwide, perpetual, irrevocable, royalty-free basis to BMJ Publishing Group Ltd ("BMJ") its licensees and where the relevant Journal is co-owned by BMJ to the co-owners of the Journal, to publish the Work in this journal and any other BMJ products and to exploit all rights, as set out in our licence.*

*The Submitting Author accepts and understands that any supply made under these terms is made by BMJ to the Submitting Author unless you are acting as an employee on behalf of your employer or a postgraduate student of an affiliated institution which is paying any applicable article publishing charge ("APC") for Open Access articles. Where the Submitting Author wishes to make the Work available on an Open Access basis (and intends to pay the relevant APC), the terms of reuse of such Open Access shall be governed by a Creative Commons licence – details of these licences and which Creative Commons licence will apply to this Work are set out in our licence referred to above.*

*Other than as permitted in any relevant BMJ Author's Self Archiving Policies, I confirm this Work has not been accepted for publication elsewhere, is not being considered for publication elsewhere and does not duplicate material already published. I confirm all authors consent to publication of this Work and authorise the granting of this licence.*

For peer review only

# Study protocol; Establishment of a multi-center preeclampsia database and biobank in Sweden – GO PROVE and UP MOST, a prospective cohort study

Lilja Thorgeirsdottir[1,2], Malin Andersson[3], Ove Karlsson[3], Sven-Egron Thörn[3], Jonatan Oras[3], Verena Sengpiel[2,4], Teresia Svanvik[2,4], Helen Elden[1,2], Karolina Linden[1], Katja Junus[5], Susanne Lager[5], Ida Enskär[5], Teelkien van Veen[6], Johan Wikström[7], Isabella M. Björkman-Burtscher[8], Anna Stigsdotter Neely[8,9], Anna Karin Wikström[5], Lina Bergman[2,4,11]

[1]Institute of Health and Care Sciences, Sahlgrenska Academy, University of Gothenburg, Gothenburg, Sweden

[2]Department of Obstetrics and Gynecology, Sahlgrenska University Hospital, Gothenburg, Sweden

[3]Department of Anesthesiology and Intensive Care, Institute of clinical sciences, Sahlgrenska Academy, University of Gothenburg, Gothenburg, Sweden

[4]Region Västra Götaland, Sahlgrenska University Hospital, Department of obstetrics and gynecology, Gothenburg, Sweden

Department of Obstetrics and Gynecology, Institute of clinical sciences, Sahlgrenska Academy, University of Gothenburg, Gothenburg, Sweden

[5]Department of Women's and Children's Health, Uppsala University, Uppsala, Sweden

[6]Department of Obstetrics and Gynecology, University Medical Center Groningen, Groningen, the Netherlands

[7]Department of Surgical Sciences, Radiology, Uppsala University

[8]Department of Radiology, Institute of Clinical Sciences, Sahlgrenska Academy, University of Gothenburg and Sahlgrenska University Hospital, Gothenburg, Sweden

[8]Department of Social and Psychological Studies, Karlstad University, Sweden

[9]Engineering Psychology Luleå University of Technology

[11]Department of Obstetrics and Gynecology, Stellenbosch University, Cape Town, South Africa

Corresponding author

Lina Bergman

Department of Obstetrics and Gynecology

PO Kvinnokliniken SU Östra

SE 416 85 Göteborg



For peer review only - http://bmjopen.bmj.com/site/about/guidelines.xhtml

Sweden

Telephone number 0046707920780

Email: lina.bergman.2@gu.se

Word count: 3561

For peer review only

## ABSTRACT

### Introduction

Preeclampsia, a multi-system disorder in pregnancy, is one of the most common causes of maternal morbidity and mortality worldwide. However, we lack methods for objective assessment of organ function in preeclampsia and predictors of organ impairment during and after preeclampsia. The women's and their partners' experiences of preeclampsia have not been studied in detail. To phenotype different subtypes of the disorder is of importance for prediction, prevention, surveillance, treatment and follow-up of preeclampsia.

The aim of this study is to set up a multi-center database and biobank for preeclampsia in order to contribute to a safer and more individualized treatment and care.

### Methods and analysis

This is a multicenter cohort study. Prospectively recruited pregnant women ≥18 years, diagnosed with preeclampsia presenting at Sahlgrenska University Hospital, Uppsala University Hospital and at Södra Älvsborgs Hospital, Sweden, as well as normotensive controls are eligible for participation. At inclusion and at one-year follow-up, the participants donate bio samples that are stored in a biobank and they are also asked to participate in various organ specific evaluations. In addition, questionnaires and interviews regarding the women's and partner's experiences are distributed at follow up.

### Ethics and dissemination

By creating a data- and biobank, we will provide the means to explore the disorder in a broader sense and allow clinical and laboratory discoveries that can be translated to clinical trials aiming at improved care of women with preeclampsia. Further, to evaluate experiences and the psychological impact of being affected by preeclampsia can improve the care of pregnant women and their partners. In case of incidental pathological findings during examinations performed, they will be handled in accordance with clinical routine. Data is


For peer review only



For peer review only - http://bmjopen.bmj.com/site/about/guidelines.xhtml

stored in a secure online database. Biobank samples are identified through the women's personal identification number and pseudonymised after identification in the biobank before analysis.

This study was approved by the regional ethical review board in Gothenburg on the 28th December 2018 (approval number 955-18) and by the Swedish Ethical Review Authority on the 27th of February 2019 (approval number 2019-00309).

Results from the study will be published in international peer-reviewed journals.

## Trial Registration

Trial ID: ISRCTN13060768

Date registered: 27/07/2020

Link: https://www.isrctn.com/ISRCTN13060768

**Keywords**: (3-10 max) Biobank, brain, cerebral autoregulation, cognition, partner experience, RH-PAT, UCG, MRI

## Strengths and limitations of this study

- This study is a national three center study, enabling inclusion of women from different socioeconomic backgrounds and by the size of the units, permits a large biobank and database of preeclampsia in a short time frame.
- The interdisciplinary team of obstetricians, midwives, anaesthesiologists, radiologists and cardiologists and collaboration between units from developed and developing countries contribute to a wider understanding of the disease from different aspects.
- The study uses pre-defined variables by an international consensus of predictors and outcomes in preeclampsia research – enabling merging of data with other international research groups.



For peer review only - http://bmjopen.bmj.com/site/about/guidelines.xhtml

- The hospital integrated biobanks ensure a high quality and standardized methods for the biological samples included in the study.
- Women with preeclampsia are delivered (as a cure) often before onset of complications and thus the endpoints will often consist of "preterm delivery" instead of true maternal organ complications.

For peer review only



For peer review only - http://bmjopen.bmj.com/site/about/guidelines.xhtml

## INTRODUCTION

Preeclampsia is one of the most serious complications of pregnancy affecting 3-8 % of pregnancies worldwide.[1,2] Preeclampsia leads to more than 60,000 maternal deaths annually and is also a common cause of maternal and neonatal morbidity.[2,3] Preeclampsia is a multi-organ disorder involving the brain, cardiovascular system, fetoplacental unit, hematological system, kidneys, liver, lungs and maternal vessels.[1] It may cause heart failure, pulmonary edema, renal failure, hemostatic disorders, cerebrovascular events, seizures (eclampsia), intrauterine growth restriction and intrauterine death. The pathophysiology of preeclampsia is not completely understood, but the hypothesis is based on placental dysfunction, in particular in early onset disease (diagnosis <34 gestational weeks). In late onset disease, maternal factors such as diabetes, cardiac disease, multi-fetal pregnancy and obesity are thought to play a more important role.[4] There is a paucity of data on how to evaluate threatening organ dysfunction in preeclampsia. At present, there are only a few tests available mainly regarding liver-, coagulation- and renal function.[5] Clinicians lack predictors or objective assessment of cardiovascular, endothelial, or neurological dysfunction in preeclampsia. An increased understanding of the underlying pathophysiology of organ complications and increased accuracy regarding prediction would allow individualized treatment. This includes magnesium sulphate for prevention of seizures, targeted blood pressure treatment for prevention of intracranial hemorrhage, customized administration of regional anesthesia, particularly in cases with disturbed coagulation and targeted treatment in case of cardiac dysfunction. By identifying biomarkers with high sensitivity and specificity for various threatening organ complications, end organ complications could be identified and managed in time and some cases of iatrogenic preterm labor could be avoided, potentially improving the short- and long-term outcomes for babies born to women with early onset preeclampsia.[6]

For peer review only



For peer review only - http://bmjopen.bmj.com/site/about/guidelines.xhtml

The capacity to recognize, exploit and phenotype different subtypes of preeclampsia is of importance for prediction, prevention, surveillance, treatment and follow-up of preeclampsia. This protocol adheres to the international consensus about standardized data and biological sample collections in preeclampsia research.[7] Adherence to such standardized protocols will facilitate the cooperation with other research teams and hopefully improve the understanding of the causes of preeclampsia and development of targeted treatment strategies.

The women's and their partners' experiences of preeclampsia during pregnancy and postpartum has not been studied in detail. Qualitative interview studies with women with preeclampsia have described preeclampsia as a fearful, powerless, painful and/or life-threatening event. They have also described inadequate clinical management and care and many women felt lucky to have survived.[8,9] By studying how women and their partners experience preeclampsia and the consequences regarding health-related quality of life, self-efficacy, anxiety, childbirth experiences and breastfeeding, we aim to improve personalized care and follow-up after a pregnancy complicated by preeclampsia.

## METHODS/DESIGN

This is a Swedish multicenter prospective cohort study prospectively including women diagnosed with preeclampsia and their partners and in addition normotensive controls. A flow chart of the study is presented in Figure 1. The study started to include women during the fall of 2019 and will continue to recruit until 2023.

### Aims

The overall aim of this study is to establish a database and biobank including two Swedish University preeclampsia cohorts, GO PROVE (Gothenburg Preeclampsia Obstetric adverse events) and UP MOST (Uppsala Pregnancy Complication Study) for preeclampsia research, to facilitate laboratory-based, translational and clinical research on preeclampsia and to



For peer review only - http://bmjopen.bmj.com/site/about/guidelines.xhtml

contribute to a safer and more patient centered care of women with preeclampsia in the acute and long-term perspective, focused on specific organ involvement. Thus, the following actions are included in the project:

- To establish a biobank and database with core predictors and outcomes for preeclampsia research in Sweden (GO PROVE and UP MOST)
- To examine alterations in cerebral structure and function through magnetic resonance (MR) investigation of the brain, doppler examination of cerebral blood flow, cerebral biomarkers, electroencephalograms (EEG) and neurocognitive assessment of mental processing speed, working memory, executive functions, and episodic memory in women with preeclampsia compared to normotensive controls at diagnosis and at one year postpartum (GO PROVE and UP MOST)
- To examine cardiac impairment and prediction of cardiac impairment through echocardiography and cardiac biomarkers in women with preeclampsia compared to normotensive controls at diagnosis and at one year postpartum (GO PROVE)
- To examine vascular dysfunction through measurement of peripheral resistance and blood-based endothelial biomarkers in women with preeclampsia compared to normotensive controls at diagnosis and at one year postpartum (GO PROVE)
- To examine coagulation profiles through blood-based biomarkers and tests such as thromboelastometry (ROTEM®) and impedance aggregometry (Multiplate®) in women with preeclampsia compared to normotensive controls (GO PROVE) To increase the knowledge and understanding of women's and their partners' experiences of preeclampsia during pregnancy, birth and postpartum through validated measurements and qualitative interviews (GO PROVE)

**Population**

For peer review only

Pregnant women ≥18 years of age with a diagnosis of preeclampsia after gestational week 20, presenting at Sahlgrenska University Hospital (10 500 deliveries/year), Uppsala University Hospital (4 200 deliveries/year) and Södra Älvsborgs Hospital (3 100 deliveries/year) are approached for inclusion. Both participating women and their partner are eligible for the qualitative interview study. Healthy women ≥18 years with normotensive pregnancies are included as controls.

Exclusion criteria: Women unable to understand oral and written information and unable to give written informed consent are not eligible as well as women with pre-existing hypertension, diabetes mellitus before or during pregnancy and pre-existing cardiovascular, renal or cerebral disease. MR contraindications for MR group includes claustrophobia. Women who have previously been included in the study will not be eligible for their next pregnancy.

For the database and biobank as a whole there will be no matching. For specific sub-studies there might be matching on a group level for gestational age at examination.

The diagnosis of preeclampsia will be established according to Swedish guidelines for hypertensive disorders in pregnancy (www.sfog.se) that generally follows international guidelines such as ISSHP, NICE and ACOG with the addition that we will require proteinuria to be present to limit the heterogeneity of the population.

**GO PROVE and UP MOST database**

The database is provided by MedSciNet (MedSciNet AB, Stockholm, Sweden) and includes exposure and outcome variables as proposed for preeclampsia research by the international collaborative group for preeclampsia research Co-Lab.[7] In addition, specific variables for this study have been included (Table S1 and Table 1). Biochemical tests when performed in routine health care are also collected, in addition to biochemical tests analyzed specifically for the study, and are presented in Table 2.



For peer review only - http://bmjopen.bmj.com/site/about/guidelines.xhtml

**GO PROVE and UP MOST Biobank**

GO PROVE and UP MOST use the Hospital Integrated Biobank (SIB, www.biobanksverige.se) that enables samples to be handled in a standardized manner through the hospitals' clinical laboratories, thus ensuring sample quality. Samples are sent to the local hospital's clinical chemistry laboratory or directly to the hospital biobank facility, where they are spun, aliquoted into 225 µl wells and frozen within four to six hours. Samples are only thawed directly prior to analysis. Each new project utilizing the biobank requires ethical approval, but no additional informed consent. At inclusion in the study, at delivery and at one year follow up, maternal blood and urine are collected. At delivery, placental tissue, umbilical cord blood sample and cerebrospinal fluid are collected in addition to above. Cerebrospinal fluid is only collected if spinal anasthesia is administered. If the woman undergoes MRI brain, an extra blood sample is drawn. A detailed description of samples can be found in Table 3. By merging the participants in the biobank with the GO PROVE and UP MOST database, a data- and biobank is created that will also include results from organ-specific evaluation as described below.

Bio-samples will be analyzed for pre-specified biomarkers such as cerebral biomarkers, angiogenic biomarkers, cardiac biomarkers and endothelial biomarkers. In addition, bio-samples will be available for future analyses of other potential biomarkers within the field of preeclampsia by the authors and other research groups after ethical approval and approval from the research group.

**GO PROVE and UPMOST examinations**

For an overview of included examinations, please see Figure 1.

*Magnetic resonance (MR) examination of the brain, assessment of cerebral autoregulation and cognitive function scoring*

MR brain

For peer review only

Investigations will be performed using 3T MR scanners at inclusion and at one year postpartum for cases and controls. Imaging protocols contain the following sequences: 3D T1-weighted (w) MPRAGE sequence, 3D fluid attenuated inversion recovery (FLAIR), and 2D diffusion weighted imaging (DWI) with three directions and multiple b-values for morphological evaluation of e.g. edema and gross pathology as well as perfusion assessment using intravoxel incoherent motion (IVIM) technique,[10] 2D DWI with 32 directions to evaluate white matter microstructure,[11] arterial spin labeling (ASL) for perfusion assessment,[10,12] 3D time of flight (TOF) MR angiography for vessel evaluation, susceptibility weighted imaging (SWI/QSM or SWAN) for assessment of hemorrhage and resting state functional MR (rsfMR) for evaluation of neuronal networks.[13]

Neurocognitive assessment

To determine whether the participating women experience cognitive impairment, several instruments that measure, both subjective and objective cognition, provide a fine-grained picture of the participants' cognitive performance. Subjective cognitive function is assessed by the Cognitive Failure Questionnaire (CFQ) measuring self-reported cognitive failures in everyday life.[14] The objective cognitive test battery consists of 11 tests covering a broad range of cognitive abilities. Verbal ability is assessed with a Swedish four-alternative multiple-choice synonym test (SRB 1) and serves as an estimate of premorbid cognitive ability.[15] Mental speed is measured by Digit Symbol Coding from Wechsler Adult Intelligence Scale (WAIS IV).[16] Working memory is assessed by the letter-number sequencing task and the three digit span tasks forward, backward and sequencing from WAIS-IV.[16] Executive functions are measured by three tasks from the Delis–Kaplan Executive Function System (D-KEFS) (a) Mental shifting/flexibility with the Trail Making Test; (b) Inhibition with the Stroop test; and (c) Verbal fluency with Letter and Category fluency test.[17] Several aspects of episodic memory performance are measured with free and

For peer review only



For peer review only - http://bmjopen.bmj.com/site/about/guidelines.xhtml

delayed recall of nouns using the Buschke Selective Reminding procedure and prospective episodic memory with the Reminding task.[18,19] All cognitive tests have good psychometric properties. The tests are administered by study personnel, at inclusion at the hospital and again at one year postpartum.

Transcranial cerebral doppler

Measurement of the blood flow velocity in the middle cerebral artery (MCA) is performed with transcranial doppler technique and combined with continuous non-invasive measurement of the blood pressure using finger arterial volume clamping (calibrated with brachial blood pressure measurement) and carbon dioxide concentrations in exhaled air. The signal is obtained through the temporal window and systolic, mean and diastolic blood flow velocity and depth of measurement are registered. The measurements are performed by trained investigators and intra- and inter-observer variation is measured and kept below 15%. Cerebral autoregulation is determined from the response of the cerebral blood flow velocity to spontaneous fluctuations in mean blood pressure as described previously and reported as Autoregulation Index.[20] The measurements will be compared between cases and normotensive controls and between inclusion and at one year postpartum.

*Echocardiography and endothelial dysfunction measurement*

Echocardiography

Women with preeclampsia and pregnant controls are examined with transthoracic echocardiography (TTE) for evaluation of systolic and diastolic function. Systolic dysfunction is defined as having an ejection fraction less than 50% or having regional hypokinesia. Diastolic dysfunction is defined as fulfilling at least 50% of the following; an average peak velocity of early diastolic transmitral flow / peak velocity of early diastolic mitral annular motion as determined by pulsed wave Doppler (E/e´) >14, septal e´ velocity < 7cm/s or lateral e´ velocity < 10cm/s, tricuspid regurgitation velocity > 2.8m/s or left atrial

For peer review only

volume index $>34 ml/m^2$[21]. Right ventricular function is assessed by eye-balling, tricuspid Annular Plane Systolic Excursion (TAPSE) and right ventricular peak velocity of systolic mitral annular motion as determined by pulsed wave Doppler (RV S′).

The examination is performed in proximity of inclusion and one year postpartum.

Peripheral artery resistance measurements

The status of the peripheral vessels can be measured with a variety of methods. In common, they are based on the interpretation of pulse pressure curve in the vessels. In this study we use the Reactive Hyperemia-Peripheral Arterial Tonometry (RH-PAT) method. The RH-PAT induces ischemia in one arm during 5 minutes. The pulse pressure wave in the peripheral artery, i.e. the finger, is measured before, during and after ischemia in both arms. The difference in reaction is recorded and reflects the vascular status peripherally.[22]

The Systemic Vascular Resistance index (SVRI) is calculated from the cardiac output and estimation of central venous pressure (CVP) measured through TTE indexed to body mass. SVRI reflects the afterload i.e. the workload that the heart has to handle at a normovolemic state which in turn will be affected by the patient's blood pressure and peripheral vasoconstriction.[23]

The measurements of the heart function and the derived Systemic Vascular resistance retrieved from the TTE will then be compared with the measurement of peripheral vascular function as measured by the RH-PAT method and serum biomarkers clinically used to measure heart failure. The examination is performed at inclusion and again at one year postpartum.

*Hemostasis*

Hemostatic status can be assessed by laboratory analyses and by point-of-care devices as thromboelastometry (ROTEM®) and impedance aggregometry (Multiplate®). ROTEM® is an established viscoelastic method for hemostasis testing in whole blood and a modification of

For peer review only



For peer review only - http://bmjopen.bmj.com/site/about/guidelines.xhtml

traditional thromboelastography (TEG®). ROTEM® investigates the interaction of coagulation factors, their inhibitors, anticoagulant drugs, blood cells (specifically platelets) during clotting and subsequent fibrinolysis.[24] Multiplate® measures platelet function through impedance aggregometry.[25] ROTEM® and Multiplate® are assessed at inclusion and again at delivery (GO-PROVE).

**Women's and partner's experiences of preeclampsia during pregnancy, childbirth and postnatal care**

*Questionnaires*

Effects of preeclampsia on women's self-reported health are measured with validated instruments sent to them by email, 8-12 weeks postpartum: Self-efficacy, the belief that one is capable of accomplishing a behavior or developing a competency to succeed in reaching a specific or more general goal, is measured by the General Self-Efficacy scale (GES).[26] Anxiety and depression are measured by Hospital anxiety and depression scale (HADS)[27] and Edinburgh postnatal depression scale (EPDS).[28] Health-related quality of life is determined by the EQ-visual analog scale (EQ- VAS).[29] Childbirth experiences is measured by the Childbirth Experience Questionnaire (CEQ)[30] and levels of breastfeeding self-efficacy is assessed through the Breastfeeding Self-Efficacy Scale short form (BES).[31] Post-Traumatic Stress Symptoms is evaluated by the Post traumatic stress disorder (PTSD) Checklist, DSM-5 (PCL-5).[32]

*Interviews*

Experiences of preeclampsia during pregnancy, childbirth and postnatal care are described through qualitative interviews. Informants will be 15-20 women who have experienced preeclampsia with severe features during pregnancy and their partners.[33] Informants are selected to ensure a broad range of views and experiences of the phenomenon preeclampsia. Face-to-face interviews[34,35] are conducted 2 to 3 months after delivery. An open–ended



For peer review only - http://bmjopen.bmj.com/site/about/guidelines.xhtml

question is used, “Please tell me of your experience of preeclampsia” or “please tell me of your experience as a partner to a woman with preeclampsia”.

**Data processing, analysis and statistics**

Results from cerebral doppler, echocardiography, and RH-PAT estimations will be calculated by two independent interpreters blinded to groups and entered manually into the database. MR data will be analyzed in collaboration by neuroradiologists, blinded to groups. Blood samples will be thawed for analyses of cardiac-, renal-, neurological- and endothelial biomarkers, analyzed through standardized platforms when available and for the remaining through manual ELISA analyses in duplicates. Inter- and intra-assay coefficients of variation will be aimed at below 10%.

Demographics will be presented as medians or means as appropriate by distribution and by numbers and percentages as appropriate. Comparison between groups will be analyzed by student’s t-test or Mann Whitney U-test with means or medians and confidence intervals or interquartile range, as appropriate according to distribution of the variables. Proportions will be analyzed through Chi Square test. Correlations will be analyzed by Pearson’s r or Spearman’s rho as appropriate by distribution of the variable. Regression analyses, unadjusted and adjusted, will be performed to adjust for known confounding variables. Ten cases per variable at the lowest will be considered appropriate to avoid overfitting of the model. All statistical analyses will be performed in SPSS or R. Analyzes of cognitive function will be performed through ANOVA AND MANOVA.

Data analysis of the interview data will be conducted by phenomenology with a lifeworld approach[36] or with qualitative content analysis.[37] NVivo 10 software (qsrinternational.com/) is used for grouping and organizing the text.

**Power calculations**



For peer review only - http://bmjopen.bmj.com/site/about/guidelines.xhtml

Uppsala and Sahlgrenska University hospitals have 4,200 and 10,500 deliveries yearly respectively and Södra Älvsborgs Hospital 3,100 deliveries yearly. Preeclampsia affects 3% of the population resulting in approximately 530 cases yearly at the centers and approximately 410 cases after exclusions according to the study protocol. For logistical reasons, all women cannot be included. Of eligible, we estimate that we will recruit around 20% for MR, transcranial doppler and cognition examinations (70/year) at Uppsala and Sahlgrenska University hospitals and about 40/year at Sahlgrenska University hospital for cardiac and endothelial function tests. Therefore, we estimate that inclusion will take two years. Inclusion during two years at two centers will thus give around 140 MR investigations, 100 transcranial doppler and cognition examinations and 40 TTE. We will include 60 controls for each investigation. For blood based tests we will use the whole cohort. We want to include more cases than controls since we want to be able to subtype cases based on preeclampsia phenotypes. Some specific power calculations:

*Cerebral blood flow regulation and tissue perfusion:* in order to demonstrate a change of 3 ml / min x 100g in perfusion in the cortical sub-volume of the brain,[38] 44 cases of preeclampsia and 44 normotensive controls with healthy pregnancies are required. In order to detect a difference in dynamic cerebral autoregulation index of 1.2,[20] a sample size of 32 women in each group is estimated to be required. In addition, differences in cerebral perfusion and dynamic cerebral autoregulation have been detected in the above sample sizes of 44 and 20 preeclampsia cases respectively with an equal number of normotensive controls.

*Cognition:* In a meta-analysis, a difference in the objective test “letter number sequencing task” was found when pooling results from 101 women with previous preeclampsia and 98 women with previous healthy pregnancies.[39]

**Patient and Public Involvement**

For peer review only

To ensure that the research is relevant, acceptable and feasible from the women's and partners' point of view patient representatives were invited to participate in the research process.[40] Focusing on women's and their partners' experiences of preeclampsia diagnosis and care at the same time will ensure that findings from this project will improve not only clinical outcome measures but also women's and their partners' experience.

## ETHICS AND DISSEMINATION

This is a cohort study with a control group with no intervention for the participants. Incidental pathological findings on examinations performed are and will be handled in accordance with clinical routine. Data is stored in a secure online database. Biobank samples are identified through the women's personal identification number and pseudonymised after identification in the biobank before analysis. Results from the study will be published in international peer-reviewed journals.

For peer review only

## DISCUSSION

There is a need for more individualized treatment of women with preeclampsia with respect to different organ manifestations of the disease. This in order to individualize treatment and care, such as an informed decision to deliver and to avoid short- and long-term complications for the mother and infant. Currently there are no reliable methods in clinical practice to evaluate organ dysfunction such as cardiovascular and cerebral effects of preeclampsia, although cerebral edema, intracranial bleeding and pulmonary edema secondary to heart failure are the most common causes of maternal mortality due to preeclampsia. There are emerging biomarkers and prediction models but none of them are used in routine care yet and they do not seem to be able to differentiate between different organ dysfunction.[41,42]



For peer review only - http://bmjopen.bmj.com/site/about/guidelines.xhtml

The interdisciplinary team of obstetricians, midwives, anesthesiologists, neuroradiologists and cardiologists enables us to link organ specialists in the field of preeclampsia research. This collaboration will enable important discoveries that have great potential to improve the care of these women. In addition, by employing a database created to harmonize data collection in preeclampsia research, biological samples and data can be shared with other research groups, enabling larger datasets for rare outcomes.[7] Prospective recruitment may imply selection bias. In this study, inclusions will mainly occur daytime by research personnel even though medical staff have the opportunity to include women after office hours (between 4 p.m and 8 a.m.). This might introduce selection bias regarding severity of disease where women with sudden onset of organ complications are more often seen and delivered after office hours. In addition, there might be a larger proportion of women with early onset preeclampsia recruited since women with late onset preeclampsia are induced shortly after diagnosis. However, national health records are available to compare demographics of the study population to the general population with preeclampsia.

Regarding cerebral dysfunction in preeclampsia, we will be able to further characterize imaging abnormalities, circulating cerebral biomarkers and cognitive function among women with preeclampsia and neurological impairment. Some women with preeclampsia show evidence of cerebral edema in the acute phase[43,44] and long-term follow-up of women with previous preeclampsia demonstrate an increased number of white matter lesions.[45] There is a paucity in data regarding the connection between acute lesions and long-term effects on the brain and their implications in the development of stroke, epilepsy, and dementia. Cerebral biomarkers have been reported to be increased in preeclampsia but the connection to objective findings on MR or cognitive function is not clear.[46,47] Hopefully, data from this study can enable an objective assessment of neurological function in preeclampsia as a tool for targeting women at risk for adverse cerebral events. In addition, the long-term cerebral



For peer review only - http://bmjopen.bmj.com/site/about/guidelines.xhtml

effects of preeclampsia are still an area that needs more research. There are reports of an increased risk of dementia, stroke and epilepsy after a pregnancy complicated by preeclampsia but the causality and importance of preeclampsia as an insult to the brain is not fully elucidated.[48-50] Cognitive impairment years after a pregnancy complicated by preeclampsia has been reported but it is not known whether this cognitive impairment exists already at diagnosis and how it changes over the first year postpartum after preeclampsia.[51,52] Findings from this study might increase the understanding of the underlying pathophysiological mechanisms of acute cerebral injury and long-term effects.

Regarding hemostasis, our results will hopefully provide guidance as to when women with preeclampsia can receive spinal anesthesia and further insight into the coagulation profile among women with severe preeclampsia.

For preeclampsia and cardiovascular function, an initial assessment of cardiovascular function with TTE and peripheral resistance measurement for assessment of endothelial dysfunction for all women in the study will aid in the risk-classification of women stratified on cardiovascular involvement.

Increased knowledge about how women as well as partners experience preeclampsia and the consequences could improve personalized care and follow-up after preeclampsia.

Preeclampsia is a disease only manifested in humans, hence there are no animal models of preeclampsia with the ability to exactly mimic the true preeclampsia state.[53] Therefore, it is imperative that critical laboratory and clinical observations are made on humans and human tissues. By creating a data- and biobank, we will be able to make important laboratory discoveries that can be translated to clinical trials that may improve the care of women.



For peer review only - http://bmjopen.bmj.com/site/about/guidelines.xhtml

**LIST OF ABBREVIATIONS**

BES Breastfeeding Self-Efficacy Scale short form

CEQ Childbirth Experience Questionnaire

CFQ Cognitive Failures Questionnaire

D-KEFS Delis-Kaplan Executive Function System

DSM 5 (PCL-5) Post Traumatic Stress Disorder Checklist

DWI Diffusion weighted images

EPDS Edinburgh Postnatal Depression Scale

EQ-VAS Euro Qol Visual Analogue Scale

FLAIR Fluid attenuated inversion recovery

GES General Self-Efficacy Scale

GO-PROVE Gothenburg Obstetric Adverse Events

HADS Hospital Anxiety and Depression Scale

MCA Middle cerebral artery

MRA 3D TOF Magnetic resonance angiography 3 dimensional time of flight

MR Magnetic Resonance

PROVE Preeclampsia Obstetric Adverse Events

PTSD Post traumatic stress disorder

RH-PAT reactive hyperemia-peripheral arterial tonometry

ROTEM Rotational tromboelastometry

SIB Hospital integrated biobank

SU Sahlgrenska University Hospital

TEG Thromboelastography

UP-PROVE Uppsala Pregnancy Complication Study

WAIS IV Wechsler Adult Intelligence Scale, fourth version

For peer review only

# DECLARATIONS

## Ethics approval and consent to participate

This study was approved by the regional ethical review board in Gothenburg on the 28th December 2018 (approval number 955-18) and by the Swedish Ethical Review Authority on the 27th of February 2019 (approval number 2019-00309).

All participating women are given oral and written information and sign an informed consent before participation in the study.

## Consent for publication

Not applicable.

## Availability of data and materials

The datasets used and/or analyzed during the current study are available from the corresponding author on reasonable request.

## Acknowledgment

We would like to thank Biobank Väst and Uppsala Biobank (a part of SIB) for supporting with collection of biological specimens.

For peer review only

## REFERENCES

1. Mol BWJ, Roberts CT, Thangaratinam S, Magee LA, de Groot CJM, Hofmeyr GJ. Pre-eclampsia. *Lancet.* 2016;387(10022):999-1011.

2. Duley L. The global impact of pre-eclampsia and eclampsia. *Semin Perinatol.* 2009;33(3):130-137.

3. Lain KY, Roberts JM. Contemporary concepts of the pathogenesis and management of preeclampsia. *JAMA.* 2002;287(24):3183-3186.

4. von Dadelszen P, Magee LA, Roberts JM. Subclassification of preeclampsia. *Hypertension in pregnancy : official journal of the International Society for the Study of Hypertension in Pregnancy.* 2003;22(2):143-148.

5. Brown MA, Magee LA, Kenny LC, et al. The hypertensive disorders of pregnancy: ISSHP classification, diagnosis & management recommendations for international practice. *Pregnancy Hypertens.* 2018.

6. Sibai BM. Preeclampsia as a cause of preterm and late preterm (near-term) births. *Seminars in Perinatology.* 2006;30(1):16-19.

7. Myatt L, Redman CW, Staff AC, et al. Strategy for standardization of preeclampsia research study design. *Hypertension.* 2014;63(6):1293-1301.

8. Furuta M, Sandall J, Bick D. Women's perceptions and experiences of severe maternal morbidity--a synthesis of qualitative studies using a meta-ethnographic approach. *Midwifery.* 2014;30(2):158-169.

9. Vaerland IE, Vevatne K, Brinchmann BS. An Integrative Review of Mothers' Experiences of Preeclampsia. *J Obstet Gynecol Neonatal Nurs.* 2016;45(3):300-307.

10. Le Bihan D, Breton E, Lallemand D, Aubin ML, Vignaud J, Laval-Jeantet M. Separation of diffusion and perfusion in intravoxel incoherent motion MR imaging. *Radiology.* 1988;168(2):497-505.

For peer review only



For peer review only - http://bmjopen.bmj.com/site/about/guidelines.xhtml

11. Pierpaoli C, Jezzard P, Basser PJ, Barnett A, Di Chiro G. Diffusion tensor MR imaging of the human brain. *Radiology.* 1996;201(3):637-648.

12. Williams DS, Detre JA, Leigh JS, Koretsky AP. Magnetic resonance imaging of perfusion using spin inversion of arterial water. *Proc Natl Acad Sci U S A.* 1992;89(1):212-216.

13. Biswal B, Yetkin FZ, Haughton VM, Hyde JS. Functional connectivity in the motor cortex of resting human brain using echo-planar MRI. *Magn Reson Med.* 1995;34(4):537-541.

14. Broadbent DE, Cooper PF, FitzGerald P, Parkes KR. The Cognitive Failures Questionnaire (CFQ) and its correlates. *Br J Clin Psychol.* 1982;21 (Pt 1):1-16.

15. Dureman I KL, Österberg E. *Manual till DS-batteriet [Manual for the DS-battery].* Stockholm: Psykologiförlaget; 1971.

16. Wechsler D. *WAIS-IV Administration and Scoring Manual.* San Antonio, TX: The Psychological Corporation; 2008.

17. Delis DC Kaplan E KJ. *Delis-Kaplan Executive Functioning System (D-KEFS).* San Antonio: The Psychological Corporation; 2001.

18. Buschke H. Selective reminding for analysis of memory and learning. *J Verb Learn Verb Behav.* 1973;12:543–550.

19. Mäntylä T, Nilsson LG. Remembering to remember in adulthood: A population-based study on aging and prospective memory. *Journal of Neuropsyhol Cogn.* 1997;4:81-92.

20. van Veen TR, Panerai RB, Haeri S, Griffioen AC, Zeeman GG, Belfort MA. Cerebral autoregulation in normal pregnancy and preeclampsia. *Obstet Gynecol.* 2013;122(5):1064-1069.

21. Nagueh SF SO, Appleton CP, Byrd BF 3rd, Dokainish H, Edvardsen T, Flachskampf FA, Gillebert TC, Klein AL, Lancellotti P, Marino P, Oh JK, Popescu BA, Waggoner

For peer review only



For peer review only - http://bmjopen.bmj.com/site/about/guidelines.xhtml

AD. Recommendations for the Evaluation of Left Ventricular Diastolic Function by Echocardiography: An Update from the American Society of Echocardiography and the European Association of Cardiovascular Imaging. *Journal Am Soc Echocardiogr.* 2016;29(4):277-314.

22. Meeme A BG, Mammen M, Namugowa A. Endothelial dysfunction and arterial stiffness in pre-eclampsia demonstrated by the EndoPAT method. *Cardiovascular Journal of Africa.* 2016;27:1-6.

23. Capeless EL CJ. Cardiovascular changes in early phase of pregnancy. *American Journal of Obstetrics and Gynecology.* 1989;161(6):1449-1453.

24. RJ L. Thrombelastography/thromboelastometry. *Clin Lab Haematol.* 2005;27(2):81-90.

25. Moenen FCJI VM, Nelemans PJ, van Rooy KJM, Vranken JRRA, Verhezen PWM, Wetzels RJH, Ten Cate H, Schouten HC, Beckers EAM, Henskens YMC. Screening for platelet function disorders with Multiplate and platelet function analyzer. *Platelets.* 2019;30(1):81-87.

26. Love J, Moore CD, Hensing G. Validation of the Swedish translation of the General Self-Efficacy scale. *Qual Life Res.* 2012;21(7):1249-1253.

27. Spinhoven PH OJ, Sloekers PPA, Kempen GIJM, Speckens AEM, Van Hemert AM. A validation study of the Hospital Anxiety and Depression Scale (HADS) in different groups of Dutch subjects. *Psychological Medicine.* 1997;27:363-370.

28. Cox JL HJ, Sagovsky R. Detection of Postnatal Depression Development of the 10-item Edinburgh Postnatal Depression Scale. *British Journal of Psychiatry.* 1987;150:782-786.

29. Group TE. EuroQol - a new facility for the measurement of health-related quality of life. *Health Policy.* 1990;16:199-208.

For peer review only



For peer review only - http://bmjopen.bmj.com/site/about/guidelines.xhtml

30. Dencker A, Taft C, Bergqvist L, Lilja H, Berg M. Childbirth experience questionnaire (CEQ): development and evaluation of a multidimensional instrument. *BMC Pregnancy Childbirth.* 2010;10:81.

31. Gerhardsson E, Nyqvist KH, Mattsson E, Volgsten H, Hildingsson I, Funkquist EL. The Swedish Version of the Breastfeeding Self-Efficacy Scale-Short Form: Reliability and Validity Assessment. *J Hum Lact.* 2014;30(3):340-345.

32. Blevins CA, Weathers FW, Davis MT, Witte TK, Domino JL. The Posttraumatic Stress Disorder Checklist for DSM-5 (PCL-5): Development and Initial Psychometric Evaluation. *J Trauma Stress.* 2015;28(6):489-498.

33. Patton M. *Qualitative Research& Evaluation Methods (3rd ed.).* Thousand Oaks,CA,: Sage Publications; 2002.

34. Graneheim U LB. Qualitative content analysis in nursing research: Concepts, procedures and measures to achieve trustworthiness. *Nurse Education Today.* 2004;24:105-112.

35. Polit D BC. *Nursing Research, Generating and Assessing Evidence fo Nursing Practice.* 8th ed: Wolters Kluwer/Lippincott Williams&Wilkins; 2008.

36. Dahlberg K DH, Nyström Pettersson M. *Reflective Lifeworld Research.* Studentlitteratur Lund; 2008.

37. Elo S KH. The qualitative content analysis process. *Journal of Advanced Nursing.* 2008;62(1):107-115.

38. Virhammar J, Laurell K, Ahlgren A, Larsson EM. Arterial Spin-Labeling Perfusion MR Imaging Demonstrates Regional CBF Decrease in Idiopathic Normal Pressure Hydrocephalus. *AJNR Am J Neuroradiol.* 2017;38(11):2081-2088.

For peer review only



For peer review only - http://bmjopen.bmj.com/site/about/guidelines.xhtml

39. Elharram M, Dayan N, Kaur A, Landry T, Pilote L. Long-Term Cognitive Impairment After Preeclampsia: A Systematic Review and Meta-analysis. *Obstet Gynecol.* 2018;132(2):355-364.

40. Sacristan JA, Aguaron A, Avendano-Sola C, et al. Patient involvement in clinical research: why, when, and how. *Patient Prefer Adherence.* 2016;10:631-640.

41. Thangaratinam S, Allotey J, Marlin N, et al. Prediction of complications in early-onset pre-eclampsia (PREP): development and external multinational validation of prognostic models. *BMC Med.* 2017;15(1):68.

42. Cerdeira AS, O'Sullivan J, Ohuma EO, et al. Randomized Interventional Study on Prediction of Preeclampsia/Eclampsia in Women With Suspected Preeclampsia. *Hypertension.* 2019:HYPERTENSIONAHA11912739.

43. Brewer J, Owens MY, Wallace K, et al. Posterior reversible encephalopathy syndrome in 46 of 47 patients with eclampsia. *Am J Obstet Gynecol.* 2013;208(6):468 e461-466.

44. Fisher N, Saraf S, Egbert N, Homel P, Stein EG, Minkoff H. Clinical Correlates of Posterior Reversible Encephalopathy Syndrome in Pregnancy. *J Clin Hypertens (Greenwich).* 2016;18(6):522-527.

45. Aukes AM, De Groot JC, Wiegman MJ, Aarnoudse JG, Sanwikarja GS, Zeeman GG. Long-term cerebral imaging after pre-eclampsia. *BJOG.* 2012;119(9):1117-1122.

46. Bergman L, Zetterberg H, Kaihola H, Hagberg H, Blennow K, Akerud H. Blood-based cerebral biomarkers in preeclampsia: Plasma concentrations of NfL, tau, S100B and NSE during pregnancy in women who later develop preeclampsia - A nested case control study. *PLoS One.* 2018;13(5):e0196025.

47. Evers KS, Atkinson A, Barro C, et al. Neurofilament as Neuronal Injury Blood Marker in Preeclampsia. *Hypertension.* 2018;71(6):1178-1184.

For peer review only



For peer review only - http://bmjopen.bmj.com/site/about/guidelines.xhtml

48. Basit S, Wohlfahrt J, Boyd HA. Pre-eclampsia and risk of dementia later in life: nationwide cohort study. *BMJ.* 2018;363:k4109.

49. Miller EC. Preeclampsia and Cerebrovascular Disease. *Hypertension.* 2019;74(1):5-13.

50. Nerenberg KA, Park AL, Vigod SN, et al. Long-term Risk of a Seizure Disorder After Eclampsia. *Obstet Gynecol.* 2017;130(6):1327-1333.

51. Aukes AM, Wessel I, Dubois AM, Aarnoudse JG, Zeeman GG. Self-reported cognitive functioning in formerly eclamptic women. *Am J Obstet Gynecol.* 2007;197(4):365 e361-366.

52. Postma IR, Groen H, Easterling TR, et al. The brain study: Cognition, quality of life and social functioning following preeclampsia; An observational study. *Pregnancy Hypertens.* 2013;3(4):227-234.

53. Sunderland N, Hennessy A, Makris A. Animal models of pre-eclampsia. *Am J Reprod Immunol.* 2011;65(6):533-541.

For peer review only

## Figure legends

**Figure 1.**

Flow chart of the study. Investigations and biological samples from inclusion to one year postpartum. CSF; Cerebrospinal fluid, MRI; Magnetic resonance imaging

For peer review only



For peer review only - http://bmjopen.bmj.com/site/about/guidelines.xhtml

**Authors' contributions**

LB and LT wrote the article. LB contributed with information about biosamples in GO PROVE, transcranial doppler examination, MRI examinations, structure of the online database and inclusion of women in GO PROVE. LT contributed with information about questionnaires, cognitive tests, handling of biosamples and structure of the online database. MA, JO and SET provided the methods and contributed to the sections regarding cardiac function and endothelial dysfunction. OK provided the methods and contributed to the sections regarding coagulation. VS contributed to the section about recruitment of the population. TS and SL contributed to the methods section about placental sampling. HE and KL provided the methods and contributed to the sections regarding women's experience of childbirth and questionnaires. KJ and TVV contributed to the sections about transcranial doppler and cerebral autoregulation. IE contributed to the section about recruitment of particpants in Uppsala (UPMOST). JW and IBB provided the methods section and contributed to the sections about MRI. ASN provided the methods section and contributed to the sections about cognitive function tests. AKW contributed to the section about recruitment in Uppsala and handling of biosamples.

All authors revised and approved the final version of the manuscript.

**Funding statement**

This study is supported by the Swedish Brain Fund, Märta Lundqvist's foundation, Jane and Dan Olsson's foundation, the Foundation of the Health and Medical care committee of the Region of Västra Götaland, Sweden (VGFOUREG851391), (VGFOUREG930918), Gothenburg Society of Medicine (GLS934555 and GLS878481), Sahlgrenska University Hospital, ALF Västra Götalands Region (ALFGBG-925851), Swedish Research Council (2020-01640), Swedish Society for Medical Research and Sahlgrenska University Hospital SU (2018-03591).



For peer review only - http://bmjopen.bmj.com/site/about/guidelines.xhtml

**Competing interest statement**

The authors declare that they have no competing interests.

For peer review only



For peer review only - http://bmjopen.bmj.com/site/about/guidelines.xhtml

For peer review only



For peer review only - http://bmjopen.bmj.com/site/about/guidelines.xhtml

**Table 1.** Neurological Symptoms at inclusion, at MRI brain and at follow up one year postpartum.

| |
|---|
| Edema (n) |
| Visual disturbances (b) |
| *No visual disturbances* (b) |
| *Blurred vision* (b) |
| *Double vision* (b) |
| *Temporary loss of vision* (b) |
| *Sensitivity to light* (b) |
| *Partial loss of vision or blind spots in normal field of vision* (b) |
| *Flickering lights* (b) |
| *Other* (n) |
| |
| Time of onset for visual disturbances (n) |
| *N/A (no visual disturbance)* (b) |
| |
| Headache (o) |
| Headache onset (o) |
| Epigastric/abdominal pain (o) |
| Tightness in chest (b) |
| Shortness of breath (b) |
| Focal neurological deficits (n) |
| |
| Tendon reflexes (n) |
| Nausea (b) |
| Vomit (b) |
| Form of confusion (b) |
| Twitching or jerking in arms and/or legs (b) |
| Has the woman's family/friends experienced the patient as not responding or staring blankly into space (b) |
| Difficulty concentrating (b) |
| Speech affected (b) |
| Hearing affected (b) |
| Mood changes (n) |
| Other mood changes than listed (Free text) |
| |
| *Before being diagnosed with PE/MRI scan/visit at 1 year postpartum did the woman* |
| -feel anxious (b) |
| -feel as if the end of the world was coming (b) |
| -experience severe dizziness (b) |
| -experience any weakness or paralysis (b) |
| -experience jitters/nervousness/nervous shaking (b) |
| -experienced any other symptoms not listed (b) |
| List other symptoms (FREE text) |

For peer review only



For peer review only - http://bmjopen.bmj.com/site/about/guidelines.xhtml

655 **Table 2.** Biochemical analyses specific for the study and in clinical routine when available.

| Prenatal screening | At inclusion | At delivery and Summary of analyses at discharge* |
|---|---|---|
| **If taken in clinical routine**<br>human chorionic gonadotropin) (c)<br>Oestriol (c)<br>Alpha-fetoprotein (c)<br>Inhibin A (c)<br>Pregnancy-associated plasma protein A (c)<br>Cell free fetal DNA (c) | **If taken in clinical routine**<br>Haemoglobin (c)<br>Haematocrit (c)<br>WCC (white cell count) (c)<br>Platelets (c)<br>Creatinine (c)<br>AST (aspartate transaminase) (c)<br>ALT (alanine transaminase) (c)<br>Total bilirubin (c)<br>Albumin (c)<br>LDH (lactate dehydrogenase) (c)<br>Haptoglobin (c)<br>APTT (activated partial thromboplastin time) (c)<br>PR (prothrombin ratio) (c)<br>INR (International normalize ratio) (c)<br>CRP (C reactive protein) (c)<br>Oxygen saturation (d)<br>Total leukocyte count (c)<br>Urinary prot/creat ratio (c)<br>Fibrinogen (c)<br>Antitrombin (c)<br><br>**Specific for the study**<br>ROTEM (b)<br>Multiplate (b)<br>NT-pro BNP (c)<br>Troponin (c) | **At delivery specific for the study**<br>ROTEM (b)<br>Multiplate (b)<br>Platelets (c)<br>Troponin (c)<br>NT pro BNP (c)<br><br>**Summary of analyses at discharge if taken in clinical routine**<br>Haemoglobin (g/L) (c)<br>Platelet count (x10^9/L) (c)<br>Urinary protein/creatinine ratio (mg/g) (c)<br>Creatinine (mmol/L) (c)<br>Aspartate transaminase (IU/L) (c)<br>Alanine transaminase (IU/L) (c)<br>GGT (IU/L) (c)<br>Total bilirubin (umol/L) (c)<br>Albumin (g/L) (c)<br>LDH(IU/L) (c)<br>Haptoglobin (mg/dL) (c)<br>APTT (sec) (c)<br>INR (c)<br>D-dimer (ug/L) (c)<br>CRP (mg/L) (c) |

656 **Lowest antepartum in hospital/Highest antepartum in hospital/Lowest value postpartum/Highest value postpartum*

657

658

For peer review only



For peer review only - http://bmjopen.bmj.com/site/about/guidelines.xhtml

**Table 3.** Biological sample collection

| At inclusion | At delivery | At MRI brain | At one year postpartum |
|---|---|---|---|
| Serum | Serum | Serum | Serum |
| EDTA plasma | EDTA plasma | EDTA plasma | EDTA plasma |
| Li/Heparin plasma | Li/Heparin plasma | | |
| EDTA whole blood | EDTA plasma umbilical cord | | |
| Urine | Cerebrospinal fluid | | |
| | Placental villous tissue frozen | | |
| | Placental villous tissue RNA later | | |
| | Basal plate, full thickness frozen | | |
| | Basal plate, full thickness RNA later | | |

For peer review only

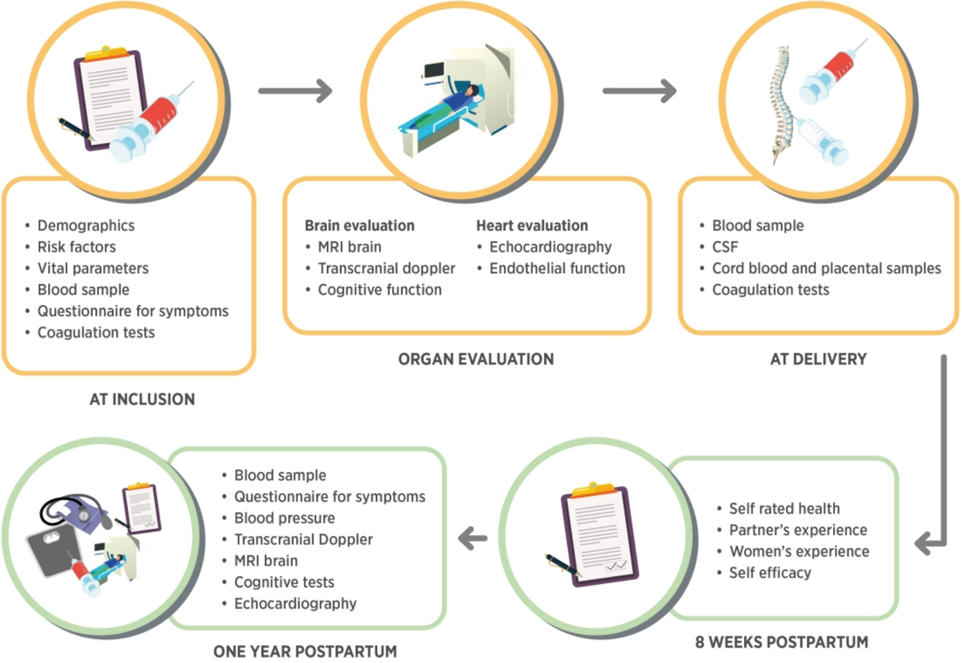


Flow chart of the study. Investigations and biological samples from inclusion to one year postpartum. CSF; Cerebrospinal fluid, MRI; Magnetic resonance imaging

297x209mm (300 x 300 DPI)

**Table S1**. Variables included in the study.

| Variables from the woman's medical chart | Variables from the study inclusion visit, gathered from the patients´ medical charts | Variables from the study inclusion visit when diagnosed with preeclampsia, registered by patients in the enrolment module | Variables from the Swedish Pregnancy Register after delivery |
|---|---|---|---|
| Marital status (n)<br>Current living situation (n)<br>Country of birth (n)<br>Total years of education (c)<br>Profession (n)<br>Maternal occupation in the beginning of pregnancy (b)<br>Height (c)<br>Intra uterine growth restriction (b)<br>Preeclampsia (b)<br><br>**Obstetric history**<br>Number of pregnancies terminated before 21+6 weeks gestation (d)<br>Previous pregnancies, terminated before 21+6 weeks gestation, causes (n)<br>Gestational age (d)<br>Gestational hypertension (b)<br>Preeclampsia (b)<br>Eclampsia (b)<br>HELLP (b)<br>Gestational diabetes treated with Insulin or oral agents (b)<br>Treatment details (Free text)<br><br>Number of pregnancies beyond 21+6 weeks gestation (d)<br>Information on previous pregnancies, with delivery beyond 21+6 weeks gestation, outcome details:<br>Gestational age (d)<br>Gestational hypertension (b)<br>Preeclampsia (b)<br>Eclampsia (b)<br>HELLP (b) | **Study assessment at inclusion**<br>Date (c)<br>Location (n)<br>1st BP (c)<br>2nd BP (c)<br>Pulse (c)<br>Weight (c)<br>Proteinuria (dipstick) (o)<br>Urinary protein/creatinine ratio (c)<br>24 hour protein (c)<br>Gestation at PE/Intra uterine growth restriction (IUGR) diagnosis (c)<br><br>**Investigations at inclusion**<br>Samples taken, see table 3<br><br>**Urine sample**<br>See Table 3<br><br>**Gestational diabetes (GDM) screening, diagnosis treatment**<br>Date screened (c)<br>Date of GDM diagnosis (c)<br>Gestation GDM diagnosed (c)<br>Date of glucose tolerance test (d)<br><br>**Ultrasound and doppler scan after preeclampsia diagnosis**<br>Date of scan (d)<br>Fetal sex (n)<br>Gestational age when scanned (c)<br>Fetal abnormality (n)<br>Crown rump length (c)<br>Nuchal translucency (c) | Yearly income (o)<br>First pregnancy with the baby's biological father (b)<br>Months of sexual relationship (pre-pregnancy) (c)<br>Contraception used in this relationship (n)<br>If contraception has been used, which mostly in the last year (n)<br>Medications before pregnancy (n)<br>First degree relative/s with hypertensive disease in pregnancy (b)<br>First degree relative/s with hypertension, Ischaemic Heart Disease, Stroke or any form of Diabetes (b)<br>Pre-pregnancy weight (c)<br><br>**Partners Family History**<br>Children with other partners (b)<br>Pregnancy outcome with other partner (n)<br>First degree relative with preeclampsia or Eclampsia (b)<br>First degree relative/s with Hypertension (b), Ischaemic Heart Disease (b), Stroke (b) or any form of Diabetes (b)<br><br>**Partner's Data**<br>Age (c)<br>Weight (c)<br>Height (c)<br>Body mass index (c)<br>Partners medical condition (n)<br>Diabetes mellitus (b)<br>Hypertension (b)<br>Chronic Heart Disease (b) | **Maternal**<br>*Delivery data*<br>Date of admission pre-delivery (c)<br>GA at admission pre-delivery (c)<br>Onset of delivery (n)<br>Maximum temperature in labour (c)<br><br>Foetal compromise pre-labour Caesarean section<br>Place and supervision of delivery (n)<br>Duration of rupture of membranes (c)<br><br>Duration of 1st stage (c)<br>Duration of 2nd stage (c)<br><br>Mode of delivery (n)<br>Indication for operative delivery (n)<br>Indication for delivery (n)<br><br>*Medication*<br>Magnesium sulphate (n)<br>Steroids (n)<br>Antihypertensive treatment in labour (n)<br>Medication during labour and delivery only (Free text)<br>Calcium antagonist (b)<br><br>*Placenta*<br>Recommended standards of placental handling used (b)<br>Placental weight (c)<br>Cord insertion relative to placental margin (n)<br>Number of vessels in cord (b)<br>Pathology report (b) |

For peer review only

| | | | |
|---|---|---|---|
| Gestational diabetes treated with Insulin or oral agents (b)<br>Treatment details (n)<br><br>**History of infertility** (b)<br>Fertility treatment for this conception (b)<br>Stimulation for ovulation for this conception (b)<br>Artificial insemination for this conception (n)<br>In Vitro fertilization for this conception (n)<br>Intra Cytoplasmic sperm injection for this conception (n)<br>Embryo recipient for this conception (b)<br><br>**Medical conditions diagnosed before pregnancy**<br>Polycystic ovary syndrome (b)<br>Medical conditions diagnosed before pregnancy (n)<br>Hypertension (b)<br>Renal disease (b)<br>Anaemia (b)<br>Diabetes type I (b)<br>Diabetes type II (b)<br>Lupus disease (b)<br>Inflammatory bowel disease (b)<br>Coeliac disease (b)<br>Rheumatoid arthritis (b)<br>Epilepsy (b)<br>Thromboembolism (b)<br>History of depression (b)<br>Antiphospholipid syndrome (b)<br>HIV (b)<br>Sickle cell (b)<br>Malaria requiring treatment in the last year (b)<br>Asthma (b)<br>Oral steroids for asthma (b)<br>Thyroid disease (n)<br>Migraine (b)<br>Irritable colon (b)<br>Anxiety disorder (b)<br>Other medical conditions diagnosed before pregnancy (free text) | Biparietal diameter (BPD (c)<br>Head circumference (HC) (c)<br>Abdominal diameter (AD (c)<br>Femur length (C)<br>Sub-chorionic haematoma (b)<br>2nd sac (b)<br>Risk score of Trisomy 21(d)<br>Liquor volume (o)<br>Placental Location (n)<br>Clinical suspicion for fetal growth restriction (b)<br>Documented fetal growth restriction (b)<br>Clinical indication for Macrosomia (b)<br>Amniotic fluid index (c)<br>Single deepest pocket (c)<br><br>*Umbilical dopplers* (c)<br>Gestational age (c)<br>Umbilical PI (c)<br><br>*Uterine dopplers*<br>L Ut Pulsatility Index (c)<br>R Ut Pulsatility Index (c)<br>Mean Ut PI (c)<br>L Notch (c)<br>R Notch (c)<br>Other Ultrasound comments (Free text)<br>Fetal Outcome (n)<br><br>**Medication use at inclusion**<br>Any medication at inclusion(b)<br>Low dose aspirin (b)<br>Other antiplatelet medication (b)<br>Low molecular weight heparin (n)<br>Diuretics (n)<br>Labetalol (n)<br>Beta blocking agent (n)<br>Calcium channel antagonist (n)<br>Antibiotic (n)<br>Anticoagulants (n)<br>Anticonvulsant-1 (not $MgSO_4$) (n)<br>Antidepressants (n) | Other (Free text)<br><br>**Vitamin and mineral supplementation before /and during pregnancy**<br>Folate (c)<br>Multivitamin (o)<br>Vitamin D (o)<br>Iron (o)<br>Calcium (o)<br><br><br>**Infection in pregnancy to record** (b)<br>Type of infection (n)<br>Urinary tract infection (lower) (b)<br>Pyelonephritis (b)<br>Human immunodeficiency virus (b)<br>Active Tuberculosis (b)<br>Zika virus (b)<br>Schistosomiasis (b)<br>Active or chronic hepatitis (b)<br>Active or recently treated sexually transmitted infection (b)<br>Other infection in pregnancy (b)<br><br>Diagnosis (n)<br>Treatment (n)<br><br>**Symptoms at inclusion see Table X**<br><br>**At time of MRI brain and Cognitive tests**<br>Blood pressure (c)<br>Pulse (c)<br>Oxygen saturation (d)<br><br>Current treatment<br>-Magnesium sulphate IVI (b)<br>-Labetolol IVI (b)<br>-Hydralazine IVI (b)<br>-Calcium antagonist short acting oral (b)<br>-Labetolol oral (b)<br>-Calcium antagonist long acting oral (b) | Photograph against a scale bar (b)<br><br>**Newborn data**<br>Liveborn (b)<br>Date of birth (c)<br>Time of birth (c)<br>Gestational age at delivery (c)<br>Sex (b)<br>Apgar score (d)<br>Intubation at birth (n)<br>Cord arterial pH (c)<br>Cord arterial Base deficit (c)<br>Cord venous pH (c)<br>Cord venous Base deficit (c)<br>Birthweight (c)<br>Birthweight centile (c)<br>Customised birthweight centile (c)<br>Length (c)<br>Head circumference (c)<br>TSH (c)<br>T3 total (c)<br>T4 total (c)<br>T3 free (c)<br>T4 free (c)<br>ICD code(s) for measures and diagnosis (n/free text)<br><br>*Neonatal Outcome at discharge*<br>Uncomplicated neonatal outcome (b)<br>Neonatal outcome (n)<br>Date of discharge home (c)<br>Days in hospital from birth to discharge home (d)<br>Admission to neonatal unit (c)<br>Final discharge date from neonatal unit or date of death in neonatal unit (c)<br>Days in neonatal unit (d)<br><br>**Perinatal outcome**<br>Perinatal Survival (b)<br>Stillbirth (b) |

For peer review only

Medications before pregnancy (n)

**Last menstrual period** (LMP)
Certainty (n)
LMP date (c)
Gestational age by LMP/clinical examination (c)
Estimated due date by LMP (c)

***Estimated due date estimated by scan***
<16 week scan date (c)
19-21 week scan date (c)
Final estimated due date (c)

**Clinical Readings at 1st Clinical Assessment**
Date of 1st clinical blood pressure (BP) (c)
Gestation of 1st BP (c)
1st clinical weight (c)
Proteinuria dipstick (o)
Urinary protein/creatinine ratio (c)
24 hour protein (c)

**Clinical Test**
(*Prenatal screening for chromosomal anomalies*)
Prenatal diagnosis (n)
Serum Screening for prenatal diagnosis (SSPD) (b)
Date of SSPD performed (c)
Gestation if SSPD performed (c)

**1st and 2nd Trimester Complications**
Hyperemesis (b)
Treatment in case of hyperemesis (free text)
Vaginal bleeding (b)
Gestational week if vaginal bleeding has occurred (c)
Duration of bleeding (d)
Amount of blood (o)

Antiglycemic agent –Insulin (b)
Metformin (b)
Long-term corticosteroids (b)
Other immunosuppressant (n)
Thyroid supplement (b)
Antithyroid treatment for thyrotoxicosis (b)
Statins (n)
Other medication(n)
Hydralazine (b)
Magnesium sulphate (b)

Received steroids for fetal lung maturation before sampling (b)
Type of steroids (n)
Date started (c)
Gestational age started (c)
Date completed (c)
Gestational age completed (c)
Total dose (c)
Time between last dose and sampling (c)

-ACE inhibitor oral (b)
-AT-2 inhibitor oral (b)
-Diuretics IVI (b)
-Diuretics oral (b)
-Other medications (b)

**Neurological symptoms at MRI brain, see table X**

**Echocardiocraphy and RH pat**
Date (c)
Blood pressure (c)
Pulse (c)
Oxygen saturation (d)
Oxygen administration (b)
Current treatment:
-Magnesium sulphate IVI (b)
-Labetolol IVI (b)
-Hydralazine IVI (b)
-Calcium antagonist short acting oral (b)
-Labetalol (b)
-Calcium antagonist long acting oral (b)
-ACE inhibitor oral (b)
-AT-2 inhibitor oral (b)
-Diuretics IVI (b)
-Diuretics oral (b)
-Other medications (b)
If *other*, state (FREE text)

**Variables from the visit at 1 year postpartum registered by the patient in the study module as well as study personnel**
Date (date)
Blood pressure (c)
Pulse (c)
Weight (c)
*Medication*
ACE inhibitor (b)
AT-2 inhibitor (b)
Diuretics (b)

Neonatal death (o)

**Final Maternal and Perinatal Outcome**
Maternal outcome (n)
Maternal admission to intensive care unit (b)

**Pregnancy Summary & Complication**
Did any of these complications occur
-Normal pregnancy (b)
-Preeclampsia (b)
-Gestational Hypertension (b)
-Eclampsia or HELLP (b)
-Chronic hypertension (b)
-Placental abruption (b)
-Maternal death (b)
-Small for gestational age by population centile (b)
-Fetal growth retardation (b)
-Spontaneous preterm birth (b)
-PPROM with spontaneous preterm birth (b)
-Cervical insufficiency (b)
-Pregestational diabetes (b)
-Gestational diabetes (b)
-Cholestasis of pregnancy (b)
-Venous thromboembolism (b)
-Obese (BMI≥30) (b)
-Postpartum haemorrhage (PPH) (b)
-Other complications (b)
Maternal outcome comment (Free text)
-Perinatal outcome (n)
Fetal outcome comment (Free text)
Autopsy examination (n)
Major congenital abnormalities (n)
Chromosomal abnormality (n)
Congenital infection (b)
*ICD codes for diagnosis*
O14.0 (b)
O14.1A (b)
O14.1B (b)
O14.1X (b)
O14.9 (b)

For peer review only

| | | | |
|---|---|---|---|
| **Recreational Drug use**<br>Any recreational drug user (n)<br>Gestation ceased (c)<br>Tobacco (o)<br>Cigars (o)<br>Chewing tobacco (o)<br>Snuff (o)<br>E-cigarettes (o)<br>Nicotine replacement (o)<br>Alcohol (o)<br><br>*Other drugs in pregnancy*<br>Marijuana (b)<br>Cocaine/Crack (b)<br>Amphetamines (b)<br>Substance P/Crystal Meth (b)<br>XTC (b)<br>Opiates (b)<br>Hallucinogens (b)<br>Other (n)<br><br>Audit number 3 months prior to pregnancy (d)<br><br>**Ultrasound**<br>Date of scan (c)<br>Fetal sex (n)<br>Gestational age (c)<br>Fetal abnormality(n)<br>CRL (crown rump length) (c)<br>BPD (biparietal diameter) (c)<br>HC (head circumference) (c)<br>AD (abdominal diameter) (c)<br>FL (femur length) (c)<br>Sub-chorionic haematoma(b)<br>2nd sac (b)<br>Risk score of Trisomi 21(d)<br>Liquor Volume (o)<br>Placental Location (n)<br>Clinical suspicion for fetal growth restriction (FGR) (b)<br>Documented fetal growth restriction (b) | | Betablocker (b)<br>Aspirin (b)<br>Insulin (b)<br>Oral diabetes medication (b)<br>Lipid lowering medication (b)<br>Levothyroxine (b)<br>Antidepressants (b)<br>Sleep medicine (b)<br><br>Did she breastfeed (b)<br>Period of breastfeeding (o)<br>Regained period (b)<br>Use of contraception (b)<br>-If yes, what contraception (free text)<br><br>After delivery, has the woman sought healthcare for;<br>-Migraine (b)<br>-High blood pressure (b)<br>-Visual disturbance (b)<br>-Diabetes (b)<br>-Thyroid function disorder (b)<br>-Allergies (b)<br>-Irritable colon (b)<br>-Alcohol or drug addiction (b)<br>-Depression (b)<br>-Anxiety (b)<br>-Pain-related disorders (b)<br><br>Cognitive test performed (b)<br>-STAI, PANAS, PSQI performed (b)<br>-EPDS, HADS performed (b)<br>-EQ-VAS, CEC, BSES, GES performed (b)<br>-Bioimpedance been performed (b)<br><br>**Neurological symptoms at 1 year postpartum, see Table X** | O11.9 (b)<br>Other (b)<br>Procedure codes (FREE text)<br><br>**Other severe complication at any time before or after delivery**<br>-None (b)<br>-Unknown (b)<br>-DIC (b)<br>-Acute renal insufficiency (b)<br>-No urine catheter output for more than 60 min (b)<br>-Haemolysis (b)<br>-CVA (b)<br>-Epigastric pain (b)<br>-Pulmonary Oedema (b)<br>-Hepatic rupture (b)<br>-Severe sepsis (b)<br>-Maternal death (b)<br>-Foetal death (b)<br>-Thrombocytopenia (b)<br>-Abnormal LFT (due to PE) (b)<br>-Coma (b)<br>-Jaundice (b)<br>-Respiratory failure (b)<br>-Cardiac failure (b)<br>-Venous Thromboembolism (b)<br>-Other complications (b)<br>-If other, specify (FREE text)<br><br>**Summary of antenatal and postnatal lab results**<br>-Blood group (n)<br>-Rhesus factor (b)<br>Table Y<br><br>**End report-status**<br>Final pregnancy outcome (n)<br><br>If pregnancy ended $\leq 19^{+6}$, date (c) |

For peer review only

| | | | |
|---|---|---|---|
| Clinical indication for Macrosomia (b)<br>Amniotic fluid index (AFI) (c)<br>Single deepest pocket (c)<br><br>*Umbilical dopplers*<br>Gestational age (c)<br>Umbilical RI (resistive index) (n)<br>Umbilical PI (pulsatility index) (n)<br>Umbilical vein (n)<br><br>*Uterine dopplers*<br>L Ut PI (n)<br>R Ut PI (n)<br>Mean Ut PI (n)<br>L notch (n)<br>R notch (n)<br>Foetal outcome (n) | | | Gestational age at pregnancy end (c)<br><br>**MRI brain 1 year postpartum**<br>Blood pressure (c)<br>Pulse (c)<br>Plasma sample taken (b) |

For peer review only

STROBE Statement—Checklist of items that should be included in reports of ***cohort studies***

| | Item No | Recommendation |
|---|---|---|
| **Title and abstract** | 1 | (*a*) Indicate the study's design with a commonly used term in the title or the abstract<br>Title page |
| | | (*b*) Provide in the abstract an informative and balanced summary of what was done and what was found<br>Page 3-4 |
| **Introduction** | | |
| Background/rationale | 2 | Explain the scientific background and rationale for the investigation being reported<br>Page 6-7 |
| Objectives | 3 | State specific objectives, including any prespecified hypotheses<br>Page 7-8 |
| **Methods** | | |
| Study design | 4 | Present key elements of study design early in the paper<br>Page 7 |
| Setting | 5 | Describe the setting, locations, and relevant dates, including periods of recruitment, exposure, follow-up, and data collection<br>Page 7, 9-14 |
| Participants | 6 | (*a*) Give the eligibility criteria, and the sources and methods of selection of participants. Describe methods of follow-up<br>Page 9 and Figure 1 |
| | | (*b*) For matched studies, give matching criteria and number of exposed and unexposed<br>N/A |
| Variables | 7 | Clearly define all outcomes, exposures, predictors, potential confounders, and effect modifiers. Give diagnostic criteria, if applicable<br>Page 8. The study is a biobank and database that will also address future research questions and outcomes. |
| Data sources/ measurement | 8* | For each variable of interest, give sources of data and details of methods of assessment (measurement). Describe comparability of assessment methods if there is more than one group<br>Page 9-14 |
| Bias | 9 | Describe any efforts to address potential sources of bias<br>Page 15 |
| Study size | 10 | Explain how the study size was arrived at<br>Page 15-16 |
| Quantitative variables | 11 | Explain how quantitative variables were handled in the analyses. If applicable, describe which groupings were chosen and why<br>N/A, study protocol |
| Statistical methods | 12 | (*a*) Describe all statistical methods, including those used to control for confounding<br>Page 15 |
| | | (*b*) Describe any methods used to examine subgroups and interactions<br>N/A |
| | | (*c*) Explain how missing data were addressed<br>N/A, study protocol |
| | | (*d*) If applicable, explain how loss to follow-up was addressed<br>N/A, study protocol |
| | | (*e*) Describe any sensitivity analyses |


For peer review only - http://bmjopen.bmj.com/site/about/guidelines.xhtml

| | | |
|---|---|---|
| | | N/A |
| **Results** | | |
| Participants | 13* | (a) Report numbers of individuals at each stage of study—eg numbers potentially eligible, examined for eligibility, confirmed eligible, included in the study, completing follow-up, and analysed<br>N/A, study protocol |
| | | (b) Give reasons for non-participation at each stage<br>N/A, study protocol |
| | | (c) Consider use of a flow diagram<br>N/A, study protocol |
| Descriptive data | 14* | (a) Give characteristics of study participants (eg demographic, clinical, social) and information on exposures and potential confounders<br>N/A, study protocol |
| | | (b) Indicate number of participants with missing data for each variable of interest<br>N/A, study protocol |
| | | (c) Summarise follow-up time (eg, average and total amount)<br>N/A, study protocol |
| Outcome data | 15* | Report numbers of outcome events or summary measures over time<br>N/A, study protocol |
| Main results | 16 | (*a*) Give unadjusted estimates and, if applicable, confounder-adjusted estimates and their precision (eg, 95% confidence interval). Make clear which confounders were adjusted for and why they were included<br>N/A, study protocol |
| | | (*b*) Report category boundaries when continuous variables were categorized<br>N/A, study protocol |
| | | (*c*) If relevant, consider translating estimates of relative risk into absolute risk for a meaningful time period<br>N/A, study protocol |
| Other analyses | 17 | Report other analyses done—eg analyses of subgroups and interactions, and sensitivity analyses<br>N/A, study protocol |
| **Discussion** | | |
| Key results | 18 | Summarise key results with reference to study objectives<br>N/A, study protocol |
| Limitations | 19 | Discuss limitations of the study, taking into account sources of potential bias or imprecision. Discuss both direction and magnitude of any potential bias<br>Page 16-18 |
| Interpretation | 20 | Give a cautious overall interpretation of results considering objectives, limitations, multiplicity of analyses, results from similar studies, and other relevant evidence<br>N/A, study protocol |
| Generalisability | 21 | Discuss the generalisability (external validity) of the study results<br>N/A, study protocol |
| **Other information** | | |
| Funding | 22 | Give the source of funding and the role of the funders for the present study and, if applicable, for the original study on which the present article is based<br>Page 28 |

*Give information separately for exposed and unexposed groups.

For peer review only

**Note:** An Explanation and Elaboration article discusses each checklist item and gives methodological background and published examples of transparent reporting. The STROBE checklist is best used in conjunction with this article (freely available on the Web sites of PLoS Medicine at http://www.plosmedicine.org/, Annals of Internal Medicine at http://www.annals.org/, and Epidemiology at http://www.epidem.com/). Information on the STROBE Initiative is available at http://www.strobe-statement.org.

For peer review only



For peer review only - http://bmjopen.bmj.com/site/about/guidelines.xhtml

BMJ Open

# Study protocol; Establishment of a multi-center preeclampsia database and biobank in Sweden – GO PROVE and UP MOST, a prospective cohort study


| | |
|---|---|
| Journal: | *BMJ Open* |
| Manuscript ID | bmjopen-2021-049559.R2 |
| Article Type: | Protocol |
| Date Submitted by the Author: | 16-Oct-2021 |


| | |
|---|---|
| Complete List of Authors: | Thorgeirsdottir, Lilja; Sahlgrenska Academy, 1Department of Reproductive and Perinatal health, Institute of Health and Care Sciences<br>Andersson, Malin; Sahlgrenska Academy, Institute of clinical sciences, department of Anesthesiology and Intensive Care<br>Karlsson, Ove ; Sahlgrenska Academy, Department of Anesthesiology and Intensive Care, Institute of clinical sciences<br>Thörn, Sven-Egron; Institute of Clinical Sciences at Sahlgrenska Academy, University of Gothenburg, Department of Anaesthesiology and Intensive Care Medicine<br>Oras, Jonatan; Sahlgrenska Academy, 3Department of Anesthesiology and Intensive Care, Institute of clinical sciences<br>Sengpiel, Verena; University of Gothenburg Sahlgrenska Academy, Department of obstetrics and gynecology; Sahlgrenska Universitetssjukhuset Östra sjukhuset, Department of obstetrics and gynecology<br>Svanvik, Teresia; University of Gothenburg Sahlgrenska Academy, Department of obstetrics and gynecology<br>Elden, Helen; Goteborgs Universitet, Health and Care Sciences<br>Linden, Karolina; Goteborgs universitet Institutionen for vardvetenskap och halsa,<br>Junus, Katja; Uppsala University, Department of women's and children's health<br>Lager, Susanne; Uppsala University, Women's and Children's Health; Uppsala University, Centre for Reproductive Biology in Uppsala (CRU)<br>Enskär, Ida; Uppsala University, Department of women's and children's health<br>van Veen, Teelkien; University Medical Centre Groningen, Department of obstetrics and gynecology<br>Wikström, Johan; Department of Surgical Sciences, Radiology Unit, Department of Surgical Sciences, Radiology Unit<br>Björkman-Burtscher, Isabella; Göteborgs universitet Institutionen för kliniska vetenskaper, Radiology<br>Stigsdotter Neely, Anna; Karlstad University, Department of Social and Psychological Studies<br>Wikström, Anna-Karin; Karolinska institutet, Department of Medicine Unit of clinical epid; Uppsala University, Womens and Childrens Health<br>Bergman, Lina ; Sahlgrenska Academy, Institute of clinical sciences, department of obstetrics and gynecology; Uppsala University, Department of women's and children's health |

| | |
|---|---|
| <b>Primary Subject Heading</b>: | Reproductive medicine |
| Secondary Subject Heading: | Obstetrics and gynaecology |
| Keywords: | Maternal medicine < OBSTETRICS, RADIOLOGY & IMAGING, ULTRASONOGRAPHY, NEUROLOGY, QUALITATIVE RESEARCH |
| | |


SCHOLARONE™ Manuscripts

*I, the Submitting Author has the right to grant and does grant on behalf of all authors of the Work (as defined in the below author licence), an exclusive licence and/or a non-exclusive licence for contributions from authors who are: i) UK Crown employees; ii) where BMJ has agreed a CC-BY licence shall apply, and/or iii) in accordance with the terms applicable for US Federal Government officers or employees acting as part of their official duties; on a worldwide, perpetual, irrevocable, royalty-free basis to BMJ Publishing Group Ltd ("BMJ") its licensees and where the relevant Journal is co-owned by BMJ to the co-owners of the Journal, to publish the Work in this journal and any other BMJ products and to exploit all rights, as set out in our licence.*

*The Submitting Author accepts and understands that any supply made under these terms is made by BMJ to the Submitting Author unless you are acting as an employee on behalf of your employer or a postgraduate student of an affiliated institution which is paying any applicable article publishing charge ("APC") for Open Access articles. Where the Submitting Author wishes to make the Work available on an Open Access basis (and intends to pay the relevant APC), the terms of reuse of such Open Access shall be governed by a Creative Commons licence – details of these licences and which Creative Commons licence will apply to this Work are set out in our licence referred to above.*

*Other than as permitted in any relevant BMJ Author's Self Archiving Policies, I confirm this Work has not been accepted for publication elsewhere, is not being considered for publication elsewhere and does not duplicate material already published. I confirm all authors consent to publication of this Work and authorise the granting of this licence.*

For peer review only

# Study protocol; Establishment of a multi-center preeclampsia database and biobank in Sweden – GO PROVE and UP MOST, a prospective cohort study

Lilja Thorgeirsdottir[1,2], Malin Andersson[3], Ove Karlsson[3], Sven-Egron Thörn[3], Jonatan Oras[3], Verena Sengpiel[2,4], Teresia Svanvik[2,4], Helen Elden[1,2], Karolina Linden[1], Katja Junus[5], Susanne Lager[5], Ida Enskär[5], Teelkien van Veen[6], Johan Wikström[7], Isabella M. Björkman-Burtscher[8], Anna Stigsdotter Neely[8,9], Anna Karin Wikström[5], Lina Bergman[2,4,11]

[1]Institute of Health and Care Sciences, Sahlgrenska Academy, University of Gothenburg, Gothenburg, Sweden

[2]Department of Obstetrics and Gynecology, Sahlgrenska University Hospital, Gothenburg, Sweden

[3]Department of Anesthesiology and Intensive Care, Institute of clinical sciences, Sahlgrenska Academy, University of Gothenburg, Gothenburg, Sweden

[4]Region Västra Götaland, Sahlgrenska University Hospital, Department of obstetrics and gynecology, Gothenburg, Sweden

Department of Obstetrics and Gynecology, Institute of clinical sciences, Sahlgrenska Academy, University of Gothenburg, Gothenburg, Sweden

[5]Department of Women's and Children's Health, Uppsala University, Uppsala, Sweden

[6]Department of Obstetrics and Gynecology, University Medical Center Groningen, Groningen, the Netherlands

[7]Department of Surgical Sciences, Radiology, Uppsala University

[8]Department of Radiology, Institute of Clinical Sciences, Sahlgrenska Academy, University of Gothenburg and Sahlgrenska University Hospital, Gothenburg, Sweden

[8]Department of Social and Psychological Studies, Karlstad University, Sweden

[9]Engineering Psychology Luleå University of Technology

[11]Department of Obstetrics and Gynecology, Stellenbosch University, Cape Town, South Africa

Corresponding author

Lina Bergman

Department of Obstetrics and Gynecology

PO Kvinnokliniken SU Östra

SE 416 85 Göteborg



For peer review only - http://bmjopen.bmj.com/site/about/guidelines.xhtml

Sweden

Telephone number 0046707920780

Email: lina.bergman.2@gu.se

Word count: 3561

For peer review only



For peer review only - http://bmjopen.bmj.com/site/about/guidelines.xhtml

## ABSTRACT

### Introduction

Preeclampsia, a multi-system disorder in pregnancy, is one of the most common causes of maternal morbidity and mortality worldwide. However, we lack methods for objective assessment of organ function in preeclampsia and predictors of organ impairment during and after preeclampsia. The women's and their partners' experiences of preeclampsia have not been studied in detail. To phenotype different subtypes of the disorder is of importance for prediction, prevention, surveillance, treatment and follow-up of preeclampsia.

The aim of this study is to set up a multi-center database and biobank for preeclampsia in order to contribute to a safer and more individualized treatment and care.

### Methods and analysis

This is a multicenter cohort study. Prospectively recruited pregnant women ≥18 years, diagnosed with preeclampsia presenting at Sahlgrenska University Hospital, Uppsala University Hospital and at Södra Älvsborgs Hospital, Sweden, as well as normotensive controls are eligible for participation. At inclusion and at one-year follow-up, the participants donate bio samples that are stored in a biobank and they are also asked to participate in various organ specific evaluations. In addition, questionnaires and interviews regarding the women's and partner's experiences are distributed at follow up.

### Ethics and dissemination

By creating a data- and biobank, we will provide the means to explore the disorder in a broader sense and allow clinical and laboratory discoveries that can be translated to clinical trials aiming at improved care of women with preeclampsia. Further, to evaluate experiences and the psychological impact of being affected by preeclampsia can improve the care of pregnant women and their partners. In case of incidental pathological findings during examinations performed, they will be handled in accordance with clinical routine. Data is




For peer review only - http://bmjopen.bmj.com/site/about/guidelines.xhtml

stored in a secure online database. Biobank samples are identified through the women's personal identification number and pseudonymised after identification in the biobank before analysis.

This study was approved by the regional ethical review board in Gothenburg on the 28th December 2018 (approval number 955-18) and by the Swedish Ethical Review Authority on the 27th of February 2019 (approval number 2019-00309).

Results from the study will be published in international peer-reviewed journals.

**Trial Registration**

Trial ID: ISRCTN13060768

Date registered: 27/07/2020

Link: https://www.isrctn.com/ISRCTN13060768

**Keywords**: (3-10 max) Biobank, brain, cerebral autoregulation, cognition, partner experience, RH-PAT, UCG, MRI

**Strengths and limitations of this study**

- This study is a national three center study, enabling inclusion of women from different socioeconomic backgrounds and by the size of the units, permits a large biobank and database of preeclampsia in a short time frame.
- The interdisciplinary team of obstetricians, midwives, anaesthesiologists, radiologists and cardiologists and collaboration between units from developed and developing countries contribute to a wider understanding of the disease from different aspects.
- The study uses pre-defined variables by an international consensus of predictors and outcomes in preeclampsia research – enabling merging of data with other international research groups.

For peer review only



For peer review only - http://bmjopen.bmj.com/site/about/guidelines.xhtml

- The hospital integrated biobanks ensure a high quality and standardized methods for the biological samples included in the study.
- Women with preeclampsia are delivered (as a cure) often before onset of complications and thus the endpoints will often consist of "preterm delivery" instead of true maternal organ complications.

For peer review only



For peer review only - http://bmjopen.bmj.com/site/about/guidelines.xhtml

## INTRODUCTION

Preeclampsia is one of the most serious complications of pregnancy affecting 3-8 % of pregnancies worldwide.[1,2] Preeclampsia leads to more than 60,000 maternal deaths annually and is also a common cause of maternal and neonatal morbidity.[2,3] Preeclampsia is a multi-organ disorder involving the brain, cardiovascular system, fetoplacental unit, hematological system, kidneys, liver, lungs and maternal vessels.[1] It may cause heart failure, pulmonary edema, renal failure, hemostatic disorders, cerebrovascular events, seizures (eclampsia), intrauterine growth restriction and intrauterine death. The pathophysiology of preeclampsia is not completely understood, but the hypothesis is based on placental dysfunction, in particular in early onset disease (diagnosis <34 gestational weeks). In late onset disease, maternal factors such as diabetes, cardiac disease, multi-fetal pregnancy and obesity are thought to play a more important role.[4] There is a paucity of data on how to evaluate threatening organ dysfunction in preeclampsia. At present, there are only a few tests available mainly regarding liver-, coagulation- and renal function.[5] Clinicians lack predictors or objective assessment of cardiovascular, endothelial, or neurological dysfunction in preeclampsia. An increased understanding of the underlying pathophysiology of organ complications and increased accuracy regarding prediction would allow individualized treatment. This includes magnesium sulphate for prevention of seizures, targeted blood pressure treatment for prevention of intracranial hemorrhage, customized administration of regional anesthesia, particularly in cases with disturbed coagulation and targeted treatment in case of cardiac dysfunction. By identifying biomarkers with high sensitivity and specificity for various threatening organ complications, end organ complications could be identified and managed in time and some cases of iatrogenic preterm labor could be avoided, potentially improving the short- and long-term outcomes for babies born to women with early onset preeclampsia.[6]

For peer review only



For peer review only - http://bmjopen.bmj.com/site/about/guidelines.xhtml

The capacity to recognize, exploit and phenotype different subtypes of preeclampsia is of importance for prediction, prevention, surveillance, treatment and follow-up of preeclampsia. This protocol adheres to the international consensus about standardized data and biological sample collections in preeclampsia research.[7] Adherence to such standardized protocols will facilitate the cooperation with other research teams and hopefully improve the understanding of the causes of preeclampsia and development of targeted treatment strategies.

The women's and their partners' experiences of preeclampsia during pregnancy and postpartum has not been studied in detail. Qualitative interview studies with women with preeclampsia have described preeclampsia as a fearful, powerless, painful and/or life-threatening event. They have also described inadequate clinical management and care and many women felt lucky to have survived.[8,9] By studying how women and their partners experience preeclampsia and the consequences regarding health-related quality of life, self-efficacy, anxiety, childbirth experiences and breastfeeding, we aim to improve personalized care and follow-up after a pregnancy complicated by preeclampsia.

## METHODS/DESIGN

This is a Swedish multicenter prospective cohort study prospectively including women diagnosed with preeclampsia and their partners and in addition normotensive controls. A flow chart of the study is presented in Figure 1. The study started to include women during the fall of 2019 and will continue to recruit until 2023.

### Aims

The overall aim of this study is to establish a database and biobank including two Swedish University preeclampsia cohorts, GO PROVE (Gothenburg Preeclampsia Obstetric adverse events) and UP MOST (Uppsala Pregnancy Complication Study) for preeclampsia research, to facilitate laboratory-based, translational and clinical research on preeclampsia and to



For peer review only - http://bmjopen.bmj.com/site/about/guidelines.xhtml

contribute to a safer and more patient centered care of women with preeclampsia in the acute and long-term perspective, focused on specific organ involvement. Thus, the following actions are included in the project:

- To establish a biobank and database with core predictors and outcomes for preeclampsia research in Sweden (GO PROVE and UP MOST)
- To examine alterations in cerebral structure and function through magnetic resonance (MR) investigation of the brain, doppler examination of cerebral blood flow, cerebral biomarkers, electroencephalograms (EEG) and neurocognitive assessment of mental processing speed, working memory, executive functions, and episodic memory in women with preeclampsia compared to normotensive controls at diagnosis and at one year postpartum (GO PROVE and UP MOST)
- To examine cardiac impairment and prediction of cardiac impairment through echocardiography and cardiac biomarkers in women with preeclampsia compared to normotensive controls at diagnosis and at one year postpartum (GO PROVE)
- To examine vascular dysfunction through measurement of peripheral resistance and blood-based endothelial biomarkers in women with preeclampsia compared to normotensive controls at diagnosis and at one year postpartum (GO PROVE)
- To examine coagulation profiles through blood-based biomarkers and tests such as thromboelastometry (ROTEM®) and impedance aggregometry (Multiplate®) in women with preeclampsia compared to normotensive controls (GO PROVE) To increase the knowledge and understanding of women's and their partners' experiences of preeclampsia during pregnancy, birth and postpartum through validated measurements and qualitative interviews (GO PROVE)

**Population**

For peer review only

162 Pregnant women ≥18 years of age with a diagnosis of preeclampsia after gestational week 20, presenting at Sahlgrenska University Hospital (10 500 deliveries/year), Uppsala University Hospital (4 200 deliveries/year) and Södra Älvsborgs Hospital (3 100 deliveries/year) are approached for inclusion. Both participating women and their partner are eligible for the qualitative interview study. Healthy women ≥18 years with normotensive pregnancies are included as controls.

168 Exclusion criteria: Women unable to understand oral and written information and unable to give written informed consent are not eligible as well as women with pre-existing hypertension, diabetes mellitus before or during pregnancy and pre-existing cardiovascular, renal or cerebral disease. MR contraindications for MR group includes claustrophobia. Women who have previously been included in the study will not be eligible for their next pregnancy.

174 For the database and biobank as a whole there will be no matching. For specific sub-studies there might be matching on a group level for gestational age at examination.

176 The diagnosis of preeclampsia will be established according to Swedish guidelines for hypertensive disorders in pregnancy (www.sfog.se) that generally follows international guidelines such as ISSHP, NICE and ACOG with the addition that we will require proteinuria to be present to limit the heterogeneity of the population.

180 **GO PROVE and UP MOST database**

181 The database is provided by MedSciNet (MedSciNet AB, Stockholm, Sweden) and includes exposure and outcome variables as proposed for preeclampsia research by the international collaborative group for preeclampsia research Co-Lab.[7] In addition, specific variables for this study have been included (Table S1 and Table 1). Biochemical tests when performed in routine health care are also collected, in addition to biochemical tests analyzed specifically for the study, and are presented in Table 2.



For peer review only - http://bmjopen.bmj.com/site/about/guidelines.xhtml

**GO PROVE and UP MOST Biobank**

GO PROVE and UP MOST use the Hospital Integrated Biobank (SIB, www.biobanksverige.se) that enables samples to be handled in a standardized manner through the hospitals' clinical laboratories, thus ensuring sample quality. Samples are sent to the local hospital's clinical chemistry laboratory or directly to the hospital biobank facility, where they are spun, aliquoted into 225 µl wells and frozen within four to six hours. Samples are only thawed directly prior to analysis. Each new project utilizing the biobank requires ethical approval, but no additional informed consent. At inclusion in the study, at delivery and at one year follow up, maternal blood and urine are collected. At delivery, placental tissue, umbilical cord blood sample and cerebrospinal fluid are collected in addition to above. Cerebrospinal fluid is only collected if spinal anasthesia is administered. If the woman undergoes MRI brain, an extra blood sample is drawn. A detailed description of samples can be found in Table 3. By merging the participants in the biobank with the GO PROVE and UP MOST database, a data- and biobank is created that will also include results from organ-specific evaluation as described below.

Bio-samples will be analyzed for pre-specified biomarkers such as cerebral biomarkers, angiogenic biomarkers, cardiac biomarkers and endothelial biomarkers. In addition, bio-samples will be available for future analyses of other potential biomarkers within the field of preeclampsia by the authors and other research groups after ethical approval and approval from the research group.

**GO PROVE and UPMOST examinations**

For an overview of included examinations, please see Figure 1.

*Magnetic resonance (MR) examination of the brain, assessment of cerebral autoregulation and cognitive function scoring*

<u>MR brain</u>

For peer review only

212 Investigations will be performed using 3T MR scanners at inclusion and at one year postpartum for cases and controls. Imaging protocols contain the following sequences: 3D T1-weighted (w) MPRAGE sequence, 3D fluid attenuated inversion recovery (FLAIR), and 2D diffusion weighted imaging (DWI) with three directions and multiple b-values for morphological evaluation of e.g. edema and gross pathology as well as perfusion assessment using intravoxel incoherent motion (IVIM) technique,[10] 2D DWI with 32 directions to evaluate white matter microstructure,[11] arterial spin labeling (ASL) for perfusion assessment,[10,12] 3D time of flight (TOF) MR angiography for vessel evaluation, susceptibility weighted imaging (SWI/QSM or SWAN) for assessment of hemorrhage and resting state functional MR (rsfMR) for evaluation of neuronal networks.[13]

222 Neurocognitive assessment

223 To determine whether the participating women experience cognitive impairment, several instruments that measure, both subjective and objective cognition, provide a fine-grained picture of the participants' cognitive performance. Subjective cognitive function is assessed by the Cognitive Failure Questionnaire (CFQ) measuring self-reported cognitive failures in everyday life.[14] The objective cognitive test battery consists of 11 tests covering a broad range of cognitive abilities. Verbal ability is assessed with a Swedish four-alternative multiple-choice synonym test (SRB 1) and serves as an estimate of premorbid cognitive ability.[15] Mental speed is measured by Digit Symbol Coding from Wechsler Adult Intelligence Scale (WAIS IV).[16] Working memory is assessed by the letter-number sequencing task and the three digit span tasks forward, backward and sequencing from WAIS-IV.[16] Executive functions are measured by three tasks from the Delis–Kaplan Executive Function System (D-KEFS) (a) Mental shifting/flexibility with the Trail Making Test; (b) Inhibition with the Stroop test; and (c) Verbal fluency with Letter and Category fluency test.[17] Several aspects of episodic memory performance are measured with free and



For peer review only - http://bmjopen.bmj.com/site/about/guidelines.xhtml

delayed recall of nouns using the Buschke Selective Reminding procedure and prospective episodic memory with the Reminding task.[18,19] All cognitive tests have good psychometric properties. The tests are administered by study personnel, at inclusion at the hospital and again at one year postpartum.

<u>Transcranial cerebral doppler</u>

Measurement of the blood flow velocity in the middle cerebral artery (MCA) is performed with transcranial doppler technique and combined with continuous non-invasive measurement of the blood pressure using finger arterial volume clamping (calibrated with brachial blood pressure measurement) and carbon dioxide concentrations in exhaled air. The signal is obtained through the temporal window and systolic, mean and diastolic blood flow velocity and depth of measurement are registered. The measurements are performed by trained investigators and intra- and inter-observer variation is measured and kept below 15%. Cerebral autoregulation is determined from the response of the cerebral blood flow velocity to spontaneous fluctuations in mean blood pressure as described previously and reported as Autoregulation Index.[20] The measurements will be compared between cases and normotensive controls and between inclusion and at one year postpartum.

*Echocardiography and endothelial dysfunction measurement*

<u>Echocardiography</u>

Women with preeclampsia and pregnant controls are examined with transthoracic echocardiography (TTE) for evaluation of systolic and diastolic function. Systolic dysfunction is defined as having an ejection fraction less than 50% or having regional hypokinesia. Diastolic dysfunction is defined as fulfilling at least 50% of the following; an average peak velocity of early diastolic transmitral flow / peak velocity of early diastolic mitral annular motion as determined by pulsed wave Doppler (E/e´) >14, septal e´ velocity < 7cm/s or lateral e´ velocity < 10cm/s, tricuspid regurgitation velocity > 2.8m/s or left atrial



For peer review only - http://bmjopen.bmj.com/site/about/guidelines.xhtml

volume index $>34 ml/m^2$[21]. Right ventricular function is assessed by eye-balling, tricuspid Annular Plane Systolic Excursion (TAPSE) and right ventricular peak velocity of systolic mitral annular motion as determined by pulsed wave Doppler (RV S′).

The examination is performed in proximity of inclusion and one year postpartum.

<u>Peripheral artery resistance measurements</u>

The status of the peripheral vessels can be measured with a variety of methods. In common, they are based on the interpretation of pulse pressure curve in the vessels. In this study we use the Reactive Hyperemia-Peripheral Arterial Tonometry (RH-PAT) method. The RH-PAT induces ischemia in one arm during 5 minutes. The pulse pressure wave in the peripheral artery, i.e. the finger, is measured before, during and after ischemia in both arms. The difference in reaction is recorded and reflects the vascular status peripherally.[22]

The Systemic Vascular Resistance index (SVRI) is calculated from the cardiac output and estimation of central venous pressure (CVP) measured through TTE indexed to body mass. SVRI reflects the afterload i.e. the workload that the heart has to handle at a normovolemic state which in turn will be affected by the patient's blood pressure and peripheral vasoconstriction.[23]

The measurements of the heart function and the derived Systemic Vascular resistance retrieved from the TTE will then be compared with the measurement of peripheral vascular function as measured by the RH-PAT method and serum biomarkers clinically used to measure heart failure. The examination is performed at inclusion and again at one year postpartum.

*Hemostasis*

Hemostatic status can be assessed by laboratory analyses and by point-of-care devices as thromboelastometry (ROTEM®) and impedance aggregometry (Multiplate®). ROTEM® is an established viscoelastic method for hemostasis testing in whole blood and a modification of

For peer review only



For peer review only - http://bmjopen.bmj.com/site/about/guidelines.xhtml

traditional thromboelastography (TEG®). ROTEM® investigates the interaction of coagulation factors, their inhibitors, anticoagulant drugs, blood cells (specifically platelets) during clotting and subsequent fibrinolysis.[24] Multiplate® measures platelet function through impedance aggregometry.[25] ROTEM® and Multiplate® are assessed at inclusion and again at delivery (GO-PROVE).

**Women's and partner's experiences of preeclampsia during pregnancy, childbirth and postnatal care**

*Questionnaires*

Effects of preeclampsia on women's self-reported health are measured with validated instruments sent to them by email, 8-12 weeks postpartum: Self-efficacy, the belief that one is capable of accomplishing a behavior or developing a competency to succeed in reaching a specific or more general goal, is measured by the General Self-Efficacy scale (GES).[26] Anxiety and depression are measured by Hospital anxiety and depression scale (HADS)[27] and Edinburgh postnatal depression scale (EPDS).[28] Health-related quality of life is determined by the EQ-visual analog scale (EQ- VAS).[29] Childbirth experiences is measured by the Childbirth Experience Questionnaire (CEQ)[30] and levels of breastfeeding self-efficacy is assessed through the Breastfeeding Self-Efficacy Scale short form (BES).[31] Post-Traumatic Stress Symptoms is evaluated by the Post traumatic stress disorder (PTSD) Checklist, DSM-5 (PCL-5).[32]

*Interviews*

Experiences of preeclampsia during pregnancy, childbirth and postnatal care are described through qualitative interviews. Informants will be 15-20 women who have experienced preeclampsia with severe features during pregnancy and their partners.[33] Informants are selected to ensure a broad range of views and experiences of the phenomenon preeclampsia. Face-to-face interviews[34,35] are conducted 2 to 3 months after delivery. An open–ended

For peer review only



For peer review only - http://bmjopen.bmj.com/site/about/guidelines.xhtml

question is used, “Please tell me of your experience of preeclampsia” or “please tell me of your experience as a partner to a woman with preeclampsia”.

**Data processing, analysis and statistics**

Results from cerebral doppler, echocardiography, and RH-PAT estimations will be calculated by two independent interpreters blinded to groups and entered manually into the database. MR data will be analyzed in collaboration by neuroradiologists, blinded to groups. Blood samples will be thawed for analyses of cardiac-, renal-, neurological- and endothelial biomarkers, analyzed through standardized platforms when available and for the remaining through manual ELISA analyses in duplicates. Inter- and intra-assay coefficients of variation will be aimed at below 10%.

Demographics will be presented as medians or means as appropriate by distribution and by numbers and percentages as appropriate. Comparison between groups will be analyzed by student’s t-test or Mann Whitney U-test with means or medians and confidence intervals or interquartile range, as appropriate according to distribution of the variables. Proportions will be analyzed through Chi Square test. Correlations will be analyzed by Pearson’s r or Spearman’s rho as appropriate by distribution of the variable. Regression analyses, unadjusted and adjusted, will be performed to adjust for known confounding variables. Ten cases per variable at the lowest will be considered appropriate to avoid overfitting of the model. All statistical analyses will be performed in SPSS or R. Analyzes of cognitive function will be performed through ANOVA AND MANOVA.

Data analysis of the interview data will be conducted by phenomenology with a lifeworld approach[36] or with qualitative content analysis.[37] NVivo 10 software (qsrinternational.com/) is used for grouping and organizing the text.

**Power calculations**



For peer review only - http://bmjopen.bmj.com/site/about/guidelines.xhtml

Uppsala and Sahlgrenska University hospitals have 4,200 and 10,500 deliveries yearly respectively and Södra Älvsborgs Hospital 3,100 deliveries yearly. Preeclampsia affects 3% of the population resulting in approximately 530 cases yearly at the centers and approximately 410 cases after exclusions according to the study protocol. For logistical reasons, all women cannot be included. Of eligible, we estimate that we will recruit around 20% for MR, transcranial doppler and cognition examinations (70/year) at Uppsala and Sahlgrenska University hospitals and about 40/year at Sahlgrenska University hospital for cardiac and endothelial function tests. Therefore, we estimate that inclusion will take two years. Inclusion during two years at two centers will thus give around 140 MR investigations, 100 transcranial doppler and cognition examinations and 40 TTE. We will include 60 controls for each investigation. For blood based tests we will use the whole cohort. We want to include more cases than controls since we want to be able to subtype cases based on preeclampsia phenotypes. Some specific power calculations:

*Cerebral blood flow regulation and tissue perfusion:* in order to demonstrate a change of 3 ml / min x 100g in perfusion in the cortical sub-volume of the brain,[38] 44 cases of preeclampsia and 44 normotensive controls with healthy pregnancies are required. In order to detect a difference in dynamic cerebral autoregulation index of 1.2,[20] a sample size of 32 women in each group is estimated to be required. In addition, differences in cerebral perfusion and dynamic cerebral autoregulation have been detected in the above sample sizes of 44 and 20 preeclampsia cases respectively with an equal number of normotensive controls.

*Cognition:* In a meta-analysis, a difference in the objective test “letter number sequencing task” was found when pooling results from 101 women with previous preeclampsia and 98 women with previous healthy pregnancies.[39]

**Patient and Public Involvement**



For peer review only - http://bmjopen.bmj.com/site/about/guidelines.xhtml

To ensure that the research is relevant, acceptable and feasible from the women's and partners' point of view patient representatives were invited to participate in the research process.[40] Focusing on women's and their partners' experiences of preeclampsia diagnosis and care at the same time will ensure that findings from this project will improve not only clinical outcome measures but also women's and their partners' experience.

## ETHICS AND DISSEMINATION

This is a cohort study with a control group with no intervention for the participants. Incidental pathological findings on examinations performed are and will be handled in accordance with clinical routine. Data is stored in a secure online database. Biobank samples are identified through the women's personal identification number and pseudonymised after identification in the biobank before analysis. Results from the study will be published in international peer-reviewed journals.

## DISCUSSION

There is a need for more individualized treatment of women with preeclampsia with respect to different organ manifestations of the disease. This in order to individualize treatment and care, such as an informed decision to deliver and to avoid short- and long-term complications for the mother and infant. Currently there are no reliable methods in clinical practice to evaluate organ dysfunction such as cardiovascular and cerebral effects of preeclampsia, although cerebral edema, intracranial bleeding and pulmonary edema secondary to heart failure are the most common causes of maternal mortality due to preeclampsia. There are emerging biomarkers and prediction models but none of them are used in routine care yet and they do not seem to be able to differentiate between different organ dysfunction.[41,42]



For peer review only - http://bmjopen.bmj.com/site/about/guidelines.xhtml

The interdisciplinary team of obstetricians, midwives, anesthesiologists, neuroradiologists and cardiologists enables us to link organ specialists in the field of preeclampsia research. This collaboration will enable important discoveries that have great potential to improve the care of these women. In addition, by employing a database created to harmonize data collection in preeclampsia research, biological samples and data can be shared with other research groups, enabling larger datasets for rare outcomes.[7] Prospective recruitment may imply selection bias. In this study, inclusions will mainly occur daytime by research personnel even though medical staff have the opportunity to include women after office hours (between 4 p.m and 8 a.m.). This might introduce selection bias regarding severity of disease where women with sudden onset of organ complications are more often seen and delivered after office hours. In addition, there might be a larger proportion of women with early onset preeclampsia recruited since women with late onset preeclampsia are induced shortly after diagnosis. However, national health records are available to compare demographics of the study population to the general population with preeclampsia.

Regarding cerebral dysfunction in preeclampsia, we will be able to further characterize imaging abnormalities, circulating cerebral biomarkers and cognitive function among women with preeclampsia and neurological impairment. Some women with preeclampsia show evidence of cerebral edema in the acute phase[43,44] and long-term follow-up of women with previous preeclampsia demonstrate an increased number of white matter lesions.[45] There is a paucity in data regarding the connection between acute lesions and long-term effects on the brain and their implications in the development of stroke, epilepsy, and dementia. Cerebral biomarkers have been reported to be increased in preeclampsia but the connection to objective findings on MR or cognitive function is not clear.[46,47] Hopefully, data from this study can enable an objective assessment of neurological function in preeclampsia as a tool for targeting women at risk for adverse cerebral events. In addition, the long-term cerebral



For peer review only - http://bmjopen.bmj.com/site/about/guidelines.xhtml

effects of preeclampsia are still an area that needs more research. There are reports of an increased risk of dementia, stroke and epilepsy after a pregnancy complicated by preeclampsia but the causality and importance of preeclampsia as an insult to the brain is not fully elucidated.[48-50] Cognitive impairment years after a pregnancy complicated by preeclampsia has been reported but it is not known whether this cognitive impairment exists already at diagnosis and how it changes over the first year postpartum after preeclampsia.[51,52] Findings from this study might increase the understanding of the underlying pathophysiological mechanisms of acute cerebral injury and long-term effects.

Regarding hemostasis, our results will hopefully provide guidance as to when women with preeclampsia can receive spinal anesthesia and further insight into the coagulation profile among women with severe preeclampsia.

For preeclampsia and cardiovascular function, an initial assessment of cardiovascular function with TTE and peripheral resistance measurement for assessment of endothelial dysfunction for all women in the study will aid in the risk-classification of women stratified on cardiovascular involvement.

Increased knowledge about how women as well as partners experience preeclampsia and the consequences could improve personalized care and follow-up after preeclampsia.

Preeclampsia is a disease only manifested in humans, hence there are no animal models of preeclampsia with the ability to exactly mimic the true preeclampsia state.[53] Therefore, it is imperative that critical laboratory and clinical observations are made on humans and human tissues. By creating a data- and biobank, we will be able to make important laboratory discoveries that can be translated to clinical trials that may improve the care of women.



For peer review only - http://bmjopen.bmj.com/site/about/guidelines.xhtml

## LIST OF ABBREVIATIONS

BES Breastfeeding Self-Efficacy Scale short form

CEQ Childbirth Experience Questionnaire

CFQ Cognitive Failures Questionnaire

D-KEFS Delis-Kaplan Executive Function System

DSM 5 (PCL-5) Post Traumatic Stress Disorder Checklist

DWI Diffusion weighted images

EPDS Edinburgh Postnatal Depression Scale

EQ-VAS Euro Qol Visual Analogue Scale

FLAIR Fluid attenuated inversion recovery

GES General Self-Efficacy Scale

GO-PROVE Gothenburg Obstetric Adverse Events

HADS Hospital Anxiety and Depression Scale

MCA Middle cerebral artery

MRA 3D TOF Magnetic resonance angiography 3 dimensional time of flight

MR Magnetic Resonance

PROVE Preeclampsia Obstetric Adverse Events

PTSD Post traumatic stress disorder

RH-PAT reactive hyperemia-peripheral arterial tonometry

ROTEM Rotational tromboelastometry

SIB Hospital integrated biobank

SU Sahlgrenska University Hospital

TEG Thromboelastography

UP-PROVE Uppsala Pregnancy Complication Study

WAIS IV Wechsler Adult Intelligence Scale, fourth version

For peer review only

## DECLARATIONS

### Ethics approval and consent to participate

This study was approved by the regional ethical review board in Gothenburg on the 28th December 2018 (approval number 955-18) and by the Swedish Ethical Review Authority on the 27th of February 2019 (approval number 2019-00309).

All participating women are given oral and written information and sign an informed consent before participation in the study.

### Consent for publication

Not applicable.

### Availability of data and materials

The datasets used and/or analyzed during the current study are available from the corresponding author on reasonable request.

### Acknowledgment

We would like to thank Biobank Väst and Uppsala Biobank (a part of SIB) for supporting with collection of biological specimens.

For peer review only



For peer review only - http://bmjopen.bmj.com/site/about/guidelines.xhtml

## REFERENCES

1. Mol BWJ, Roberts CT, Thangaratinam S, Magee LA, de Groot CJM, Hofmeyr GJ. Pre-eclampsia. *Lancet.* 2016;387(10022):999-1011.

2. Duley L. The global impact of pre-eclampsia and eclampsia. *Semin Perinatol.* 2009;33(3):130-137.

3. Lain KY, Roberts JM. Contemporary concepts of the pathogenesis and management of preeclampsia. *JAMA.* 2002;287(24):3183-3186.

4. von Dadelszen P, Magee LA, Roberts JM. Subclassification of preeclampsia. *Hypertension in pregnancy : official journal of the International Society for the Study of Hypertension in Pregnancy.* 2003;22(2):143-148.

5. Brown MA, Magee LA, Kenny LC, et al. The hypertensive disorders of pregnancy: ISSHP classification, diagnosis & management recommendations for international practice. *Pregnancy Hypertens.* 2018.

6. Sibai BM. Preeclampsia as a cause of preterm and late preterm (near-term) births. *Seminars in Perinatology.* 2006;30(1):16-19.

7. Myatt L, Redman CW, Staff AC, et al. Strategy for standardization of preeclampsia research study design. *Hypertension.* 2014;63(6):1293-1301.

8. Furuta M, Sandall J, Bick D. Women's perceptions and experiences of severe maternal morbidity--a synthesis of qualitative studies using a meta-ethnographic approach. *Midwifery.* 2014;30(2):158-169.

9. Vaerland IE, Vevatne K, Brinchmann BS. An Integrative Review of Mothers' Experiences of Preeclampsia. *J Obstet Gynecol Neonatal Nurs.* 2016;45(3):300-307.

10. Le Bihan D, Breton E, Lallemand D, Aubin ML, Vignaud J, Laval-Jeantet M. Separation of diffusion and perfusion in intravoxel incoherent motion MR imaging. *Radiology.* 1988;168(2):497-505.



For peer review only - http://bmjopen.bmj.com/site/about/guidelines.xhtml

11. Pierpaoli C, Jezzard P, Basser PJ, Barnett A, Di Chiro G. Diffusion tensor MR imaging of the human brain. *Radiology.* 1996;201(3):637-648.

12. Williams DS, Detre JA, Leigh JS, Koretsky AP. Magnetic resonance imaging of perfusion using spin inversion of arterial water. *Proc Natl Acad Sci U S A.* 1992;89(1):212-216.

13. Biswal B, Yetkin FZ, Haughton VM, Hyde JS. Functional connectivity in the motor cortex of resting human brain using echo-planar MRI. *Magn Reson Med.* 1995;34(4):537-541.

14. Broadbent DE, Cooper PF, FitzGerald P, Parkes KR. The Cognitive Failures Questionnaire (CFQ) and its correlates. *Br J Clin Psychol.* 1982;21 (Pt 1):1-16.

15. Dureman I KL, Österberg E. *Manual till DS-batteriet [Manual for the DS-battery].* Stockholm: Psykologiförlaget; 1971.

16. Wechsler D. *WAIS-IV Administration and Scoring Manual.* San Antonio, TX: The Psychological Corporation; 2008.

17. Delis DC Kaplan E KJ. *Delis-Kaplan Executive Functioning System (D-KEFS).* San Antonio: The Psychological Corporation; 2001.

18. Buschke H. Selective reminding for analysis of memory and learning. *J Verb Learn Verb Behav.* 1973;12:543–550.

19. Mäntylä T, Nilsson LG. Remembering to remember in adulthood: A population-based study on aging and prospective memory. *Journal of Neuropsyhol Cogn.* 1997;4:81-92.

20. van Veen TR, Panerai RB, Haeri S, Griffioen AC, Zeeman GG, Belfort MA. Cerebral autoregulation in normal pregnancy and preeclampsia. *Obstet Gynecol.* 2013;122(5):1064-1069.

21. Nagueh SF SO, Appleton CP, Byrd BF 3rd, Dokainish H, Edvardsen T, Flachskampf FA, Gillebert TC, Klein AL, Lancellotti P, Marino P, Oh JK, Popescu BA, Waggoner

For peer review only



For peer review only - http://bmjopen.bmj.com/site/about/guidelines.xhtml

AD. Recommendations for the Evaluation of Left Ventricular Diastolic Function by Echocardiography: An Update from the American Society of Echocardiography and the European Association of Cardiovascular Imaging. *Journal Am Soc Echocardiogr.* 2016;29(4):277-314.

22. Meeme A BG, Mammen M, Namugowa A. Endothelial dysfunction and arterial stiffness in pre-eclampsia demonstrated by the EndoPAT method. *Cardiovascular Journal of Africa.* 2016;27:1-6.

23. Capeless EL CJ. Cardiovascular changes in early phase of pregnancy. *American Journal of Obstetrics and Gynecology.* 1989;161(6):1449-1453.

24. RJ L. Thrombelastography/thromboelastometry. *Clin Lab Haematol.* 2005;27(2):81-90.

25. Moenen FCJI VM, Nelemans PJ, van Rooy KJM, Vranken JRRA, Verhezen PWM, Wetzels RJH, Ten Cate H, Schouten HC, Beckers EAM, Henskens YMC. Screening for platelet function disorders with Multiplate and platelet function analyzer. *Platelets.* 2019;30(1):81-87.

26. Love J, Moore CD, Hensing G. Validation of the Swedish translation of the General Self-Efficacy scale. *Qual Life Res.* 2012;21(7):1249-1253.

27. Spinhoven PH OJ, Sloekers PPA, Kempen GIJM, Speckens AEM, Van Hemert AM. A validation study of the Hospital Anxiety and Depression Scale (HADS) in different groups of Dutch subjects. *Psychological Medicine.* 1997;27:363-370.

28. Cox JL HJ, Sagovsky R. Detection of Postnatal Depression Development of the 10-item Edinburgh Postnatal Depression Scale. *British Journal of Psychiatry.* 1987;150:782-786.

29. Group TE. EuroQol - a new facility for the measurement of health-related quality of life. *Health Policy.* 1990;16:199-208.



For peer review only - http://bmjopen.bmj.com/site/about/guidelines.xhtml

For peer review only

30. Dencker A, Taft C, Bergqvist L, Lilja H, Berg M. Childbirth experience questionnaire (CEQ): development and evaluation of a multidimensional instrument. *BMC Pregnancy Childbirth.* 2010;10:81.

31. Gerhardsson E, Nyqvist KH, Mattsson E, Volgsten H, Hildingsson I, Funkquist EL. The Swedish Version of the Breastfeeding Self-Efficacy Scale-Short Form: Reliability and Validity Assessment. *J Hum Lact.* 2014;30(3):340-345.

32. Blevins CA, Weathers FW, Davis MT, Witte TK, Domino JL. The Posttraumatic Stress Disorder Checklist for DSM-5 (PCL-5): Development and Initial Psychometric Evaluation. *J Trauma Stress.* 2015;28(6):489-498.

33. Patton M. *Qualitative Research& Evaluation Methods (3rd ed.).* Thousand Oaks,CA,: Sage Publications; 2002.

34. Graneheim U LB. Qualitative content analysis in nursing research: Concepts, procedures and measures to achieve trustworthiness. *Nurse Education Today.* 2004;24:105-112.

35. Polit D BC. *Nursing Research, Generating and Assessing Evidence fo Nursing Practice.* 8th ed: Wolters Kluwer/Lippincott Williams&Wilkins; 2008.

36. Dahlberg K DH, Nyström Pettersson M. *Reflective Lifeworld Research.* Studentlitteratur Lund; 2008.

37. Elo S KH. The qualitative content analysis process. *Journal of Advanced Nursing.* 2008;62(1):107-115.

38. Virhammar J, Laurell K, Ahlgren A, Larsson EM. Arterial Spin-Labeling Perfusion MR Imaging Demonstrates Regional CBF Decrease in Idiopathic Normal Pressure Hydrocephalus. *AJNR Am J Neuroradiol.* 2017;38(11):2081-2088.

For peer review only



For peer review only - http://bmjopen.bmj.com/site/about/guidelines.xhtml

39. Elharram M, Dayan N, Kaur A, Landry T, Pilote L. Long-Term Cognitive Impairment After Preeclampsia: A Systematic Review and Meta-analysis. *Obstet Gynecol.* 2018;132(2):355-364.

40. Sacristan JA, Aguaron A, Avendano-Sola C, et al. Patient involvement in clinical research: why, when, and how. *Patient Prefer Adherence.* 2016;10:631-640.

41. Thangaratinam S, Allotey J, Marlin N, et al. Prediction of complications in early-onset pre-eclampsia (PREP): development and external multinational validation of prognostic models. *BMC Med.* 2017;15(1):68.

42. Cerdeira AS, O'Sullivan J, Ohuma EO, et al. Randomized Interventional Study on Prediction of Preeclampsia/Eclampsia in Women With Suspected Preeclampsia. *Hypertension.* 2019:HYPERTENSIONAHA11912739.

43. Brewer J, Owens MY, Wallace K, et al. Posterior reversible encephalopathy syndrome in 46 of 47 patients with eclampsia. *Am J Obstet Gynecol.* 2013;208(6):468 e461-466.

44. Fisher N, Saraf S, Egbert N, Homel P, Stein EG, Minkoff H. Clinical Correlates of Posterior Reversible Encephalopathy Syndrome in Pregnancy. *J Clin Hypertens (Greenwich).* 2016;18(6):522-527.

45. Aukes AM, De Groot JC, Wiegman MJ, Aarnoudse JG, Sanwikarja GS, Zeeman GG. Long-term cerebral imaging after pre-eclampsia. *BJOG.* 2012;119(9):1117-1122.

46. Bergman L, Zetterberg H, Kaihola H, Hagberg H, Blennow K, Akerud H. Blood-based cerebral biomarkers in preeclampsia: Plasma concentrations of NfL, tau, S100B and NSE during pregnancy in women who later develop preeclampsia - A nested case control study. *PLoS One.* 2018;13(5):e0196025.

47. Evers KS, Atkinson A, Barro C, et al. Neurofilament as Neuronal Injury Blood Marker in Preeclampsia. *Hypertension.* 2018;71(6):1178-1184.

For peer review only



For peer review only - http://bmjopen.bmj.com/site/about/guidelines.xhtml

48. Basit S, Wohlfahrt J, Boyd HA. Pre-eclampsia and risk of dementia later in life: nationwide cohort study. *BMJ.* 2018;363:k4109.

49. Miller EC. Preeclampsia and Cerebrovascular Disease. *Hypertension.* 2019;74(1):5-13.

50. Nerenberg KA, Park AL, Vigod SN, et al. Long-term Risk of a Seizure Disorder After Eclampsia. *Obstet Gynecol.* 2017;130(6):1327-1333.

51. Aukes AM, Wessel I, Dubois AM, Aarnoudse JG, Zeeman GG. Self-reported cognitive functioning in formerly eclamptic women. *Am J Obstet Gynecol.* 2007;197(4):365 e361-366.

52. Postma IR, Groen H, Easterling TR, et al. The brain study: Cognition, quality of life and social functioning following preeclampsia; An observational study. *Pregnancy Hypertens.* 2013;3(4):227-234.

53. Sunderland N, Hennessy A, Makris A. Animal models of pre-eclampsia. *Am J Reprod Immunol.* 2011;65(6):533-541.

For peer review only



For peer review only - http://bmjopen.bmj.com/site/about/guidelines.xhtml

## Figure legends

**Figure 1.**

Flow chart of the study. Investigations and biological samples from inclusion to one year postpartum. CSF; Cerebrospinal fluid, MRI; Magnetic resonance imaging

For peer review only



For peer review only - http://bmjopen.bmj.com/site/about/guidelines.xhtml

**Authors' contributions**

LB and LT wrote the article. LB contributed with information about biosamples in GO PROVE, transcranial doppler examination, MRI examinations, structure of the online database and inclusion of women in GO PROVE. LT contributed with information about questionnaires, cognitive tests, handling of biosamples and structure of the online database. MA, JO and SET provided the methods and contributed to the sections regarding cardiac function and endothelial dysfunction. OK provided the methods and contributed to the sections regarding coagulation. VS contributed to the section about recruitment of the population. TS and SL contributed to the methods section about placental sampling. HE and KL provided the methods and contributed to the sections regarding women's experience of childbirth and questionnaires. KJ and TVV contributed to the sections about transcranial doppler and cerebral autoregulation. IE contributed to the section about recruitment of particpants in Uppsala (UPMOST). JW and IBB provided the methods section and contributed to the sections about MRI. ASN provided the methods section and contributed to the sections about cognitive function tests. AKW contributed to the section about recruitment in Uppsala and handling of biosamples.

All authors revised and approved the final version of the manuscript.

**Funding statement**

This study is supported by the Swedish Brain Fund, Märta Lundqvist's foundation, Jane and Dan Olsson's foundation, the Foundation of the Health and Medical care committee of the Region of Västra Götaland, Sweden (VGFOUREG851391), (VGFOUREG930918), Gothenburg Society of Medicine (GLS934555 and GLS878481), Sahlgrenska University Hospital, ALF Västra Götalands Region (ALFGBG-925851), Swedish Research Council (2020-01640), Swedish Society for Medical Research and Sahlgrenska University Hospital SU (2018-03591).



For peer review only - http://bmjopen.bmj.com/site/about/guidelines.xhtml

**Competing interest statement**

The authors declare that they have no competing interests.

For peer review only



For peer review only - http://bmjopen.bmj.com/site/about/guidelines.xhtml

For peer review only



For peer review only - http://bmjopen.bmj.com/site/about/guidelines.xhtml

**Table 1.** Neurological Symptoms at inclusion, at MRI brain and at follow up one year postpartum.

| |
|---|
| Edema (n) |
| Visual disturbances (b) |
| *No visual disturbances* (b) |
| *Blurred vision* (b) |
| *Double vision* (b) |
| *Temporary loss of vision* (b) |
| *Sensitivity to light* (b) |
| *Partial loss of vision or blind spots in normal field of vision* (b) |
| *Flickering lights* (b) |
| *Other* (n) |
| |
| Time of onset for visual disturbances (n) |
| *N/A (no visual disturbance)* (b) |
| |
| Headache (o) |
| Headache onset (o) |
| Epigastric/abdominal pain (o) |
| Tightness in chest (b) |
| Shortness of breath (b) |
| Focal neurological deficits (n) |
| |
| Tendon reflexes (n) |
| Nausea (b) |
| Vomit (b) |
| Form of confusion (b) |
| Twitching or jerking in arms and/or legs (b) |
| Has the woman's family/friends experienced the patient as not responding or staring blankly into space (b) |
| Difficulty concentrating (b) |
| Speech affected (b) |
| Hearing affected (b) |
| Mood changes (n) |
| Other mood changes than listed (Free text) |
| |
| *Before being diagnosed with PE/MRI scan/visit at 1 year postpartum did the woman* |
| -feel anxious (b) |
| -feel as if the end of the world was coming (b) |
| -experience severe dizziness (b) |
| -experience any weakness or paralysis (b) |
| -experience jitters/nervousness/nervous shaking (b) |
| -experienced any other symptoms not listed (b) |
| List other symptoms (FREE text) |

For peer review only

**Table 2.** Biochemical analyses specific for the study and in clinical routine when available.

| Prenatal screening | At inclusion | At delivery and Summary of analyses at discharge* |
|---|---|---|
| **If taken in clinical routine**<br>human chorionic gonadotropin) (c)<br>Oestriol (c)<br>Alpha-fetoprotein (c)<br>Inhibin A (c)<br>Pregnancy-associated plasma protein A (c)<br>Cell free fetal DNA (c) | **If taken in clinical routine**<br>Haemoglobin (c)<br>Haematocrit (c)<br>WCC (white cell count) (c)<br>Platelets (c)<br>Creatinine (c)<br>AST (aspartate transaminase) (c)<br>ALT (alanine transaminase) (c)<br>Total bilirubin (c)<br>Albumin (c)<br>LDH (lactate dehydrogenase) (c)<br>Haptoglobin (c)<br>APTT (activated partial thromboplastin time) (c)<br>PR (prothrombin ratio) (c)<br>INR (International normalize ratio) (c)<br>CRP (C reactive protein) (c)<br>Oxygen saturation (d)<br>Total leukocyte count (c)<br>Urinary prot/creat ratio (c)<br>Fibrinogen (c)<br>Antitrombin (c)<br><br>**Specific for the study**<br>ROTEM (b)<br>Multiplate (b)<br>NT-pro BNP (c)<br>Troponin (c) | **At delivery specific for the study**<br>ROTEM (b)<br>Multiplate (b)<br>Platelets (c)<br>Troponin (c)<br>NT pro BNP (c)<br><br>**Summary of analyses at discharge if taken in clinical routine**<br>Haemoglobin (g/L) (c)<br>Platelet count (x10^9/L) (c)<br>Urinary protein/creatinine ratio (mg/g) (c)<br>Creatinine (mmol/L) (c)<br>Aspartate transaminase (IU/L) (c)<br>Alanine transaminase (IU/L) (c)<br>GGT (IU/L) (c)<br>Total bilirubin (umol/L) (c)<br>Albumin (g/L) (c)<br>LDH(IU/L) (c)<br>Haptoglobin (mg/dL) (c)<br>APTT (sec) (c)<br>INR (c)<br>D-dimer (ug/L) (c)<br>CRP (mg/L) (c) |

**Lowest antepartum in hospital/Highest antepartum in hospital/Lowest value postpartum/Highest value postpartum*

For peer review only

**Table 3.** Biological sample collection

| At inclusion | At delivery | At MRI brain | At one year postpartum |
|---|---|---|---|
| Serum | Serum | Serum | Serum |
| EDTA plasma | EDTA plasma | EDTA plasma | EDTA plasma |
| Li/Heparin plasma | Li/Heparin plasma | | |
| EDTA whole blood | EDTA plasma umbilical cord | | |
| Urine | Cerebrospinal fluid | | |
| | Placental villous tissue frozen | | |
| | Placental villous tissue RNA later | | |
| | Basal plate, full thickness frozen | | |
| | Basal plate, full thickness RNA later | | |

For peer review only

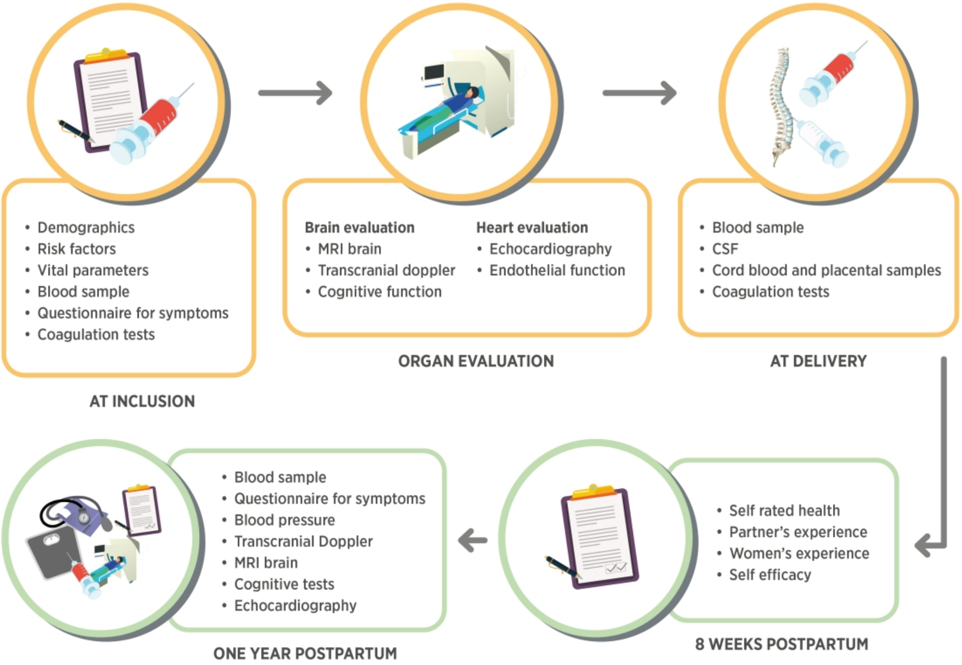


Flow chart of the study. Investigations and biological samples from inclusion to one year postpartum. CSF; Cerebrospinal fluid, MRI; Magnetic resonance imaging

297x209mm (300 x 300 DPI)

**Table S1**. Variables included in the study.

| Variables from the woman's medical chart | Variables from the study inclusion visit, gathered from the patients´ medical charts | Variables from the study inclusion visit when diagnosed with preeclampsia, registered by patients in the enrolment module | Variables from the Swedish Pregnancy Register after delivery |
|---|---|---|---|
| Marital status (n)<br>Current living situation (n)<br>Country of birth (n)<br>Total years of education (c)<br>Profession (n)<br>Maternal occupation in the beginning of pregnancy (b)<br>Height (c)<br>Intra uterine growth restriction (b)<br>Preeclampsia (b)<br><br>**Obstetric history**<br>Number of pregnancies terminated before 21+6 weeks gestation (d)<br>Previous pregnancies, terminated before 21+6 weeks gestation, causes (n)<br>Gestational age (d)<br>Gestational hypertension (b)<br>Preeclampsia (b)<br>Eclampsia (b)<br>HELLP (b)<br>Gestational diabetes treated with Insulin or oral agents (b)<br>Treatment details (Free text)<br><br>Number of pregnancies beyond 21+6 weeks gestation (d)<br>Information on previous pregnancies, with delivery beyond 21+6 weeks gestation, outcome details:<br>Gestational age (d)<br>Gestational hypertension (b)<br>Preeclampsia (b)<br>Eclampsia (b)<br>HELLP (b) | **Study assessment at inclusion**<br>Date (c)<br>Location (n)<br>1st BP (c)<br>2nd BP (c)<br>Pulse (c)<br>Weight (c)<br>Proteinuria (dipstick) (o)<br>Urinary protein/creatinine ratio (c)<br>24 hour protein (c)<br>Gestation at PE/Intra uterine growth restriction (IUGR) diagnosis (c)<br><br>**Investigations at inclusion**<br>Samples taken, see table 3<br><br>**Urine sample**<br>See Table 3<br><br>**Gestational diabetes (GDM) screening, diagnosis treatment**<br>Date screened (c)<br>Date of GDM diagnosis (c)<br>Gestation GDM diagnosed (c)<br>Date of glucose tolerance test (d)<br><br>**Ultrasound and doppler scan after preeclampsia diagnosis**<br>Date of scan (d)<br>Fetal sex (n)<br>Gestational age when scanned (c)<br>Fetal abnormality (n)<br>Crown rump length (c)<br>Nuchal translucency (c) | Yearly income (o)<br>First pregnancy with the baby's biological father (b)<br>Months of sexual relationship (pre-pregnancy) (c)<br>Contraception used in this relationship (n)<br>If contraception has been used, which mostly in the last year (n)<br>Medications before pregnancy (n)<br>First degree relative/s with hypertensive disease in pregnancy (b)<br>First degree relative/s with hypertension, Ischaemic Heart Disease, Stroke or any form of Diabetes (b)<br>Pre-pregnancy weight (c)<br><br>**Partners Family History**<br>Children with other partners (b)<br>Pregnancy outcome with other partner (n)<br>First degree relative with preeclampsia or Eclampsia (b)<br>First degree relative/s with Hypertension (b), Ischaemic Heart Disease (b), Stroke (b) or any form of Diabetes (b)<br><br>**Partner's Data**<br>Age (c)<br>Weight (c)<br>Height (c)<br>Body mass index (c)<br>Partners medical condition (n)<br>Diabetes mellitus (b)<br>Hypertension (b)<br>Chronic Heart Disease (b) | **Maternal**<br>*Delivery data*<br>Date of admission pre-delivery (c)<br>GA at admission pre-delivery (c)<br>Onset of delivery (n)<br>Maximum temperature in labour (c)<br><br>Foetal compromise pre-labour Caesarean section<br>Place and supervision of delivery (n)<br>Duration of rupture of membranes (c)<br><br>Duration of 1st stage (c)<br>Duration of 2nd stage (c)<br><br>Mode of delivery (n)<br>Indication for operative delivery (n)<br>Indication for delivery (n)<br><br>*Medication*<br>Magnesium sulphate (n)<br>Steroids (n)<br>Antihypertensive treatment in labour (n)<br>Medication during labour and delivery only (Free text)<br>Calcium antagonist (b)<br><br>*Placenta*<br>Recommended standards of placental handling used (b)<br>Placental weight (c)<br>Cord insertion relative to placental margin (n)<br>Number of vessels in cord (b)<br>Pathology report (b) |

For peer review only

| | | | |
|---|---|---|---|
| Gestational diabetes treated with Insulin or oral agents (b)<br>Treatment details (n)<br><br>**History of infertility** (b)<br>Fertility treatment for this conception (b)<br>Stimulation for ovulation for this conception (b)<br>Artificial insemination for this conception (n)<br>In Vitro fertilization for this conception (n)<br>Intra Cytoplasmic sperm injection for this conception (n)<br>Embryo recipient for this conception (b)<br><br>**Medical conditions diagnosed before pregnancy**<br>Polycystic ovary syndrome (b)<br>Medical conditions diagnosed before pregnancy (n)<br>Hypertension (b)<br>Renal disease (b)<br>Anaemia (b)<br>Diabetes type I (b)<br>Diabetes type II (b)<br>Lupus disease (b)<br>Inflammatory bowel disease (b)<br>Coeliac disease (b)<br>Rheumatoid arthritis (b)<br>Epilepsy (b)<br>Thromboembolism (b)<br>History of depression (b)<br>Antiphospholipid syndrome (b)<br>HIV (b)<br>Sickle cell (b)<br>Malaria requiring treatment in the last year (b)<br>Asthma (b)<br>Oral steroids for asthma (b)<br>Thyroid disease (n)<br>Migraine (b)<br>Irritable colon (b)<br>Anxiety disorder (b)<br>Other medical conditions diagnosed before pregnancy (free text) | Biparietal diameter (BPD (c)<br>Head circumference (HC) (c)<br>Abdominal diameter (AD (c)<br>Femur length (C)<br>Sub-chorionic haematoma (b)<br>2nd sac (b)<br>Risk score of Trisomy 21(d)<br>Liquor volume (o)<br>Placental Location (n)<br>Clinical suspicion for fetal growth restriction (b)<br>Documented fetal growth restriction (b)<br>Clinical indication for Macrosomia (b)<br>Amniotic fluid index (c)<br>Single deepest pocket (c)<br><br>*Umbilical dopplers* (c)<br>Gestational age (c)<br>Umbilical PI (c)<br><br>*Uterine dopplers*<br>L Ut Pulsatility Index (c)<br>R Ut Pulsatility Index (c)<br>Mean Ut PI (c)<br>L Notch (c)<br>R Notch (c)<br>Other Ultrasound comments (Free text)<br>Fetal Outcome (n)<br><br>**Medication use at inclusion**<br>Any medication at inclusion(b)<br>Low dose aspirin (b)<br>Other antiplatelet medication (b)<br>Low molecular weight heparin (n)<br>Diuretics (n)<br>Labetalol (n)<br>Beta blocking agent (n)<br>Calcium channel antagonist (n)<br>Antibiotic (n)<br>Anticoagulants (n)<br>Anticonvulsant-1 (not $MgSO_4$) (n)<br>Antidepressants (n) | Other (Free text)<br><br>**Vitamin and mineral supplementation before /and during pregnancy**<br>Folate (c)<br>Multivitamin (o)<br>Vitamin D (o)<br>Iron (o)<br>Calcium (o)<br><br><br>**Infection in pregnancy to record** (b)<br>Type of infection (n)<br>Urinary tract infection (lower) (b)<br>Pyelonephritis (b)<br>Human immunodeficiency virus (b)<br>Active Tuberculosis (b)<br>Zika virus (b)<br>Schistosomiasis (b)<br>Active or chronic hepatitis (b)<br>Active or recently treated sexually transmitted infection (b)<br>Other infection in pregnancy (b)<br><br>Diagnosis (n)<br>Treatment (n)<br><br>**Symptoms at inclusion see Table X**<br><br>**At time of MRI brain and Cognitive tests**<br>Blood pressure (c)<br>Pulse (c)<br>Oxygen saturation (d)<br><br>Current treatment<br>-Magnesium sulphate IVI (b)<br>-Labetolol IVI (b)<br>-Hydralazine IVI (b)<br>-Calcium antagonist short acting oral (b)<br>-Labetolol oral (b)<br>-Calcium antagonist long acting oral (b) | Photograph against a scale bar (b)<br><br>**Newborn data**<br>Liveborn (b)<br>Date of birth (c)<br>Time of birth (c)<br>Gestational age at delivery (c)<br>Sex (b)<br>Apgar score (d)<br>Intubation at birth (n)<br>Cord arterial pH (c)<br>Cord arterial Base deficit (c)<br>Cord venous pH (c)<br>Cord venous Base deficit (c)<br>Birthweight (c)<br>Birthweight centile (c)<br>Customised birthweight centile (c)<br>Length (c)<br>Head circumference (c)<br>TSH (c)<br>T3 total (c)<br>T4 total (c)<br>T3 free (c)<br>T4 free (c)<br>ICD code(s) for measures and diagnosis (n/free text)<br><br>*Neonatal Outcome at discharge*<br>Uncomplicated neonatal outcome (b)<br>Neonatal outcome (n)<br>Date of discharge home (c)<br>Days in hospital from birth to discharge home (d)<br>Admission to neonatal unit (c)<br>Final discharge date from neonatal unit or date of death in neonatal unit (c)<br>Days in neonatal unit (d)<br><br>**Perinatal outcome**<br>Perinatal Survival (b)<br>Stillbirth (b) |

For peer review only

| | | | |
|---|---|---|---|
| Medications before pregnancy (n)<br><br>**Last menstrual period** (LMP)<br>Certainty (n)<br>LMP date (c)<br>Gestational age by LMP/clinical examination (c)<br>Estimated due date by LMP (c)<br><br>***Estimated due date estimated by scan***<br><16 week scan date (c)<br>19-21 week scan date (c)<br>Final estimated due date (c)<br><br>**Clinical Readings at 1st Clinical Assessment**<br>Date of 1st clinical blood pressure (BP) (c)<br>Gestation of 1st BP (c)<br>1st clinical weight (c)<br>Proteinuria dipstick (o)<br>Urinary protein/creatinine ratio (c)<br>24 hour protein (c)<br><br>**Clinical Test**<br>(*Prenatal screening for chromosomal anomalies*)<br>Prenatal diagnosis (n)<br>Serum Screening for prenatal diagnosis (SSPD) (b)<br>Date of SSPD performed (c)<br>Gestation if SSPD performed (c)<br><br>**1st and 2nd Trimester Complications**<br>Hyperemesis (b)<br>Treatment in case of hyperemesis (free text)<br>Vaginal bleeding (b)<br>Gestational week if vaginal bleeding has occurred (c)<br>Duration of bleeding (d)<br>Amount of blood (o) | Antiglycemic agent –Insulin (b)<br>Metformin (b)<br>Long-term corticosteroids (b)<br>Other immunosuppressant (n)<br>Thyroid supplement (b)<br>Antithyroid treatment for thyrotoxicosis (b)<br>Statins (n)<br>Other medication(n)<br>Hydralazine (b)<br>Magnesium sulphate (b)<br><br>Received steroids for fetal lung maturation before sampling (b)<br>Type of steroids (n)<br>Date started (c)<br>Gestational age started (c)<br>Date completed (c)<br>Gestational age completed (c)<br>Total dose (c)<br>Time between last dose and sampling (c) | -ACE inhibitor oral (b)<br>-AT-2 inhibitor oral (b)<br>-Diuretics IVI (b)<br>-Diuretics oral (b)<br>-Other medications (b)<br><br>**Neurological symptoms at MRI brain, see table X**<br><br>**Echocardiocraphy and RH pat**<br>Date (c)<br>Blood pressure (c)<br>Pulse (c)<br>Oxygen saturation (d)<br>Oxygen administration (b)<br>Current treatment:<br>-Magnesium sulphate IVI (b)<br>-Labetolol IVI (b)<br>-Hydralazine IVI (b)<br>-Calcium antagonist short acting oral (b)<br>-Labetalol (b)<br>-Calcium antagonist long acting oral (b)<br>-ACE inhibitor oral (b)<br>-AT-2 inhibitor oral (b)<br>-Diuretics IVI (b)<br>-Diuretics oral (b)<br>-Other medications (b)<br>If *other*, state (FREE text)<br><br>**Variables from the visit at 1 year postpartum registered by the patient in the study module as well as study personnel**<br>Date (date)<br>Blood pressure (c)<br>Pulse (c)<br>Weight (c)<br>*Medication*<br>ACE inhibitor (b)<br>AT-2 inhibitor (b)<br>Diuretics (b) | Neonatal death (o)<br><br>**Final Maternal and Perinatal Outcome**<br>Maternal outcome (n)<br>Maternal admission to intensive care unit (b)<br><br>**Pregnancy Summary & Complication**<br>Did any of these complications occur<br>-Normal pregnancy (b)<br>-Preeclampsia (b)<br>-Gestational Hypertension (b)<br>-Eclampsia or HELLP (b)<br>-Chronic hypertension (b)<br>-Placental abruption (b)<br>-Maternal death (b)<br>-Small for gestational age by population centile (b)<br>-Fetal growth retardation (b)<br>-Spontaneous preterm birth (b)<br>-PPROM with spontaneous preterm birth (b)<br>-Cervical insufficiency (b)<br>-Pregestational diabetes (b)<br>-Gestational diabetes (b)<br>-Cholestasis of pregnancy (b)<br>-Venous thromboembolism (b)<br>-Obese (BMI≥30) (b)<br>-Postpartum haemorrhage (PPH) (b)<br>-Other complications (b)<br>Maternal outcome comment (Free text)<br>-Perinatal outcome (n)<br>Fetal outcome comment (Free text)<br>Autopsy examination (n)<br>Major congenital abnormalities (n)<br>Chromosomal abnormality (n)<br>Congenital infection (b)<br>*ICD codes for diagnosis*<br>O14.0 (b)<br>O14.1A (b)<br>O14.1B (b)<br>O14.1X (b)<br>O14.9 (b) |

For peer review only

| | | | |
|---|---|---|---|
| **Recreational Drug use** | | Betablocker (b) | O11.9 (b) |
| Any recreational drug user (n) | | Aspirin (b) | Other (b) |
| Gestation ceased (c) | | Insulin (b) | Procedure codes (FREE text) |
| Tobacco (o) | | Oral diabetes medication (b) | |
| Cigars (o) | | Lipid lowering medication (b) | **Other severe complication at any time before** |
| Chewing tobacco (o) | | Levothyroxine (b) | **or after delivery** |
| Snuff (o) | | Antidepressants (b) | -None (b) |
| E-cigarettes (o) | | Sleep medicine (b) | -Unknown (b) |
| Nicotine replacement (o) | | | -DIC (b) |
| Alcohol (o) | | Did she breastfeed (b) | -Acute renal insufficiency (b) |
| | | Period of breastfeeding (o) | -No urine catheter output for more than 60 |
| *Other drugs in pregnancy* | | Regained period (b) | min (b) |
| Marijuana (b) | | Use of contraception (b) | -Haemolysis (b) |
| Cocaine/Crack (b) | | -If yes, what contraception (free text) | -CVA (b) |
| Amphetamines (b) | | | -Epigastric pain (b) |
| Substance P/Crystal Meth (b) | | After delivery, has the woman sought | -Pulmonary Oedema (b) |
| XTC (b) | | healthcare for; | -Hepatic rupture (b) |
| Opiates (b) | | -Migraine (b) | -Severe sepsis (b) |
| Hallucinogens (b) | | -High blood pressure (b) | -Maternal death (b) |
| Other (n) | | -Visual disturbance (b) | -Foetal death (b) |
| | | -Diabetes (b) | -Thrombocytopenia (b) |
| Audit number 3 months prior to pregnancy (d) | | -Thyroid function disorder (b) | -Abnormal LFT (due to PE) (b) |
| | | -Allergies (b) | -Coma (b) |
| **Ultrasound** | | -Irritable colon (b) | -Jaundice (b) |
| Date of scan (c) | | -Alcohol or drug addiction (b) | -Respiratory failure (b) |
| Fetal sex (n) | | -Depression (b) | -Cardiac failure (b) |
| Gestational age (c) | | -Anxiety (b) | -Venous Thromboembolism (b) |
| Fetal abnormality(n) | | -Pain-related disorders (b) | -Other complications (b) |
| CRL (crown rump length) (c) | | | -If other, specify (FREE text) |
| BPD (biparietal diameter) (c) | | Cognitive test performed (b) | |
| HC (head circumference) (c) | | -STAI, PANAS, PSQI performed (b) | |
| AD (abdominal diameter) (c) | | -EPDS, HADS performed (b) | **Summary of antenatal and postnatal lab** |
| FL (femur length) (c) | | -EQ-VAS, CEC, BSES, GES performed (b) | **results** |
| Sub-chorionic haematoma(b) | | -Bioimpedance been performed (b) | -Blood group (n) |
| $2^{nd}$ sac (b) | | | -Rhesus factor (b) |
| Risk score of Trisomi 21(d) | | **Neurological symptoms at 1 year** | Table Y |
| Liquor Volume (o) | | **postpartum, see Table X** | |
| Placental Location (n) | | | **End report-status** |
| Clinical suspicion for fetal growth restriction (FGR) | | | Final pregnancy outcome (n) |
| (b) | | | |
| Documented fetal growth restriction (b) | | | If pregnancy ended $\leq 19^{+6}$, date (c) |

For peer review only

| | | | |
|---|---|---|---|
| Clinical indication for Macrosomia (b)<br>Amniotic fluid index (AFI) (c)<br>Single deepest pocket (c)<br><br>*Umbilical dopplers*<br>Gestational age (c)<br>Umbilical RI (resistive index) (n)<br>Umbilical PI (pulsatility index) (n)<br>Umbilical vein (n)<br><br>*Uterine dopplers*<br>L Ut PI (n)<br>R Ut PI (n)<br>Mean Ut PI (n)<br>L notch (n)<br>R notch (n)<br>Foetal outcome (n) | | | Gestational age at pregnancy end (c)<br><br>**MRI brain 1 year postpartum**<br>Blood pressure (c)<br>Pulse (c)<br>Plasma sample taken (b) |

For peer review only

STROBE Statement—Checklist of items that should be included in reports of ***cohort studies***

| | Item No | Recommendation |
|---|---|---|
| **Title and abstract** | 1 | (*a*) Indicate the study's design with a commonly used term in the title or the abstract<br>Title page |
| | | (*b*) Provide in the abstract an informative and balanced summary of what was done and what was found<br>Page 3-4 |
| **Introduction** | | |
| Background/rationale | 2 | Explain the scientific background and rationale for the investigation being reported<br>Page 6-7 |
| Objectives | 3 | State specific objectives, including any prespecified hypotheses<br>Page 7-8 |
| **Methods** | | |
| Study design | 4 | Present key elements of study design early in the paper<br>Page 7 |
| Setting | 5 | Describe the setting, locations, and relevant dates, including periods of recruitment, exposure, follow-up, and data collection<br>Page 7, 9-14 |
| Participants | 6 | (*a*) Give the eligibility criteria, and the sources and methods of selection of participants. Describe methods of follow-up<br>Page 9 and Figure 1 |
| | | (*b*) For matched studies, give matching criteria and number of exposed and unexposed<br>N/A |
| Variables | 7 | Clearly define all outcomes, exposures, predictors, potential confounders, and effect modifiers. Give diagnostic criteria, if applicable<br>Page 8. The study is a biobank and database that will also address future research questions and outcomes. |
| Data sources/ measurement | 8* | For each variable of interest, give sources of data and details of methods of assessment (measurement). Describe comparability of assessment methods if there is more than one group<br>Page 9-14 |
| Bias | 9 | Describe any efforts to address potential sources of bias<br>Page 15 |
| Study size | 10 | Explain how the study size was arrived at<br>Page 15-16 |
| Quantitative variables | 11 | Explain how quantitative variables were handled in the analyses. If applicable, describe which groupings were chosen and why<br>N/A, study protocol |
| Statistical methods | 12 | (*a*) Describe all statistical methods, including those used to control for confounding<br>Page 15 |
| | | (*b*) Describe any methods used to examine subgroups and interactions<br>N/A |
| | | (*c*) Explain how missing data were addressed<br>N/A, study protocol |
| | | (*d*) If applicable, explain how loss to follow-up was addressed<br>N/A, study protocol |
| | | (*e*) Describe any sensitivity analyses |

For peer review only

| | | |
|---|---|---|
| | | N/A |
| **Results** | | |
| Participants | 13* | (a) Report numbers of individuals at each stage of study—eg numbers potentially eligible, examined for eligibility, confirmed eligible, included in the study, completing follow-up, and analysed<br>N/A, study protocol |
| | | (b) Give reasons for non-participation at each stage<br>N/A, study protocol |
| | | (c) Consider use of a flow diagram<br>N/A, study protocol |
| Descriptive data | 14* | (a) Give characteristics of study participants (eg demographic, clinical, social) and information on exposures and potential confounders<br>N/A, study protocol |
| | | (b) Indicate number of participants with missing data for each variable of interest<br>N/A, study protocol |
| | | (c) Summarise follow-up time (eg, average and total amount)<br>N/A, study protocol |
| Outcome data | 15* | Report numbers of outcome events or summary measures over time<br>N/A, study protocol |
| Main results | 16 | (*a*) Give unadjusted estimates and, if applicable, confounder-adjusted estimates and their precision (eg, 95% confidence interval). Make clear which confounders were adjusted for and why they were included<br>N/A, study protocol |
| | | (*b*) Report category boundaries when continuous variables were categorized<br>N/A, study protocol |
| | | (*c*) If relevant, consider translating estimates of relative risk into absolute risk for a meaningful time period<br>N/A, study protocol |
| Other analyses | 17 | Report other analyses done—eg analyses of subgroups and interactions, and sensitivity analyses<br>N/A, study protocol |
| **Discussion** | | |
| Key results | 18 | Summarise key results with reference to study objectives<br>N/A, study protocol |
| Limitations | 19 | Discuss limitations of the study, taking into account sources of potential bias or imprecision. Discuss both direction and magnitude of any potential bias<br>Page 16-18 |
| Interpretation | 20 | Give a cautious overall interpretation of results considering objectives, limitations, multiplicity of analyses, results from similar studies, and other relevant evidence<br>N/A, study protocol |
| Generalisability | 21 | Discuss the generalisability (external validity) of the study results<br>N/A, study protocol |
| **Other information** | | |
| Funding | 22 | Give the source of funding and the role of the funders for the present study and, if applicable, for the original study on which the present article is based<br>Page 28 |

*Give information separately for exposed and unexposed groups.

For peer review only

**Note:** An Explanation and Elaboration article discusses each checklist item and gives methodological background and published examples of transparent reporting. The STROBE checklist is best used in conjunction with this article (freely available on the Web sites of PLoS Medicine at http://www.plosmedicine.org/, Annals of Internal Medicine at http://www.annals.org/, and Epidemiology at http://www.epidem.com/). Information on the STROBE Initiative is available at http://www.strobe-statement.org.

For peer review only


For peer review only - http://bmjopen.bmj.com/site/about/guidelines.xhtml
